# Supplementary material for: BMP, MEK, and WNT inhibition with NGN2 expression for rapid generation of hiPSC-derived neurons amenable to regional patterning
Source: Stem Cell Reports. 2025 Jun 19;20(7):102539. doi: 10.1016/j.stemcr.2025.102539 (PMC12277818; doi:10.1016/j.stemcr.2025.102539)
Supplement: Document S1. Figures S1–S7, Tables S1–S9, Notes S1–S3, and supplemental methods [file mmc1.pdf]

Stem Cell Reports, Volume *20*

Supplemental Information

BMP, MEK, and WNT inhibition with NGN2 expression for rapid generation of hiPSC-derived neurons amenable to regional patterning

Carina Habich, Alexandra Kowalski, Astrid Wachter, Michaela J. Heimann, Michael Wolf, Markus P. Kummer, Nathalie Nicolaisen, Christopher Sliwinski, Lydia Reinhardt, Veronika Heil, Timo Lange, Christopher Untucht, Loan N. Miller, Jürgen Korffmann, Daniela Geist, David Schöndorf, Heyne Lee, Lamiaa Bahnassawy, Benjamin Mielich-Süss, Melanie S. Brennan, Ruven Wilkens, Julian Röwe, Ian Weidling, Rüdiger Rudolf, Mathias Hafner, Justine D. Manos, Miroslav Cik, and Peter Reinhardt

Supplementary Information

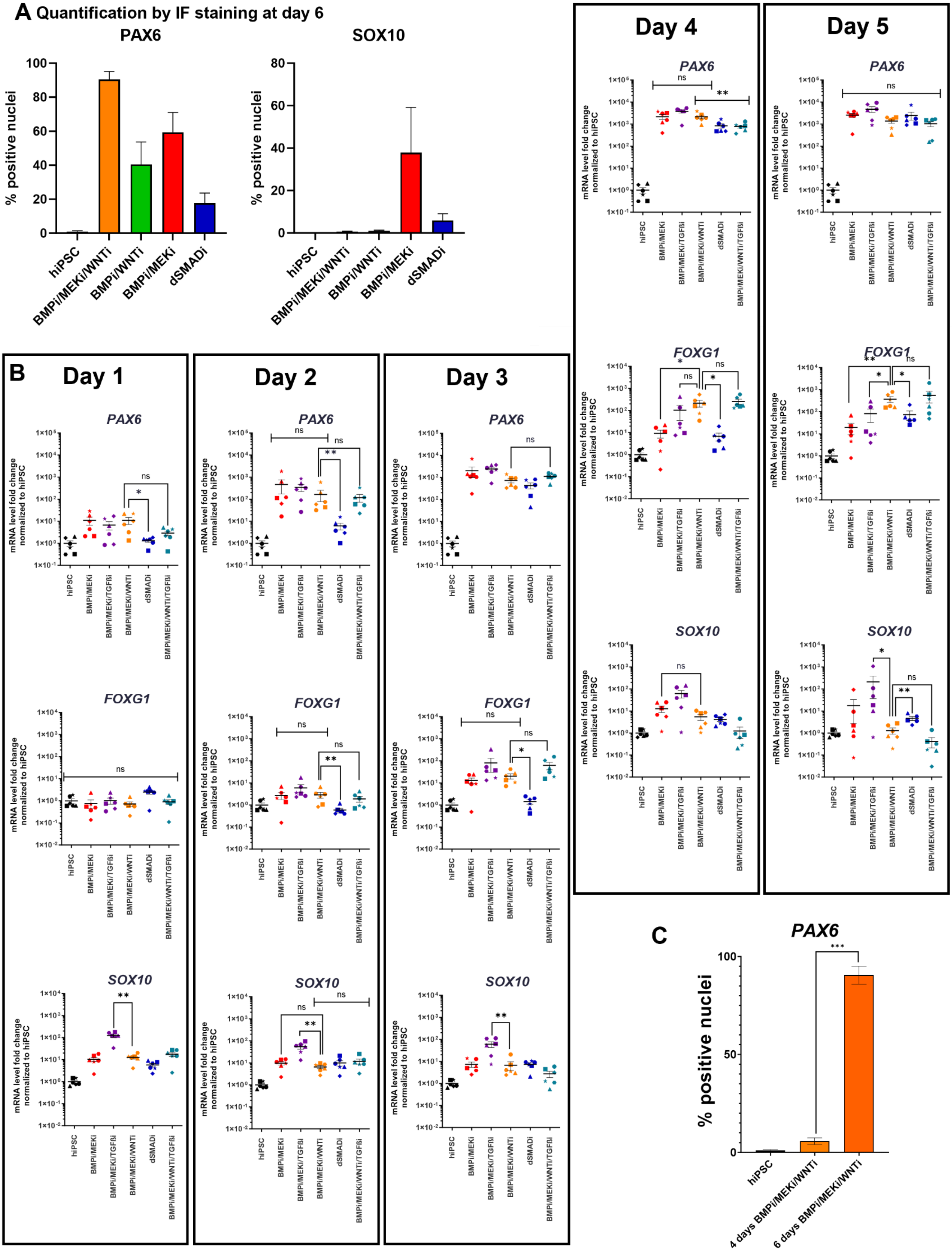

Figure S1: Additional data related to main-text figure 1. A Quantification of IF staining in Figure 1C. 5 different cell lines (hiPSC_1-5). **B** Time course of gene expression measured by qPCR after treatment of hiPSC with different combinations of inhibitors from day 0 to day 6 (results are shown as means ±SEM; N = 6 individual experiments with 6 different cell lines). In the main text Figure 1B, the course over 6 days is shown. The individual results of day 6 are shown in Figure 1D. In order to check significance, the replicates of each day were plotted individually and selected combinations were examined for significance. **C** Quantification of IF images Figure 1E (hiPSC_1-5). (* = $p < 0.05$, ** = $p < 0.01$; ●=hiPSC_1, ▲=hiPSC_2, ■=hiPSC_3, ♦=hiPSC_4, ★=hiPSC_5, ⬣=hiPSC_6)

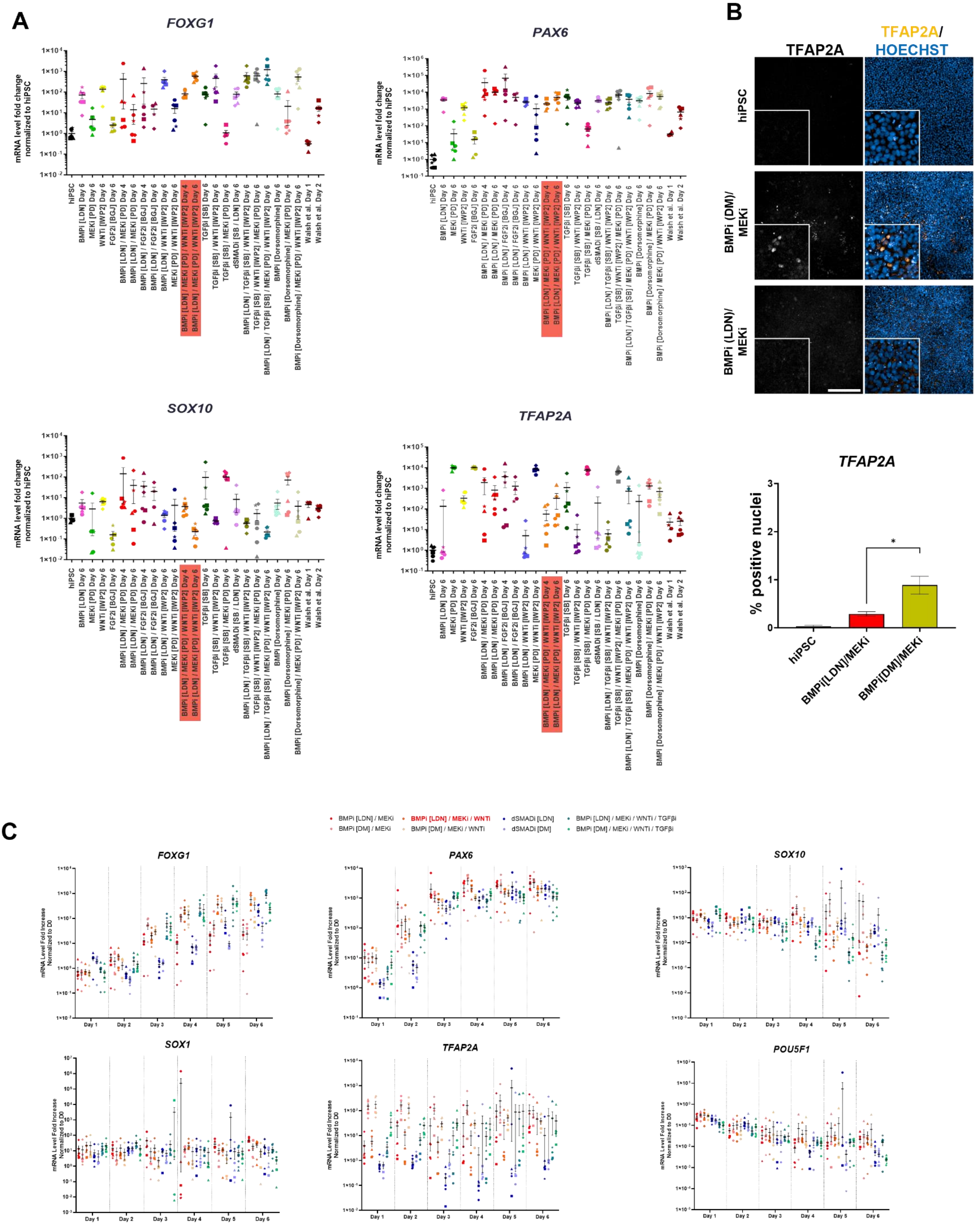

Figure S2: Additional data related to main-text Figure 1. A Gene expression after 4 or 6 days of treatment with different inhibitor combinations. 6 different cell lines (hiPSC_1-6) were used and a total of 6 independent differentiations were performed. For comparison, the differentiation protocol of Walsh et al. was replicated and samples were taken on day 1 and 2 (N=6 cell lines), (results are shown as means ±SEM). **B** Comparison of BMP

inhibition with LDN or DM. Inhibition with DM does not completely block formation of non-neural ectoderm, highlighted by the presence of TFAP2A+ cells. Representative IF pictures of hiPSC_1 were shown. Quantification of TFAP2A positive nuclei in 5 cell lines is shown (hiPSC_1-5, Scale bar: 200 µM, insert 3x zoom-in, means ±SEM). **C** Data points of time course of gene expression measured by qPCR after treatment of hiPSC (hiPSC_1-6) with different combinations of inhibitors from day 0 to day 6 (results are shown as means ±SEM; N = 6 different cell lines; DM=Dorsomorphine). (●=hiPSC_1, ▲=hiPSC_2, ■=hiPSC_3, ♦=hiPSC_4, ★=hiPSC_5, ⬣=hiPSC_6).

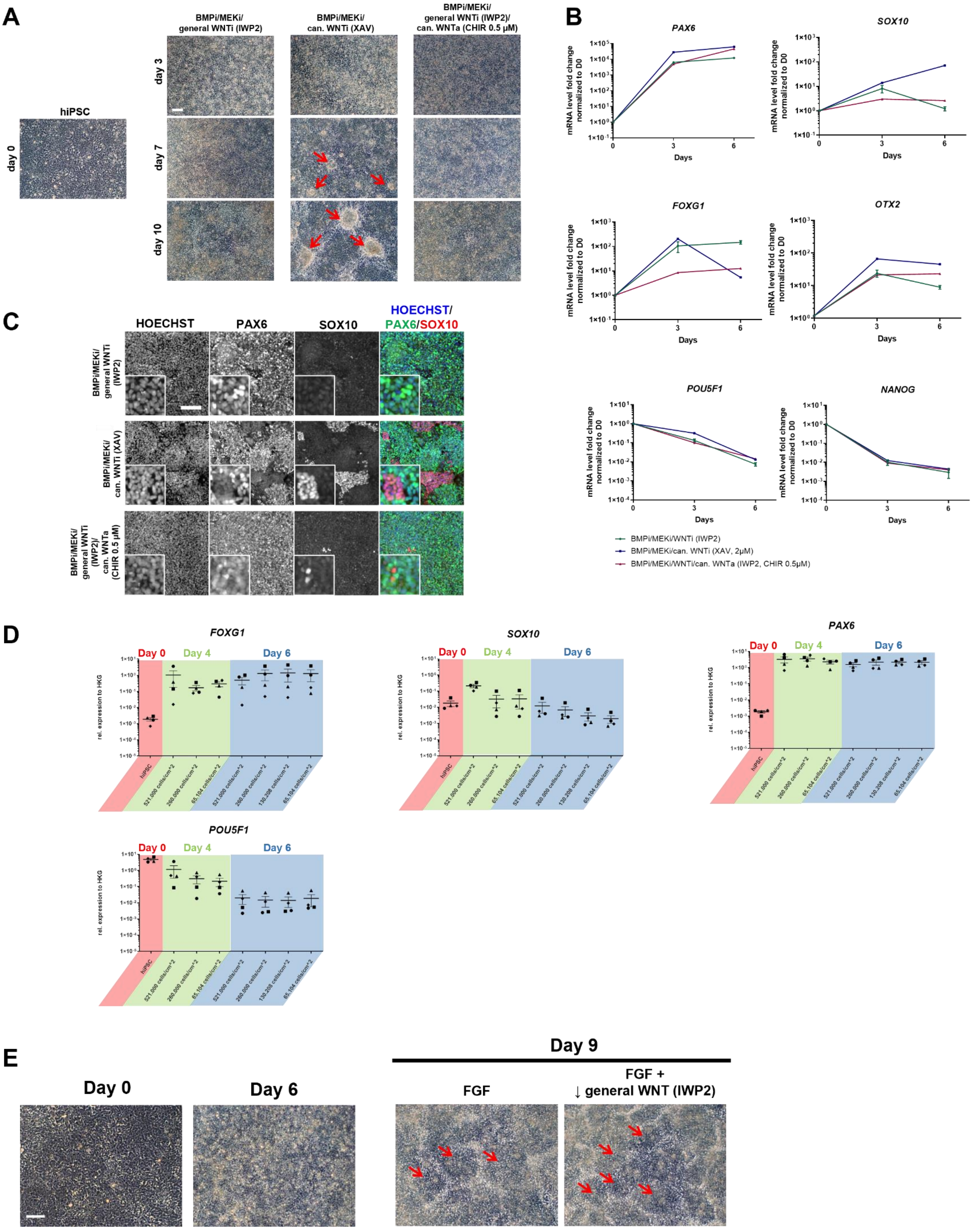

Figure S3: Additional data related to main-text Figure 1. A hiPSC_6 was treated with BMPi/MEKi and either with a general WNT inhibitor (IWP2), a canonical WNT inhibitor (XAV) or IWP2 with slight simultaneous canonical WNT activation (0.5 µM CHIR, GSK3 inhibitor). Phase contrast images of the cells at day 0, day 3, day 7 and day 10 of induction are shown. Sphere formation, signifying neural crest differentiation, is observed only when canonical WNT is inhibited without additional WNT inhibitors. Red arrows indicate spheres (Scale bar: 100 µM). **B** Gene analysis

by qPCR of relevant genes. Inhibition of canonical WNT results in the highest fold increase in *PAX6* and *SOX10*, along with a rapid downregulation of *FOXG1* starting from day 3 onwards. General WNT inhibition leads to the highest fold increase for *FOXG1* (1 hiPSC line was used N=1 (hiPSC_6), BMPi/MEKi/WNTi n=3 independent differentiations, other conditions n=1, results are shown as means ±SEM). **C** IF staining of cells (same experiment as in B) at day 6 of neural induction. Cells were stained for the neuroectodermal marker PAX6 and the neural crest marker SOX10. Patches of SOX10+ cells were observed only when canonical WNT was inhibited without additional WNT inhibitors. Individual SOX10+ cells are also present when general WNT is inhibited and the cells are treated with the canonical WNT activator CHIR. The images suggest that non-canonical WNT signaling has a more significant impact on neural crest formation than canonical WNT signaling (Scale bar: 100 µM, insert 3x zoom-in). **D** hiPSC_1-4 lines were differentiated up to day 6 using the BMWi protocol. On day 0 cells were seeded at different densities. Using qRT-PCR, the RNA samples from the hiPSC, the pre-differentiated precursors and the NPC were examined for relevant genes on the day of replating (N=4 cell lines, results are shown as means ±SEM). **E** hiPSC were induced for 6 days by BMWi. Subsequently, the cells were expanded for 4 days through treatment with FGF2 or a combination of FGF2 and general WNT inhibition (IWP2). Phase contrast images were taken on D0, D6 and D9. Representative images of hiPSC_6 are shown. After treatment with FGF2 formation of neural rosettes can be observed. Selected rosetted labeled by arrows (Scale bar: 100 µM).

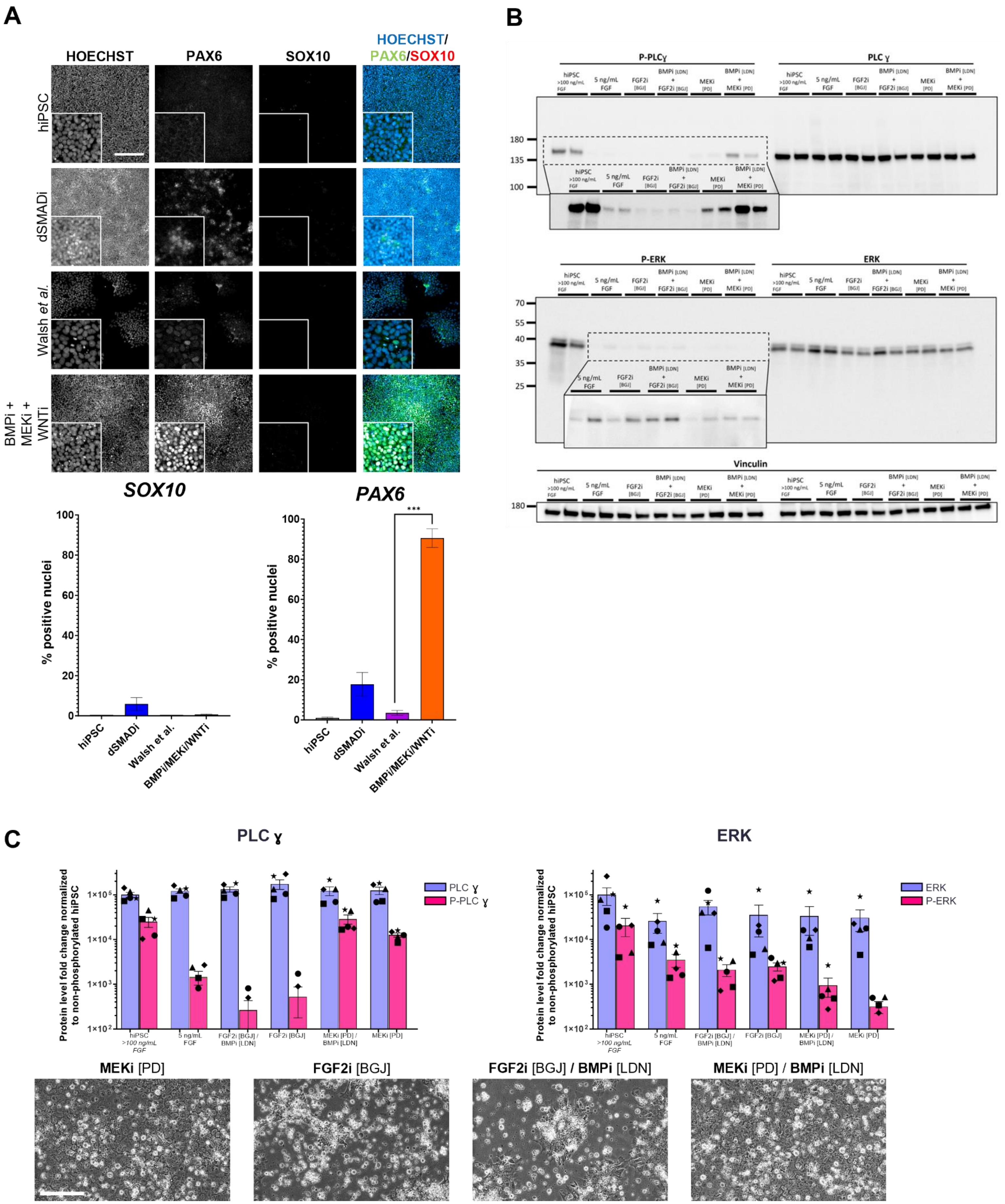

Figure S4: **Additional data related to main-text Figure 1. A** Representative immunofluorescence staining (hiPSC_5) after 6 days of treatment with various inhibitors. According to Walsh et *al.* differentiated cells were fixed on day 2 (final stage NPC) of the protocol instead (Scale bar: 200 µM, insert 3x zoom-in). A quantification of 5 cell lines was carried out (N = 5 cell lines, hiPSC_1-5, results are shown as means ±SEM). **B** hiPSC were treated with the indicated inhibitors for 4 days. As a positive control, 5 ng/ml FGF2 was added to FGF2-free medium, or hiPSC medium (100 ng/ml FGF2) was used. The results of 5 cell lines (hiPSC_1-5) were evaluated using western blot

(12 µg protein each well) with vinculin control. **C** Quantification of western blot results from B (N=5 cell lines). The positive control (>100 ng/ml FGF2) was set to 100% and the other samples were then normalized (results are shown as means ±SEM). Representative images show hiPSC_4 after 4 days of treatment with the specified inhibitors (scale bar: 200 µM).

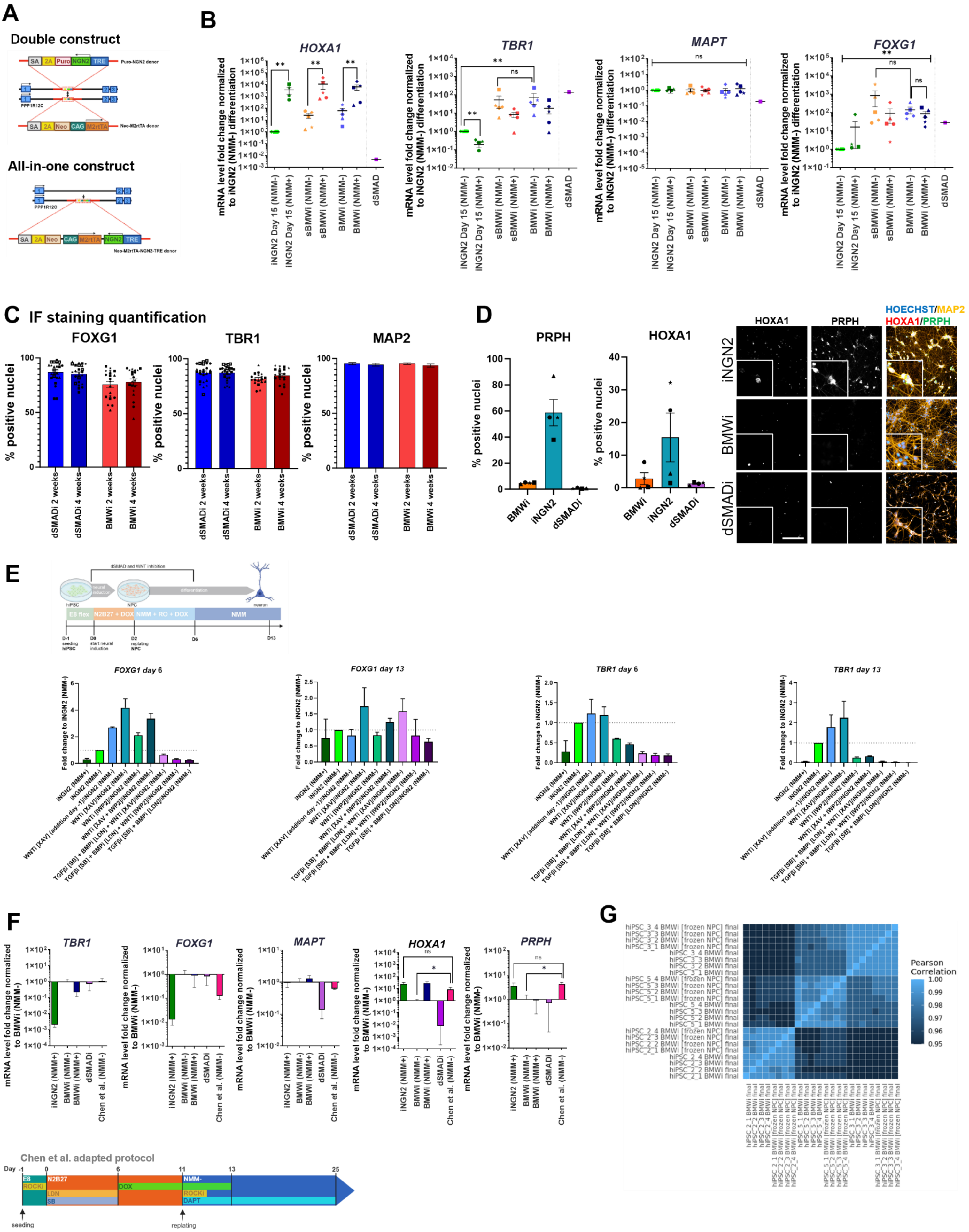

Figure S5: Additional data related to main-text Figure 1/2. A The overexpression of the basic helix-loop-helix transcription factor neurogenin 2 (NGN2) promotes the direct differentiation of hiPSC to neurons. In the ASSV1 (PPP1R12C) locus, puro-NGN2 and neo-M2rtTA were inserted on 2 alleles by gene editing through HDR pathway (hiPSC_1,2,4,5/ Double construct). Alternatively, an all-in-one construct was inserted into an allele in AAVS1

(hiPSC_3 - All-in-one construct). The expression of NGN2 is stimulated by adding DOX. **B** Gene expression at comparable times of different differentiation protocols, normalized to iNGN2 (NMM-) protocol. The final neurons were replated in NMM+ or NMM-. The dSMADi neurons were only replated in NMM+ according to the original protocol. 5 independent differentiations (different cell lines) were carried out (sBMWi, BMWi, iNGN2; hiPSC_1-5) and for comparison 3 cell lines (hiPSC_7-9) with the dSMADi protocol (results are shown as means ±SEM, same data plotted as Figure 2B). **C** Quantification of IF stainings (hiPSC_1-5) BMWi vs dSMADi neurons 2 and 4 weeks after final plating of the respective protocol (N=5 cell lines BMWi protocol, dSMADi additional hiPSC_8, n=4 technical replicates, results are shown as means ±SEM). **D** Quantification of IF stainings and representative images (hiPSC_1). Neurons were matured 2 weeks after final replating of the respective protocol (N=4 cell lines, hiPSC_1-4, scale bar: 200 µm, insert 3x zoom-in, results are shown as means ±SEM). **E** From day 0 (XAV treatment also from day -1) until day 6, differentiation was performed with and without the addition of dSMADi (LDN and SB) and WNT (IWP2 and XAV) pathway inhibitors, alone and in combination, in order to obtain more cortical neurons on day 13 of iNGN2 neuron differentiation (n=3 independent differentiations from hiPSC_1, except NMM+ and dSMADi n=2, results are shown as means ±SEM). **F** hiPSC_1-4 were differentiated according to protocol adapted from Chen et al. 2020 (dSMADi with following NGN2 overexpression, protocol see supplementary Materials and Methods). qPCR comparison with BMWi protocol shows an increased expression of hindbrain and PNS genes in Chen et al. neurons (N=4 cell lines). Schematic representation of the protocol is also shown. **G** hiPSC were differentiated according to the BMWi protocol. On day 8, half of one differentiation was replated and the other half cryopreserved. The replated NPC were matured for 14 days. The frozen NPC were thawed a week later and matured for 14 days (N=3 cell lines hiPSC_2,3,5, n=4 independent differentiations of each cell line). RNA seq was performed and similarity across replicates from frozen and non-frozen NPC was assessed by Pearson correlation coefficient (r = 0.99, N=3 cell lines, n=4 technical replicate).

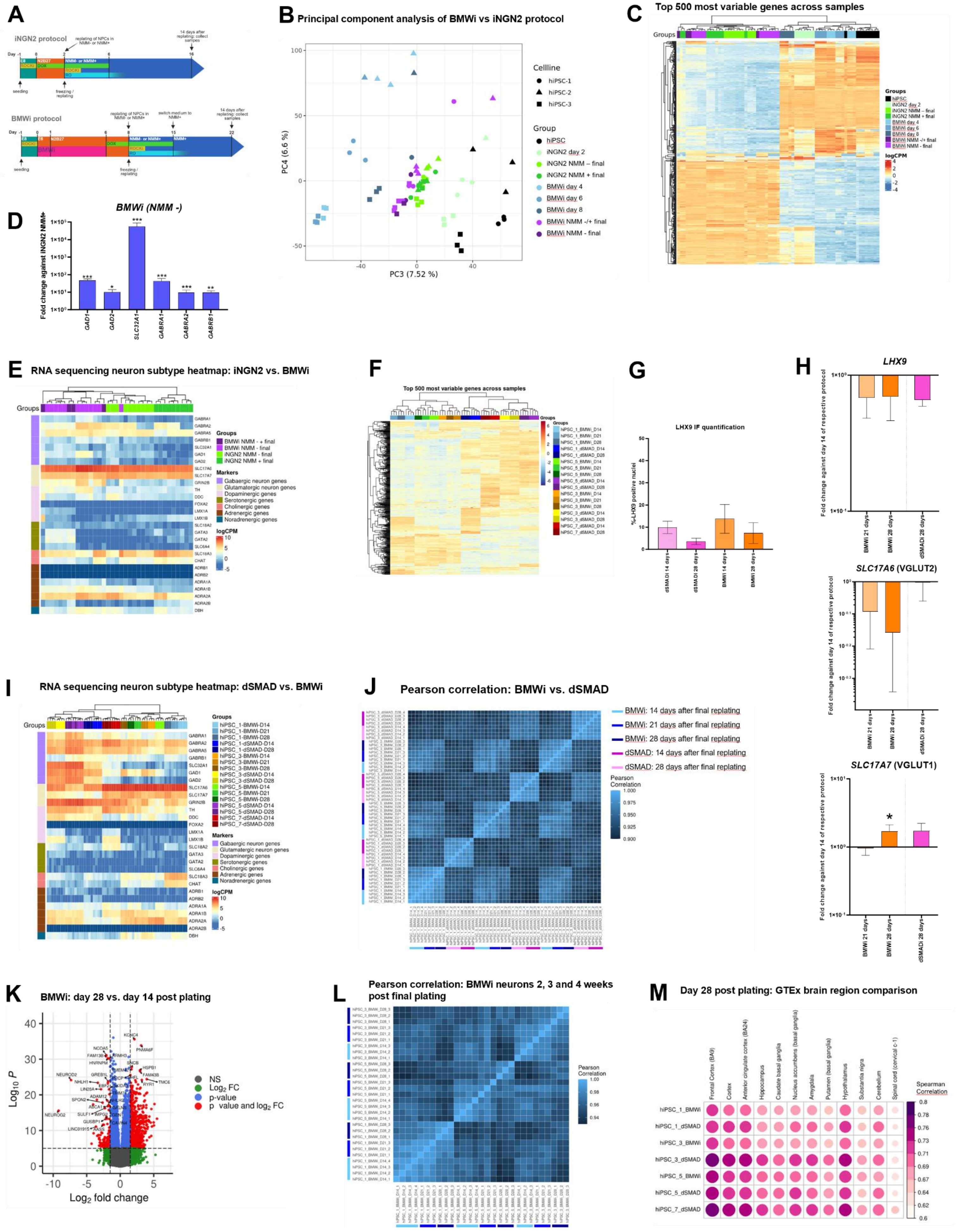

Figure S6: Additional data related to main-text Figure 3. A Schematic BMWi and iNGN2 protocols used for bulk RNA-seq, with respective timepoints of changing NMM media. The neurons were either plated final in NMM + or NMM -. BMWi cells were also replated in NMM- and medium was changed to NMM+ after 7 days (BMWi NMM -/+

final). Sequencing was performed on 3 different cell lines with at least 3 technical replicates (hiPSC_1-3). **B** Principal component analysis of top 500 variable genes shows RNA seq results comparing iNGN2 and BMWi protocol (Figure 3). Shown are PCA dimensions 3 vs. 4 stratifying between different protocols, while first two principal components are not shown as they differentiate cell types. **C** Bulk RNA-seq results showing differences between the iNGN2 and BMWi protocols by hierarchically clustering the 500 most variable genes. **D** Comparison of iNGN2 neurons (NMM+) and BMWi neurons in bulk RNA-seq to show the expression of GABAergic marker genes (results are shown as means ±SEM, * = $p < 0.05$, ** = $p < 0.01$, *** = $p < 0.001$). **E** Heatmap showing hierarchical clustering of BMWi and iNGN2 neurons based on marker genes chosen for different neuronal subtypes (adrenergic, cholinergic, dopaminergic, GABAergic, glutamatergic, noradrenergic and serotonergic genes). Genes not detected across samples were indicated with dark-blue color, at higher intensity than minimum expression levels. **F** Bulk RNA-seq of 2 and 4 weeks matured dSMADi neurons (hiPSC_1,3,5,7, N=4 cell lines, n=3 technical replicates/independent differentiations) compared to 2, 3 and 4 week matured BMWi (hiPSC_1,3,5, N=3 cell lines, n=3 technical replicates/independent differentiations). 500 most variable genes differentiating BMWi and dSMADi. **G** Quantification of IF stainings of LHX9 in 2 and 4 weeks matured dSMADi and BMWi neurons (N=4 cell lines n=2 technical replicates each, results are shown as means ±SEM). **H** Comparison of BMWi and dSMADi bulk RNA-seq timecourse against the respective protocol of gene expression levels during maturation of neurons (N=4 cell lines, hiPSC_1-4, results are shown as means ±SEM, * = $p < 0.05$). **I** Heatmap of bulk transcriptomics at different maturation times of BMWi and dSMADi neurons. Same marker genes are shown as in Figure S6D. Genes not detected across samples were indicated with dark-blue color, at higher intensity than minimum expression levels. **J** Pearson correlation of BMWi and dSMADi bulk RNA-seq replicates. **K** Volcano plot of 4 vs. 2 weeks matured BMWi neurons. **L** Pearson correlation of different maturation timepoints of BMWi neurons (2, 3 and 4 weeks after replating). Legend see I. **M** GTEx brain region analysis of BMWi and dSMADi neurons 28 days after final replating of the respective protocol. Color-intensity and dot size indicate Spearman correlation coefficient.

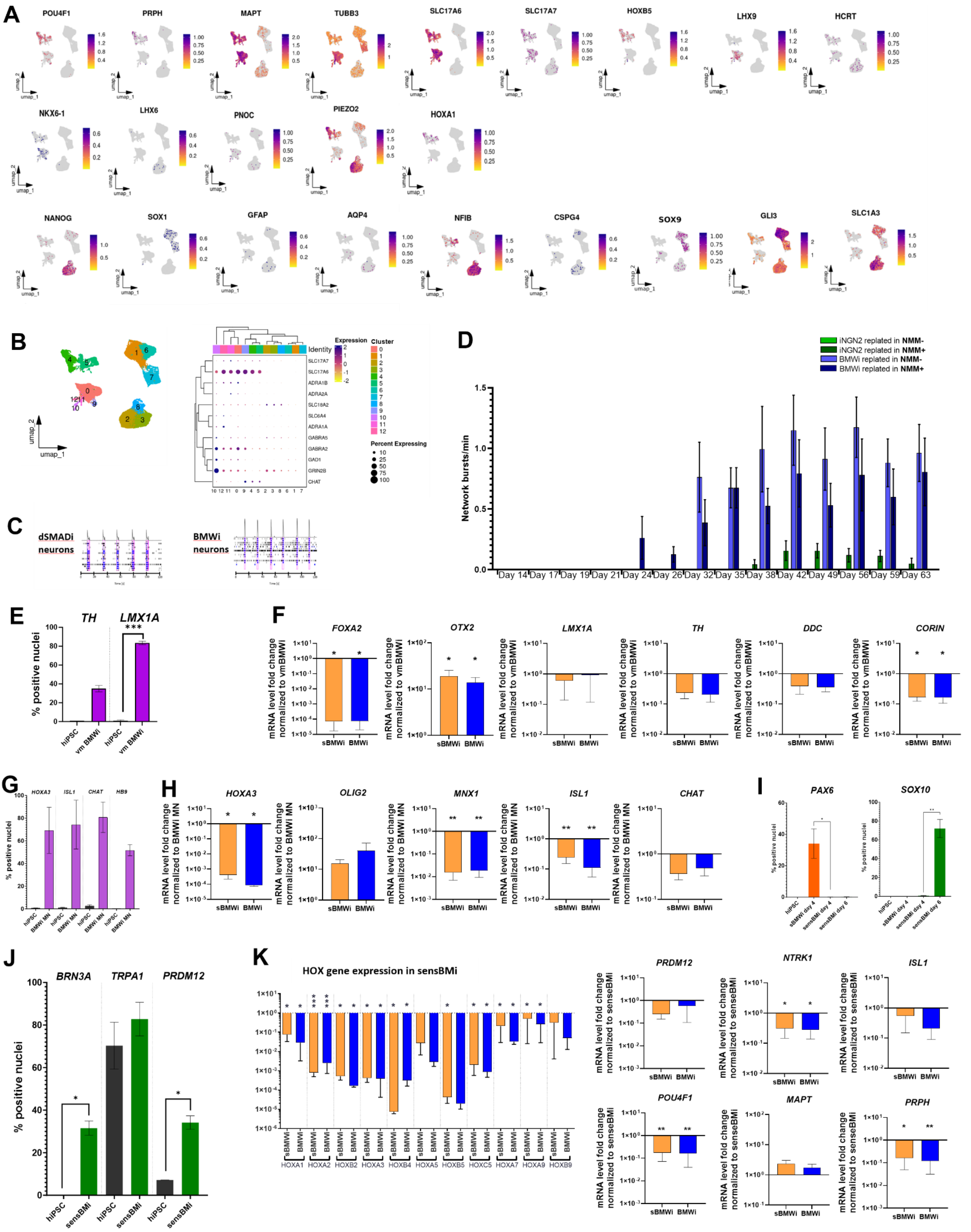

Figure S7: Additional data related to main-text Figures 3/6/7. A Additional analysis of marker genes of snRNA-seq Figure 3E. **B** Clustering of BMWi neurons, BMWi NPC day 6, iNGN2 neurons and hiPSC (hiPSC_3) in snRNA-seq. A bubble plot of marker genes expressed in different clusters is shown. **C** Network activity pattern of MEA measurements of dSMADi neurons and BWMi neurons. 2-minute sections are shown (day 28 after final replating,

hiPSC_1). **D** Determination of the beginning of the electrophysiological activity of the iNGN2 and BMWi neuron cultures. Medium of the neurons replated in NMM- was switched to NMM+ after 7 days. The number of bursts (burst duration min 250 spikes) is plotted against the days after the final replating. Experiment was carried out with 5 different cell lines (hiPSC_1-5) each with 6 technical replicates (results are shown as means ±SEM). The activity of the neurons was recorded for 8 min. **E** Quantification of mDAN marker genes LMX1A and TH of 14 days matured ventral midbrain BMWi (vmBMWi) (Figure 6C, N=4 cell lines, hiPSC_1-4, means ±SEM) **F** Fold change of sBMWi and BMWi neurons 14 days after final replating against vmBMWi 14 days after replating (N=4 cell lines, hiPSC_1-4, all results normalized to vmBMWi of each cell line, means ±SEM). **G** Quantification of marker genes of 15 days matured BMWi MN (Figure 6F, N=4 cell lines, hiPSC_1-4, means ±SEM) **H** Fold change of sBMWi and BMWi neurons 14 days after final replating against BMWi MN 14 days after replating. (N=3 cell lines, hiPSC_1-3, all results normalized to BMWi MN of each cell line, means ±SEM) **I** Quantification of results shown in Figure 7B (N = 2 cell lines, hiPSC_1,5, n=3, n_{hiPSC}=2, means ±SEM). **J** Quantification of data shown in Figure 7C **(**N = 2 cell lines, hiPSC_1,5, n=4, n_{hiPSC}=1, means ±SEM) **K** Fold change of sensBMi neurons normalized to sBMWi and BMWi neurons 14 days after final replating. (* = $p < 0.05$, ** = $p < 0.01$, *** = $p < 0.001$)

Note S1 (Figure S2/3):

It should be noted that the more potent BMP inhibitor LDN provided a stronger BMPi than Dorsomorphin, which is why we chose LDN in this protocol (Figure S2). Incomplete BMPi was assessed by a stronger expression of *TFAP2A* and consistently with the formation of non-neural ectoderm, the use of Dorsomorphin also resulted in non-neuronal, Cytokeratin 18 (CK18) positive cells (not shown). These cells were not observed in conditions with LDN as inhibitor.

Interestingly, a commonly used alternative WNT inhibitor, XAV939 ("XAV"), inhibiting tankyrase, and thus canonical WNT signaling (Figure 1A), did not succeed in preventing the formation of neural crest clusters (SOX10+ cells), whereas addition of canonical WNT activator CHIR99021 (GSK3β inhibitor) with BMWi led to the formation of only few SOX10+ cells (Figure S3A/B/C).

In the dSMADi dependent protocols low cell density leads to a differentiation skewed towards neural crest rather than CNS (Chambers et al., 2009; Münst et al., 2018), implying the use of very high cell densities (Manos et al., 2022). We tested the effect of cell seeding density on the expression of *PAX6, FOXG1 SOX10* and *OCT4*, and found no negative effect of low seeding density (Figure S3D). To compromise between obtaining as many cells as possible from a cell culture vessel and preventing cells from starvation over the course of a weekend, we used the density of 36.000 cells/cm^2 below, unless stated differently.

Note S2 (Figure S4):

We carried out the differentiation described by (Walsh et al., 2020) with the indicated cell lines. In all 6 lines, an increase of *PAX6* and *FOXG1* expression was obtained at the end of the induction period (Walsh et al. 2 days, BMWi 4 or 6 days), but the levels were 7.3-fold lower for *PAX6* and 34.5-fold lower for *FOXG1* (Figure S2A, Figure S4A). It should be noted that Walsh and colleagues used a different inhibitor for FGF2-i, BGJX398 "BGJ" that inhibits the FGF2 receptor rather than the downstream signaling pathways, such as the MAPK/ERK pathway. Another FGF2 dependent signaling pathway, the PLCγ/Ca^{2+} signaling pathway, is associated with the stimulation of neurite growth, but also cell survival. We probed both signaling pathways by measuring the ratio between the unphosphorylated and phosphorylated protein and observed a strong effect of BGJ on both, whereas PD mostly targeted MAPK/ERK, even to an apparently stronger extend than BGJ (Figure S4B/C). We also experienced cell death through inhibition with BGJ or BGJ in combination with BMPi (Figure S4C as an example). That could explain why PD accelerated neural induction without affecting cell survival (Doherty and Walsh, 1996; ZHANG and LIU, 2002).

Note S3 (Figure S5E):

We evaluated XAV alone, added to the culture one day before iNGN2 induction, dSMADi alone, dSMADi with IWP2, IWP2 alone and IWP2 in combination with XAV, together with the iNGN2, replated in NMM- (Figure S5E). We did not observe a strong increase of *FOXG1* or *TBR1* expression on mRNA level at 6 or 13 days of the experiment. The strongest effect on the neuron cultures was achieved with XAV alone, whereas the combination with dSMADi was not beneficial, especially for TBR1 expression. Nevertheless, the increase of *FOXG1* expression in the late timepoint was only 1.5-fold over the iNGN2 alone induction.

Supplementary experimental procedures

hiPSC culture conditions (related to main text materials & methods)

Table S1: *List of cell lines.*

Name	Referred as	Source/ Modification	Disease genotype	Sex	Age	Reprogramming Method	Modification
**BIONi010-C-13**	hiPSC_1 ●	EBiSC*	Control cell line	male	15-19	Episomal Reprogramming factors: KLF4, Lin28, MYC, POU5F1, shP53, SOX2	edited using CRISPR at CRO (Bioneer) with a DOX-inducible NGN2 overexpression construct introduced in the AAVS1 alleles (one allele CAG:m2rtTA, other allele TRE:NGN2)
**BIOMEDi004-A (Coriell Fibroblast AG08125)**	hiPSC_2 ▲	BioMedX/ Bioneer	sAD	male	64	RNA based reprogramming (Schöndorf et al., 2019), Reprogramming factors: OSKM	see hiPSC_1
**iPSC0028 MAPT TM (iNGN2 insert)**	hiPSC_3 ■	EBiSC/ internal edit/ Bioneer	iPSC0028 with MAPT P301S/ E10+14/ E10+16, all biallelic	female	24	see hiPSC_2	edited using CRISPR at CRO with a DOX -inducible NGN2 overexpression construct containing both, CAG:m2rtTA and TRE:NGN2 in one cassette introduced in one AAVS1 allele
**BIOMEDi001-A (Coriell Fibroblast AG08379)**	hiPSC_4 ♦	BioMedX/ Bioneer	Control cell line	female	60	see hiPSC_2	see hiPSC_1
**BIOMEDi005-A (Coriell Fibroblast AG06869)**	hiPSC_5 ★	BioMedX/ Bioneer	sAD	female	60	see hiPSC_2	see hiPSC_1
**SBAD3 Cl1**	hiPSC_6 ⬢	IMI StemBANCC	Control cell line	female	31	Sendai virus, Reprogramming factors: OSKM	-
**iPSC0028 MAPT WT**	hiPSC_7 ▼	Sigma Aldrich	Control cell line	female	24	Retroviral, Reprogramming factors: OSKM	-
**iPSC0028 MAPT TM**	hiPSC_8 □	EBiSC/ internal edit	MAPT P301S/ E10+14/ E10+16, all biallelic	female	24	see hiPSC_7	-
**iPSC0028 MAPT KO**	hiPSC_9 ○	Sigma Aldrich/ internal edit	Knockout of MAPT	female	24	see hiPSC_7	-

Western Blots (related to Figure S4)

Cells were lysed on ice for 10 min with RIPA buffer (ThermoFisher) supplemented with Halt Protease & Phosphatase Inhibitor Cocktail (ThermoFisher). The lysate was then centrifuged and the supernatant taken. Protein concentration was determined via BCA (Pierce BCA Protein Asay Kit, ThermoFisher, 23225) and 15 µg protein loaded onto a gel (Criterion TGX, 4-20%, BIO-RAD, 5671095). The quantitative analyse were performed with Image Lab 6.0.

Table S2: *Primary antibodies for western blot.*

1st antibody	Company	Order number	Dilution
P44/42 MAPK (ERK1/2) (137F5) Rabbit mAb	Cell Signaling	4695	1:950
Phospho-p44/42 (ERK1/2) (Thr202/Tyr204) (D13.14.4E) XP Rabbit mAb	Cell Signaling	4370	1:950
PLCγ1 (D9H10) XP Rabbbit mAb	Cell Signaling	5690	1:950
Phospho-PLCγ1 (Tyr783) (D6M9S) Rabbit mAb	Cell Signaling	14008	1:950
Recombinant Anti-Vinculin antibody [EPR19579]	Abcam	Ab207440	1:10,000

Table S3: *Secondary antibody for western blot.*

2nd antibody	Company	Order number	Concentration	Dilution
Goat Anti-Rabbit IgG H&L (HRP)	Abcam	ab97051	1 mg/mL	1:10,000

Neural induction (related to main text materials & methods)

Table S4: *N2B27 medium.*

Ingredient	Concentration	Source
DMEM/F12	0.5x	ThermoFisher
Neurobasal medium	0.5x	
N2 supplement	0.5x	
B27 supplement (w/o vitamin A)	0.5x	
Penicillin-Streptomycin (5,000 U/mL)	50 U/mL	
GlutaMax	0.5 mM	

Table S5: *Neural maturation medium (NMM).*

Medium	Ingredient	Concentration	Source
**NMM+**	Neurobasal plus medium	1x	ThermoFisher
**NMM+**	B27 Supplement plus supplement	1x	ThermoFisher
**NMM-**	Neurobasal medium	1x	ThermoFisher
**NMM-**	*B27 Supplement w/o vitamin A*	1x	ThermoFisher
**NMM+ and NMM-**	GlutaMax	1 mM	ThermoFisher
	Penicillin-Streptomycin (5,000 U/mL)	50 U/mL	ThermoFisher
	BDNF	10 ng/mL	Biotechne
	GDNF	5 ng/mL	Biotechne
	dcAMP	200 µM	Merck
	L-Ascorbic acid	200 µM	Merck
	Laminin	2 µg/mL	Merck

Immunofluorescence staining (related to all IF stainings in main text)

In this supplementary following antibody was used additionally to the antibodies in main text.

Table S6: *Supplementary antibodies.*

Target	Name	Stock concentration	Dilution	Source	Identifier
**FOXG1**	Anti FOXG1 antibody - ChIP Grade	1 mg/mL	1:150	Abcam	ab18259
**MAP2**	Anti-MAP2 antibody	19 mg/mL	1:10 000	Abcam	ab5392
**Tau aggregates/ MC1**	Anti MC1 antibody	12.4 mg/mL	1:5000	Peter Davies Lab	-
**PAX6**	Pax6, bioreactor supernatant	500 µg/L	1:60 - 1:80	DSHB	AB 528427
**EN1**	4G11, concentrate	466 µg/mL	5 µg/mL	DSHB	AB_2314371
**CHAT**	Anti-Choline Acetyltransferase Antibody	n/a	1:100	Merck	AB144P
**HB9**	81.5C10, bioreactor supernatant	430 µg/mL	1:86	DSHB	AB_2145209
**HOXA3**	HOXA3 Polyclonal Antibody	0.2 mg/mL	1:100	ThermoFisher	PA5-56077
**TH**	Tyrosine Hydroxylase Antibody	n/a	1:5 000	Immunostar	22941
**FOXA2**	Recombinant Anti-FOXA2 antibody [EPR4466]	n/a	1:300	Abcam	ab108422
**ISL1**	40.2D6, concentrate	234 µg/mL	1:47	DSHB	AB_528315
**TRPA1**	TRPA1 Antibody – BSA free	1 mg/mL	1:200	Biotechne	NB110-40763

Target	Name	Stock concentration	Dilution	Source	Identifier
**PRDM12**	PRDM12 Polyclonal Antibody	1 mg/mL	2 µg/mL	ThermoFisher	PA5-60257
**VGLUT2**	VGLUT2 (D7D2H) Rabbit mAb	n/a	1:100	Cellsignaling	71555
**VGLUT1**	VGLUT1 (E9D2B) Mouse mAB	n/a	1:800	Cellsignaling	98199
**Cytokeratin 18**	Anti-Cytokeratin 18 antibody ab52948	1mg/mL	3.3µg/mL	Abcam	ab52948
**PRPH**	Alexa Fluor® 488 Anti-Peripherin antibody [EPR23445-28]	n/a	1:400	Abcam	ab275150
**LHX9**	LHX9 Polyclonal Antibody	1.41 mg/mL	1:150	ThermoFisher	PA5-88722
**HOXA1**	Anti-HOXA1 antibody	n/a	1:100	Abcam	ab168179

Brain region analysis (related to Figure S6)

The data used for the brain region analysis described in this manuscript were obtained from the GTEx Portal on 01/04/2023 as median gene-level TPMs by tissues (v8, RNASeQCv1.1.9) (Lonsdale et al., 2013). For selected brain region specific tissues and spinal chord tissue, Spearman correlation coefficients were assessed between mean expression levels per sample group of BMWi/dSMADi protocol data and GTEx expression. The R package corrplot (v0.92) (Wei and Simko, 2021) was used to visualize results with circle size and color intensity encoding correlation coefficients.

Alternative coating method with Laminin (related to Figure S3)

Cell culture dishes were coated with 5 µg/mL LN521 (BioLamina) diluted in DPBS (+/+). 80 µL coating solution was added to each 96 well and 2 mL to each 6 well. Dishes were sealed with parafilm and stored at 4°C on a shaker for a minimum of 2 hours. The dishes can be stored in the fridge up to 3 weeks. After one week in the fridge, the coating solution needed to be replenished with DPBS (+/+) to prevent drying during long-term storage. This method was used in Figure S3A-C, E.

Reproduction of Walsh *et al*. (related to Figure S5)

All experiments were carried out on vitronectin-coated plates. IPSCs were cultured for 4 days until they were confluent and passaged with hypertonic citrate buffer and replated in E8

medium + 10 µM ROCKi. 24 h after passage, the medium was changed to E6 supplemented with 500 nM LDN and 100 nM BGJ. 24 h later a complete medium change was carried out with E6 medium supplemented with 500 nM wntC59, 20 ng/mL FGF2 and 500 nM A8301. The final samples were taken 24 h after the last medium change.

Reproduction of Nehme *et al*. (related to Figure S5)

100,000 cells/well were plated on Matrigel coated 6 well plates in E8 flex medium + 10 µM ROCKi. On day 0 medium was changed to N2B27, 2 µg/mL DOX, 100 nM LDN, 10 µM SB and 2 µM XAV. Day 1 a complete medium change with N2B27, 2 µg/mL DOX, 100 nM LDN, 10 µM SB and 2 µM XAV was performed. After 3 days, the cells were replated as described in main text materials and methods and replated in NMM-, DOX, LDN, SB and XAV, 10 µM ROCKi and 500 nM RO. The day after, 90% of the medium was replaced to NMM-, 2 µg/mL DOX, LDN, SB and XAV, and RO to remove ROCKi. On day 6, medium was replaced with NMM+ medium. The medium was 50% replaced every 4-5 days.

Reproduction of Chen *et al*. (related to Figure S5)

250.000 cells were plated in each 6 well in E8flex medium + 10 µM ROCKi. Day 0 medium was changed to N2B27 supplemented with 100 nM LDN + 10 µM SB. A complete medium change was performed each day until day 6. Day 6 medium was changed to N2B27 + 2 µg/mL DOX. After 5 days the cells were replated as in main text materials and methods described in NMM- + DOX + 2.5 µM DAPT (Tocris) + ROCKi. After 2 days DOX was removed from the medium. The medium was 50% replaced every 4-5 days. 14 days after final replating the neuons were lysed.

Quantitative real-time PCR (related to all PCR experiments of this manuscript)

Table S7: Primer real-time PCR.

Gene	Company	Assay ID
CHAT	Thermo Fisher	Hs00758143_m1
CORIN	Thermo Fisher	Hs00198141_m1
DCC	Thermo Fisher	Hs00180437_m1
FOXA2	Thermo Fisher	Hs00936490_m1
FOXG1	Thermo Fisher	Hs01850784_s1
GAPDH	Thermo Fisher	Hs99999905_m1
HOXA1	Thermo Fisher	Hs00939046_m1
HOXA2	Thermo Fisher	Hs00534579_m1
HOXA3	Thermo Fisher	Hs00601076_m1
HOXA7	Thermo Fisher	Hs00600844_m1
HOXA9	Thermo Fisher	Hs04931836_m1
HOXB2	Thermo Fisher	Hs01911167_s1

Gene	Company	Assay ID
HOXB4	Thermo Fisher	Hs00256884_m1
HOXB5	Thermo Fisher	Hs00357820_m1
HOXB9	Thermo Fisher	Hs00256886_m1
HOXC5	Thermo Fisher	Hs00232747_m1
IRX3	Thermo Fisher	Hs01124217_g1
ISL1	Thermo Fisher	Hs00158126_m1
LMX1A	Thermo Fisher	Hs00892663_m1
MAPT	Thermo Fisher	Hs00902192_m1
MNX1	Thermo Fisher	Hs00907365_m1
NANOG	Thermo Fisher	Hs04399610_g1
NKX2.1	Thermo Fisher	Hs00968940_m1
NTRK1	Thermo Fisher	Hs01021011_m1
OLIG2	Thermo Fisher	Hs00300164_s1
OTX2	Thermo Fisher	Hs00222238_m1
PAX3	Thermo Fisher	Hs00992437_m1
PAX6	Thermo Fisher	Hs01088114_m1
POU4F1	Thermo Fisher	Hs00366711_m1
POU5F1	Thermo Fisher	Hs04260367_gH
PPIA	Thermo Fisher	Hs04194521_s1
PRPH	Thermo Fisher	Hs00986945_g1
PRDM12	Thermo Fisher	Hs00964106_m1
RPL13	Thermo Fisher	Hs00744303_s1
SOX1	Thermo Fisher	Hs01057642_s1
SOX10	Thermo Fisher	Hs00366918_m1
TBR1	Thermo Fisher	Hs00232429_m1
TFAP2A	Thermo Fisher	Hs01029413_m1
TH	Thermo Fisher	Hs00165941_m1

Single nuclei RNA sequencing (related to Figure 3 and S7)

Table S8: Lysis buffer for snRNA seq.

Ingredient	Concentration	Source
**NP40 (25%)**	0.1%	Thermo Fisher
**NaCl (1 M)**	10 mM	Merck
**$MgCl_2$ (1 M)**	3 mM	Merck
**Tris-HCl (pH 7.4, 1 M)**	10 mM	Merck
**DPBS (-/-)**	-	Thermo fisher

Table S9: Washing buffer for snRNA seq.

Ingredient	Concentration	Source
35% BSA solution	1%	Merck
Protector RNase inhibitor	0.2 U/µL	Roche
DPBS (-/-)	-	Thermo Fisher

Supplementary references:

Chambers, S.M., Fasano, C.A., Papapetrou, E.P., Tomishima, M., Sadelain, M., and Studer, L. (2009). Highly efficient neural conversion of human ES and iPS cells by dual inhibition of SMAD signaling. Nat Biotechnol *27*, 275–280. https://doi.org/10.1038/nbt.1529.

Doherty, P., and Walsh, F.S. (1996). CAM-FGF Receptor Interactions: A Model for Axonal Growth. Mol Cell Neurosci *8*, 99–111. https://doi.org/10.1006/mcne.1996.0049.

Lonsdale, J., Thomas, J., Salvatore, M., Phillips, R., Lo, E., Shad, S., Hasz, R., Walters, G., Garcia, F., Young, N., et al. (2013). The Genotype-Tissue Expression (GTEx) project. Nat. Genet. *45*, 580–585. https://doi.org/10.1038/ng.2653.

Manos, J.D., Preiss, C.N., Venkat, N., Tamm, J., Reinhardt, P., Kwon, T., Wu, J., Winter, A.D., Jahn, T.R., Yanamandra, K., et al. (2022). Uncovering specificity of endogenous TAU aggregation in a human iPSC-neuron TAU seeding model. Iscience *25*, 103658. https://doi.org/10.1016/j.isci.2021.103658.

Münst, S., Koch, P., Kesavan, J., Alexander-Mays, M., Münst, B., Blaess, S., and Brüstle, O. (2018). In vitro segregation and isolation of human pluripotent stem cell-derived neural crest cells. Methods *133*, 65–80. https://doi.org/10.1016/j.ymeth.2017.09.012.

Walsh, P., Truong, V., Nayak, S., Montivero, M.S., Low, W.C., Parr, A.M., and Dutton, J.R. (2020). Accelerated differentiation of human pluripotent stem cells into neural lineages via an early intermediate ectoderm population. Stem Cells *38*, 1400–1408. https://doi.org/10.1002/stem.3260.

Wei, T., and Simko, V. (2021). R package “corrplot”: Visualization of a Correlation Matrix (Version 0.92). https://github.com/taiyun/corrplot.

ZHANG, W., and LIU, H.T. (2002). MAPK signal pathways in the regulation of cell proliferation in mammalian cells. Cell Res *12*, 9–18. https://doi.org/10.1038/sj.cr.7290105.
