# Supplementary material for: BMP, MEK, and WNT inhibition with NGN2 expression for rapid generation of hiPSC-derived neurons amenable to regional patterning
Source: Stem Cell Reports. 2025 Jun 19;20(7):102539. doi: 10.1016/j.stemcr.2025.102539 (PMC12277818; doi:10.1016/j.stemcr.2025.102539)
Supplement: Document S2. Article plus supplemental information [file mmc2.pdf]

Stem Cell Reports
Resource
OPEN ACCESS

BMP, MEK, and WNT inhibition with NGN2 expression for rapid generation of hiPSC-derived neurons amenable to regional patterning

Carina Habich,[1,9] Alexandra Kowalski,[2,9] Astrid Wachter,[3] Michaela J. Heimann,[1] Michael Wolf,[1] Markus P. Kummer,[1] Nathalie Nicolaisen,[1] Christopher Sliwinski,[1] Lydia Reinhardt,[1] Veronika Heil,[1] Timo Lange,[3] Christopher Untucht,[1] Loan N. Miller,[4] Jürgen Korffmann,[1] Daniela Geist,[1] David Schöndorf,[1] Heyne Lee,[1] Lamiaa Bahnassawy,[1] Benjamin Mielich-Süss,[1] Melanie S. Brennan,[5] Ruven Wilkens,[1] Julian Röwe,[1] Ian Weidling,[5] Rüdiger Rudolf,[6,7] Mathias Hafner,[6,8] Justine D. Manos,[5] Miroslav Cik,[1] and Peter Reinhardt[1,10,*]

[1]AbbVie Deutschland GmbH & Co. KG, Neuroscience Discovery, Knollstrasse, 67061 Ludwigshafen, Germany
[2]Institute of Molecular and Cell Biology, Mannheim University of Applied Sciences, 68163 Mannheim, Germany
[3]AbbVie Deutschland GmbH & Co. KG, Genomics Research Center, Knollstrasse, 67061 Ludwigshafen, Germany
[4]AbbVie Inc., Pharmacology and Pathology, Lake County, IL 60064, USA
[5]AbbVie Inc., Cambridge Research Center, 200 Sidney Street, Cambridge, MA 02139, USA
[6]Institute of Molecular and Cell Biology, Mannheim, University of Applied Sciences, D-68163 Mannheim, Germany, and Interdisciplinary Center for Neurosciences, Heidelberg, University, 69117 Heidelberg, Germany
[7]Center for Mass Spectrometry and Optical Spectroscopy, Mannheim University of Applied Sciences, 68163 Mannheim, Germany
[8]Institute of Medical Technology, Heidelberg University and Mannheim University of Applied Sciences, 69117 Heidelberg, Germany
[9]These authors contributed equally
[10]Lead contact
*Correspondence: peter.reinhardt@abbvie.com
https://doi.org/10.1016/j.stemcr.2025.102539

SUMMARY

Human induced pluripotent stem cells (hiPSCs) are a promising tool for studying neurological diseases and developing therapies for neurodegenerative diseases. Differentiation of hiPSCs into neurons can be achieved by dual SMAD inhibition (dSMADi) or by induced neurogenin 2 (NGN2) overexpression ("iNGN2"). Starting directly from hiPSCs, iNGN2 shortens the time to a neuronal stage but leads to neurons partially resembling peripheral or posterior fates while dSMADi more faithfully recapitulates telencephalic development. To modify the iNGN2 approach, we applied an accelerated induction paradigm that is dependent on the inhibition of BMP, MEK, and WNT pathways ("BMWi"), to commit hiPSCs into a telencephalic fate before iNGN2. The resulting neurons showed strong expression of telencephalic markers, with decreased levels of peripheral and posterior marker genes compared to iNGN2 alone. The resulting telencephalic neurons are suitable for a tau aggregation assay. Furthermore, we could demonstrate that during BMWi treatment, the cells are amenable to additional regional patterning cues. This allowed the generation of neurons from different regions of the CNS and peripheral nervous system (PNS), which will significantly facilitate *in vitro* modeling of a range of neurodevelopmental and neurodegenerative disorders.

INTRODUCTION

Access to neurons of the central nervous system (CNS) has been a limiting factor in the research of neurodegenerative diseases, such as Alzheimer's disease (AD), Parkinson's disease, and amyotrophic lateral sclerosis. With the human induced pluripotent stem cell (hiPSC) technology, *bona fide* human neurons have been generated for the first time at scale from a pluripotent stem cell (PSC) type that is not affected by major ethical concerns, such as human embryonic stem cells (Sterneckert et al., 2014). In disease, often specific neuronal subtypes are affected stronger or earlier and thus desired for *in vitro* modeling. In AD, glutamatergic excitatory neurons located in the frontal cortex are severely affected (Campos-Peña and Meraz-Ríos, 2014; Liu et al., 2019). One of the hallmarks of AD is the formation of neurofibrillary tangles of tau, which were also modeled in this study.

In human PSC, the undifferentiated state is maintained by two major signaling pathways: FGF2 (basic FGF [fibroblast growth factor]) signaling through the MEK (MAPK/ERK kinase)/ERK (extracellular signal-regulated kinase) pathway, and activation of the transforming growth factor β (TGF-β)/Activin/Nodal pathway through the SMAD2/3 cascade (Greber et al., 2011). As in embryonic development, during which inhibition of SMAD2/3 signaling in ectoderm is modulated by factors secreted by the underlying mesoderm, inhibition of SMAD2/3 facilitates neuroectoderm formation (Smith et al., 2008). BMP (bone morphogenic protein) signaling mediated by SMAD1/5/8 triggers trophectoderm formation and thereby prevents neuroectodermal fates and will later lead to non-neural ectoderm. Thus, inhibiting the BMP pathway also facilitates neuroectoderm formation (Pera et al., 2004). Inhibition of both SMAD signaling pathways (dual SMAD inhibition [dSMADi] (Chambers et al., 2009)) has therefore become a widely used strategy to guide human PSCs to a neuroectodermal fate. Conveniently, dSMADi can be combined with regional patterning cues, such as inhibition of WNT

© 2025 The Author(s).
Published by Elsevier Inc. on behalf of International Society for Stem Cell Research.
This is an open access article under the CC BY license (http://creativecommons.org/licenses/by/4.0/).

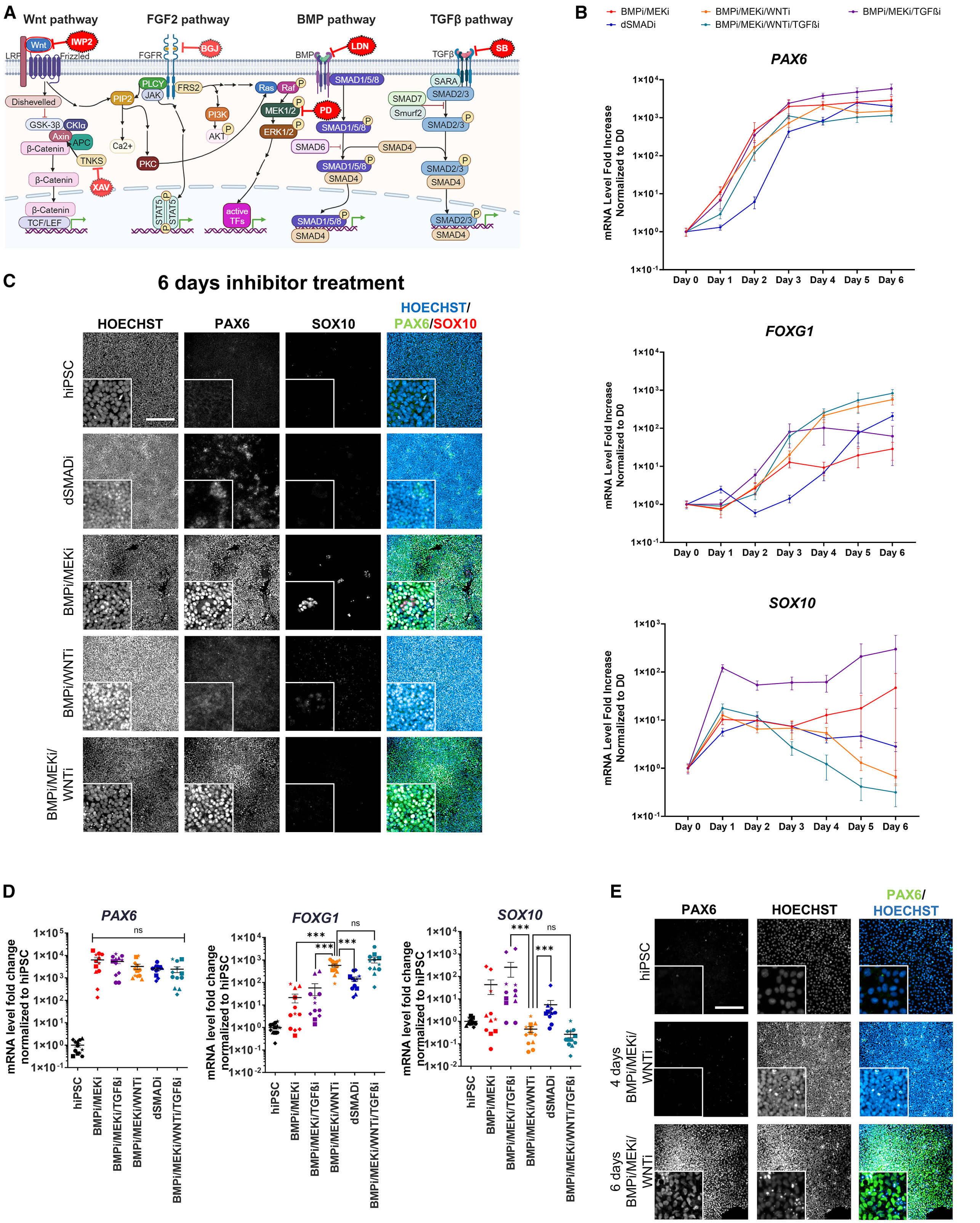

(legend on next page)

signaling to promote anterior regionalization (Chen et al., 2020; Rao et al., 2016), ventralization by SHH (sonic hedehog) signaling (Shi et al., 2012), posteriorization by retinoic acid signaling (RA) such as for motor neurons (Calder et al., 2015), and others (Tao and Zhang, 2016).

While these paradigms use signaling modulation, a different approach relies on the controlled overexpression of neurogenic transcription factors, in particular neurogenin 2 (NGN2, in the doxycycline (DOX)-inducible variant called "iNGN2") (Zhang et al., 2013). Initial reports indicated that the resulting cells exhibit a telencephalic identity (e.g., CUX1/2 expression (Wang et al., 2017)). However, expression of key telencephalic genes, such as *FOXG1*, was often not evaluated, or their expression was absent (Wang et al., 2017). Similarly, other telencephalic layer markers expressed by hiPSC-derived excitatory telencephalic neurons, such as *TBR1*, labeling glutamatergic excitatory neurons in telencephalic layers V/VI, were absent (Fiock et al., 2020; Hof et al., 1991). Later studies had indicated a mixed identity of CNS and PNS (peripheral nervous system) neurons in iNGN2 neurons (Chen et al., 2020; Lin et al., 2021). Similar to these studies, we confirmed that neurons differentiated with iNGN2 express PNS/hindbrain marker genes. For this reason, we explored a rapid telencephalic neuroectoderm induction paradigm to be included before the iNGN2 activation. We identified culture conditions that possess a powerful posteriorizing effect on iNGN2-derived neurons. We explored strategies to induce telencephalic neuroectoderm prior to iNGN2 in multiple hiPSC lines to establish a protocol that is comparable in time to iNGN2 but results in neurons with a robust telencephalic identity. hiPSCs could be differentiated into PAX6-expressing neuroectoderm (Zhang et al., 2010) with BMP/MEK/WNT inhibition (BMWi) in as short as 4–6 days. Followed by iNGN2, these cells rapidly turned into a much stronger telencephalic identity that can be used for disease modeling as shown by tau aggregation. Incorporating additional regional cues allowed modulation of identity to ventral, midbrain floorplate, motor neuron (MN), and dorsal-root-ganglia-like fates. In summary, this SMAD2/3 inhibition-independent protocol is rapid and universally applicable to multiple hiPSC donor lines at a wide range of cell densities and can be readily combined with additional patterning cues to derive a variety of neuronal subtypes. Activation instead of inhibition of WNT signaling allows even differentiation of functional sensory neurons.

RESULTS

Accelerated induction of neuroectodermal progenitor cells independent of SMAD2/3 inhibition

For accelerated induction of neuroectoderm progenitor cells from hiPSC, we combined dSMADi (LDN193189 "LDN/BMPi", a BMP signaling inhibitor and SB431542 "SB/TGF-βi", a TGF-β/Activin signaling inhibitor) with inhibition of the FGF2 (specifically the MEK/ERK) signaling cascade using the MEK inhibitor PD0325901 ("PD/MEKi") (Greber et al., 2011). In combination with dSMADi, or LDN alone, PD was applied to 6 different hiPSC lines (Figure 1A; Table S1). In agreement with Greber et al. (2011), the BMPi/TGF-βi/MEKi accelerated neural induction compared to dSMADi, assessed by the expression of the neuroectodermal fate marker *PAX6* (Figure 1B). Interestingly, no strong difference of *PAX6* expression was observed upon the omission of TGF-βi. However, in BMPi/TGF-βi/MEKi, and BMPi/MEKi, the expression of the neural crest marker *SOX10* increased (Figure 1B). In 3 of 6 cell lines with BMPi/MEKi, neural crest tissue, determined by the expression of SOX10 (Figure 1C; quantification $N = 5$ cell lines Figure S1A), could also be observed. During development, the formation of neural crest is dependent on WNT signaling. Additional inhibition of the WNT signaling pathway (WNTi) by the porcupine inhibitor IWP2 prevented neural crest specification without affecting the rate of neural induction, as measured by *PAX6* expression (Figure 1B). To assess the generation of telencephalic cells, we measured the expression of *FOXG1*, which was accelerated by MEKi and increased by added WNTi (Figure 1B). Further TGF-βi showed no significant increase of *FOXG1* expression (Figure 1D shows day 6; day 1–5 are shown in detail in Figure S1B). *FOXG1* expression was also significantly higher in BMWi compared to dSMADi. The expression of the neural crest marker *SOX10* was, as expected, highest in TGF-βi/BMPi/MEKi and also significantly increased after 6 days of dSMADi

Figure 1. Identifying an induction paradigm for neuroectoderm

(A) Schematic of the signaling pathways and their inhibitors (red) used in this study.

(B) Time course of gene expression measured by real-time qPCR after treatment of hiPSCs (hiPSC_1–6) with different combinations of inhibitors from day 0 to day 6 (results are shown as means ± SEM; $N = 6$ different cell lines, see also Figures S1B and S2).

(C) Representative IF staining (hiPSC_5) after 6 days of treatment.

(D) Gene expression after 6 days of treatment with the inhibitor combination indicated. 6 different cell lines (hiPSC_1–6) were used, and a total of 12 independent differentiations were performed ($N = 6$ cell lines, each with $n = 2$ independent differentiations).

(E) IF staining of hiPSC_5 after treatment with BMWi for 4 and 6 days (scale bars: 200 μm, insert 3× zoom-in, $^{*}p < 0.05$, $^{**}p < 0.01$, $^{***}p < 0.001$; ● = hiPSC_1, ▲ = hiPSC_2, ■ = hiPSC_3, ◆ = hiPSC_4, ★ = hiPSC_5, ⬢ = hiPSC_6).

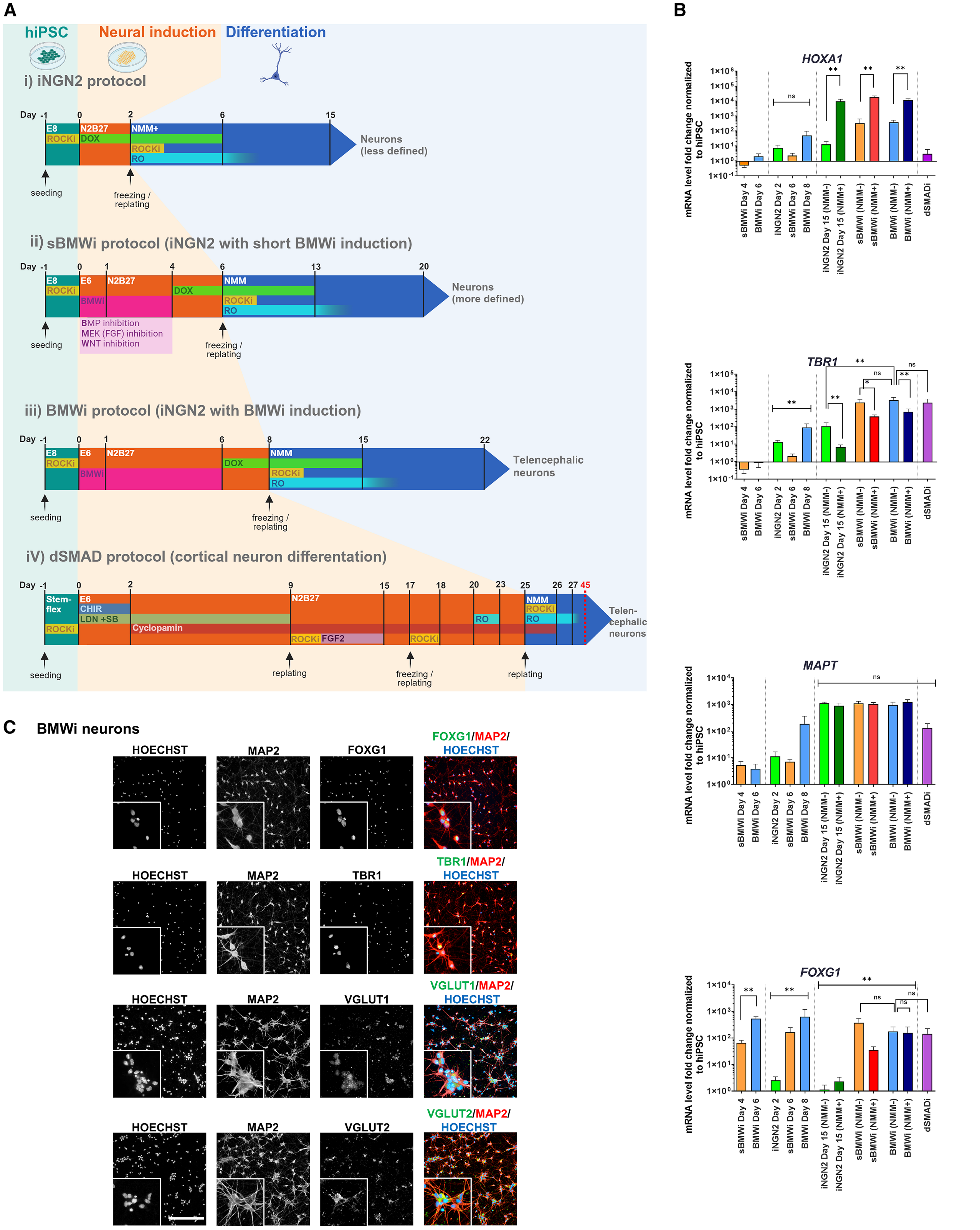

(legend on next page)

compared to BMPi/MEKi/WNTi. Additional TGF-βi did not lead to a significant decrease of *SOX10* expression. Only a low expression of PAX6 could be detected by immunofluorescence (IF) after 6 days of dSMADi treatment (Figure 1C; quantification Figure S1A). BMPi/MEKi/WNTi and BMPi/MEKi showed high PAX6 expression. On the mRNA level, *PAX6* expression reached a plateau after 4 days, whereas robust protein expression was detected after 6 days of BMPi/MEKi/WNTi (Figure 1E; quantification Figure S1C).

We evaluated different combinations of signaling inhibitors in 6 hiPSC lines and assessed mRNA expression of the key marker genes on day 6 of differentiation (Figure S2A): *FOXG1*, *PAX6*, *SOX10*, and *TFAP2A*. MEKi with insufficient BMPi may lead to non-neural ectoderm (Tchieu et al., 2017), which is shown by an increased expression of *TFAP2A*. As expected, BMPi with MEKi led to a slight increase in *TFAP2A*, albeit at a very low expression level (compared to MEKi conditions without BMPi). No $AP2a^+$ cells were detected by IF analysis (Figure S2B).

When comparing BMP inhibitors, LDN was superior to dorsomorphin ("DM") in blocking non-neural ectoderm formation (Figures S2B and S2C). Inhibition with PD/DM showed stronger *TFAP2A* expression and $TFAP2A^+$ cells via IF compared with the inhibition with PD/LDN (Figure S2B). WNT inhibitor XAV939 was not sufficient to block neural crest formation (Figures S3A–S3C). The neural induction was largely independent of cell density, unlike dSMADi (Figure S3D, Note S1). No formation of neural rosettes was observed in BMWi conditions but could be induced by FGF2 following the BMWi treatment (Figure S3E).

Walsh et al. (2020) had previously published a different two-day protocol including FGF2i, together with dSMADi and WNTi, but in separate steps (BMPi followed by TGF-βi). We compared their protocol with the BMWi induction paradigm and observed lower levels of *PAX6/FOXG1* and significant cell loss (Figures S4A, S4C, and S2A, Note S2; Tables S2 and S3). It should be noted that Walsh and colleagues used a different inhibitor for FGF2i, BGJX398 ("BGJ"). This inhibits the FGF2 receptor rather than the downstream signaling pathways, such as the mitogen-activated protein kinase (MAPK)/ERK pathway (see also Figure 1A). Another FGF2-dependent signaling pathway, the PLCγ/Ca^{2+} signaling pathway, is associated with the stimulation of neurite growth but also with cell survival. We probed both signaling pathways by measuring the ratio between the unphosphorylated and phosphorylated protein by western blot and observed a strong effect of BGJ on both, whereas PD mostly targeted MAPK/ERK, even to an apparently stronger extent than BGJ. That could explain why PD accelerated neural induction without affecting cell survival (Figures S4B and S4C).

In summary, BMWi was sufficient to differentiate hiPSCs into telencephalic, neuroectodermal cells, independent of the seeding density, and led to a consistent outcome across different hiPSC lines.

Combination of BMWi and NGN2 overexpression differentiates hiPSCs into telencephalic neurons

To determine whether BMWi could be applied prior to iNGN2 to direct the fate to telencephalon, we compared four protocols (Figure 2A): a protocol based on quantitative reverse transcription PCR (RT-qPCR) results with 4 days of BMWi and a subsequent 2-day iNGN2 before replating (short BMWi protocol, sBMWi), and a protocol based on the IF results that includes a 6-day BMWi pre-differentiation period and 2-day iNGN2 before replating (BMWi protocol). All neurons were treated with mitomycin C 5 days after final replating to remove single remaining proliferating cells for a pure neuron culture stable for several weeks (Hiller et al., 2021; Manos et al., 2022; Young et al., 2014). We compared these protocols with the iNGN2 protocol (Manos et al., 2022; Stolzenburg et al., 2023) and an established protocol for differentiating dorsal excitatory neurons via dSMADi (Manos et al., 2022). For all differentiations with NGN2 overexpression, we used hiPSCs with integration of an iNGN2 cassette (Figure S5A). Two different media were assessed for replating: neuronal maturation medium (NMM) based on the Neurobasal Plus and B27+ system (NMM+, as used in the study by Manos et al.), and a medium based on Neurobasal and B27 supplement without retinoids (NMM-). Compared to the dSMADi neurons, BMWi and iNGN2 neurons are exposed to NMM

Figure 2. Combined BMWi and iNGN2 accelerate differentiation of hiPSCs into telencephalic neurons

(A) Differentiation protocols used. Abbreviations: RO: RO4929097 γ-secretase inhibitor; DOX, doxycycline, induces NGN2 overexpression; ROCKi, Y-27632 ROCK inhibitor; dSMADi (LDN193189 + SB431542): inhibition of ALK2/3 and ALK4/5/7, dual SMAD inhibition; CHIR, CHIR99021 GSK3 inhibitor/WNT activator. Medium compositions: see Methods.

(B) Gene expression at comparable times of different differentiation protocols. Neurons were replated in NMM+ or NMM-. dSMADi neurons were used as a telencephalic standard and prepared according to the original protocol. 5 independent differentiations (different cell lines) were carried out (sBMWi, BMWi, iNGN2; hiPSC_1–5) and as reference 3 cell lines (hiPSC_7–9) with the dSMADi protocol (means ± SEM). Expression levels were normalized to the average expression of undifferentiated hiPSCs.

(C) Representative IF images of mature neuron cultures (hiPSC_5) of BMWi neurons day 14 after replating (scale bars: 200 μm, insert 3× zoom-in) (*$p < 0.05$, **$p < 0.01$, ***$p < 0.001$).

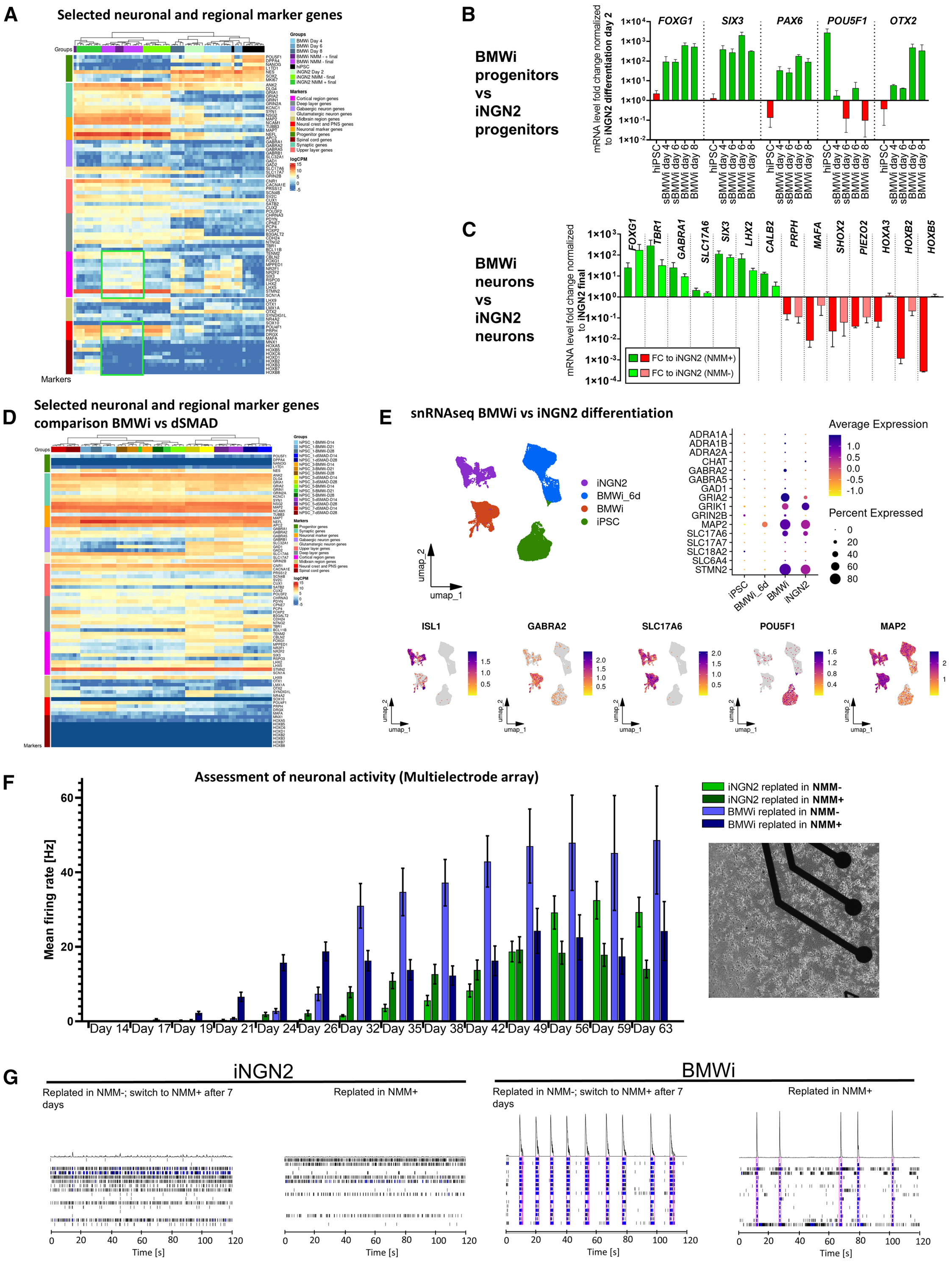

Figure 3. BMWi neurons show telencephalic transcriptome and electrophysiological activity profiles
(A) RNA-seq results showing the comparison of iNGN2 and BMWi protocols. The neurons were either plated in NMM+ or NMM-. BMWi cells were also replated in NMM-, and the medium was changed to NMM+ after 7 days (BMWi NMM-/+ final). The sequencing was carried out with 3 different cell lines with at least 3 technical replicates (hiPSC_1–3). Heatmap showing the expression levels of selected genes, color-

(legend continued on next page)

very early in their development and might be susceptible to patterning factors in the undisclosed medium compositions. Exposure at replating to NMM+ in the BMWi and iNGN2 protocols led to strong expression of posterior genes, such as *HOXA1* (Figure 2B). This indicated the presence of a posteriorizing factor, such as RA (Durston et al., 1989) in NMM+. Initial replating in NMM- was therefore included in the BMWi protocols, unless otherwise stated. Maturation in NMM+ also had significant negative effects on the expression of *TBR1* at the mRNA level (Figures 2B and S5B). In contrast, the expression of *TBR1* and *MAPT* was equivalent in BMWi neurons cultured in NMM- and dSMADi neuron samples 2 weeks post final plating.

For the telencephalic marker *FOXG1*, a positive effect of the BMWi protocols could be observed not only in progenitor cells but also in neurons 2 weeks after replating, as it showed a significant, more than 100-fold increase in gene expression compared to the iNGN2 NMM- protocol (Figure 2B). IF staining revealed FOXG1$^+$ (78.0% ± 12.8% N = 5 cell lines) and TBR1$^+$ (81.5% ± 5.4% N = 5 cell lines) BMWi neurons (Figures 2C and S5C). In line with an excitatory phenotype, the culture is VGLUT1 and VGLUT2 positive (Figure 2C). In comparison to BMWi neurons, iNGN2 neurons are more PRPH$^+$ (58.7% ± 17.6% N = 4 cell lines) and HOXA1$^+$ (15.4% ± 12.9% N = 4 cell lines) (Figure S5D).

Nehme et al. (2018) published an approach to obtain a more telencephalic-like gene expression pattern by combining dSMADi with XAV939 and iNGN2 in parallel. Under these conditions, we observed only mildly increased levels of *FOXG1* and *TBR1* expression compared with iNGN2 alone (Figure S5E, Note S3). To compare the BMWi protocol to a similar protocol with dSMADi and NGN2 overexpression, neurons were differentiated according to Chen et al. (2020), with slight variation. Using dSMADi induction for 6 days followed by 6 days of iNGN2, their conditions were used for induction. Neurons were replated in NMM-. The resulting neurons show a similar expression of *TBR1* and reduced *FOXG1* expression, but *PRPH* and *HOXA1* are significantly increased compared to the BMWi neurons (Figure S5F).

We could further confirm that BMWi neural progenitor cells (NPCs) can be frozen on day 8 of the protocol (replating). Upon thawing, BMWi NPCs had a viability of 91.9% ± 1.8% (N = 3 cell lines hiPSC_1–3, n = 2 technical replicates). We observed no differences in global gene expression between differentiated neurons from fresh versus frozen NPCs, as assessed by RNA sequencing (RNA-seq) (Figure S5G), with a mean Pearson correlation coefficient of 0.99 (N = 3 cell lines, n = 4 individual cultures).

BMWi neuron cultures show an increased expression of telencephalic markers and exhibit the activity of a mature network

To determine the telencephalic identity of the BMWi neurons, we conducted an RNA-seq study to assess whole genome expression. This was performed on hiPSC lines derived from three donors, each of which underwent 3 independent differentiations. The experiment was performed using iNGN2 NMM$^{+/-}$ and BMWi NMM$^{+/-}$ conditions, with sampling at the hiPSC stage, at the end of the BMWi treatment, after the 2-day DOX induction period and 2 weeks after final replating/maturation (schematic protocol: see Figure S6A). Transcriptome analysis showed that neuron cultures clustered separately from neural progenitor stages and from hiPSCs (Figures 3A, S6B, and S6C). Based on 83 selected markers for hiPSCs, NPCs, neurons, synapses, glutamatergic neurons, GABAergic neurons, and regional markers (cortex, deep layer, upper layer,

coded by different regions/types of interest. Hierarchical clustering shows a clear separation of the different time periods of the protocols. Genes showing differential expression between iNGN2 and BMWi protocols are indicated in green boxes (see also Figure S6A schematic protocol, S6B PCA, S6C heatmap 500 most variable genes, and S6E heatmap subtype clustering).
(B) Fold changes (FCs) between NPCs at the replating step. Selected marker genes are shown.
(C) Differential gene analysis of iNGN2 neurons (NMM+ and NMM-) vs. BMWi neurons (NMM-). Selected marker genes are shown.
(D) RNA-seq comparing BMWi and dSMAD neurons at different time points of maturation. Samples were taken 2, 3, and 4 weeks after final replating of the respective protocol (dSMAD samples 2 and 4 weeks). Sequencing was performed on 3 different cell lines of BMWi neurons (hiPSC_1,3,5) and 4 different cell lines of dSMAD neurons (hiPSC_1,3,5,7) (all conditions with 3 technical replicates). Heatmap showing the expression levels of selected genes, color-coded by different brain regions/marker types of interest, as shown in (A). Hierarchical clustering confirms a clear separation of the different protocols. Genes not detected across samples were indicated with dark-blue color, at higher intensity than minimum expression levels.
(E) Results of snRNA-seq of iNGN2 neurons, BMWi neurons, BMWi NPCs of day 6, and hiPSCs (hiPSC_3). Samples of the final neurons were taken 14 days after final replating of the respective protocol. In UMAP plots, the expression of some neural marker genes is shown. iNGN2 and BMWi neurons were also compared regarding neuronal subtype marker genes in a bubble plot.
(F) Development of electrophysiological activity of the iNGN2 and BMWi neuron cultures. Medium of the neurons replated in NMM- was switched to NMM+ after 7 days. The mean firing rate of the action potentials (Hz) is plotted against the days after the final replating, across 5 different cell lines (hiPSC_1–5) each with 6 technical replicates (results are shown as means ± SEM). The activity of the neurons was recorded for 8 min. Insert shows hiPSC_5 neurons on MEA plate (day 29, NMM-).
(G) Network activity pattern of the MEA measurements from (F). 2-min sections are shown (day 49, hiPSC_4).

midbrain, neural crest), we observed a clear separation between the iNGN2 and BMWi protocol, both at the replating step and neuron stage. This was mainly due to differences in telencephalic region marker genes (higher in BMWi) and peripheral neuron marker genes (higher in iNGN2). For a subset of these genes, strong effects of the BMWi protocol, highly comparable across hiPSC lines (hiPSC_1–3), were observed (Figures 3B and 3C). As expected, hindbrain and spinal cord genes were strongly increased in NMM+ medium in the final neurons. Interestingly, switching from NMM- to NMM+ medium 1 week after replating in the BMWi condition did not lead to separate clustering (Figure 3A), indicating weak effects at this stage. When examining other subtype neuron-specific marker genes (adrenergic, cholinergic, dopaminergic, GABAergic, glutamatergic, noradrenergic, and serotonergic), the BMWi neuron population consists mainly of glutamatergic neurons with a proportion of GABAergic (larger than in iNGN2), adrenergic, and cholinergic (but less than in iNGN2) neurons (Figures S6D and S6E).

To compare the telencephalic-patterned BMWi neurons against dSMADi neurons, telencephalic transcriptome analysis was performed. Stability of regional identity over time, 2-, 3-, and 4-week matured neurons, was assessed (Figures 3D and S6F). The data presented in Figure 2C indicated the expression of VGLUT2 (SLC17A6) besides VGLUT1 (SLC17A7). To assess the expression of both markers over time, along with *LHX9*, neurons generated with BMWi and dSMADi protocols 3 (BMWi) and 4 weeks (both) post plating were compared to neurons of the respective protocol at 2 weeks. For both cell types, *SLC17A6* and *LHX9* showed lower expression levels at later time points, whereas the expression of *SLC17A7* was increasing (Figures S6G and S6H).

Different time points did not otherwise lead to strong differences in regional marker expression. Between BMWi and dSMADi neurons, the latter showed a stronger expression of GABAergic markers (Figure S6I). Otherwise, no strong difference in subtype marker expression can be seen in differential gene expression and comparison in Pearson correlation (Figure S6J). The BMWi neurons matured for 2 and 4 weeks show no significant differences in gene expression of selected subtype marker genes. It is only noticeable that as maturation progresses, residual expression of *NGN2* decreases (Figure S6K). If comparing the 3 different maturation points using a Pearson correlation, no strong changes can be detected due to long-term culture (Figure S6L). To correlate BMWi and dSMADi cultures to brain regions, the Genotype-Tissue Expression (GTEx) portal gene expression data were compared to our RNA-seq data. BMWi neurons exhibit a significant overlap with telencephalic/forebrain regions, similar to dSMADi samples. This finding leads us to conclude that the population represents a section of telencephalon (Figure S6M). It is not surprising that individual hiPSC lines give slightly different results in terms of regionality/exact composition of neuron subtypes (Kim et al., 2024; Volpato and Webber, 2020). Based on this RNA-seq data, BMWi and dSMADi protocols show only minor differences in the final neurons, even when working with 3–4 different donor lines, respectively.

By single-nuclei RNA-seq (snRNA-seq) analysis of hiPSCs, BMWi NPCs of day 6, iNGN2 neurons, and BMWi neurons (final neuron samples were harvested 14 days after final replating), we could demonstrate that the BMWi protocol generates a completely different and distinct population of neurons (Figures 3E, S7A, and S7B for cluster analysis). Analysis of neuron subtype-specific markers confirmed the previous observation of a predominantly glutamatergic (*SLC17A6*, *SLC17A7*, and *GRIN2B*) neuron population, with a stronger GABAergic (*GABRA2*, *GABRA5*, and *GAD1*) but less cholinergic (*SLC18A2* and *CHAT*) population, that also expresses few PNS/hindbrain markers (*ISL1*, *PRPH*, *POU4F1*, *HOXB5*, *PIEZO2*, and *HOXA1*). As *LHX9* is also expressed in the hypothalamus, we evaluated the expression of selected hypothalamus regional markers (*NKX2-1* [not plotted, as no expression detectable], *NKX6-1*, *HCRT*, *PNOC*, and *LHX6*) showing largely negative cells. In general, both protocols do not yield a strong serotonergic, (nor-) adrenergic, or dopaminergic component. Due to the gamma-secretase inhibitor treatment and mitotic inactivation, the neuron cultures were homogeneous, and also by snRNA-seq analysis, few to no non-neuronal cells could be identified at the mature neuron stage (hiPSC: *POU5F1* and *NANOG*; radial glia/neural stem cells: *SOX1*, *GLI3*, and *SLC1A3*; astrocytes: *SOX9*, *GFAP*, *AQP4*, and *NFIB*; oligodendrocytes: *CSPG4* and *OLIG2* (not detected); Figures 3E and S7A).

To assess the electrophysiological function of our hiPSC-derived neurons, multielectrode array (MEA) experiments were performed with 5 hiPSC lines in the absence of astrocytes. Cells were plated either directly in NMM+ medium or in NMM- medium and switched to NMM+ after 1 week, to promote maturation and function (Manos et al., 2022) and allow comparable medium conditions at the time of measurement. Firing activity was first observed at 17 days after replating, and most conditions reached peak activity by 40 days that was maintained until day 63 (end of the experiment). The condition that showed the highest activity is BMWi plated in NMM- and switched to NMM+ after 1 week (NMM-/+) (Figure 3F). iNGN2 neurons showed a random firing pattern after 49 days of maturation (Figure 3G), whereas BMWi NMM-/+ neurons exhibited a pattern that resembles network bursting, as previously reported from primary or hiPSC-derived telencephalic cultures (Cossart et al., 2003; Kamioka et al., 1996; Saberi-Moghadam et al., 2018; Sanchez-Vives and McCormick,

2000) and could also be observed in dSMADi neurons (Figure S7C). This is less apparent in BMWi neurons that were directly plated in NMM+. However, the switch from NMM- to NMM+ cannot be the only factor for the network pattern, as iNGN2 with the same paradigm shows overall less network activity (Black et al., 2018; Eaton et al., 2021). The network burst rate development was comparable to the mean firing rate (Figure S7D).

BMWi neurons show aggregation of tau protein comparable to dSMADi-derived telencephalic neurons

In AD and other tauopathies, intraneuronal accumulation of hyperphosphorylated tau leads to the formation of neurofibrillary tangles (NFTs) (Garcini et al., 1986; Grundke-Iqbal et al., 1986; Kidd, 1963; Seubert et al., 1995). The increase of NFTs correlates with cognitive decline in AD (Karran and De Strooper, 2022). We previously developed a disease-relevant hiPSC-derived system to aggregate endogenous tau protein in a tauopathy-like manner. For that, hiPSC-derived telencephalic neurons were seeded with sonicated paired helical filaments (sPHFs, derived from 2N4R P301L recombinant tau using heparin) leading to a dose- and time-dependent increase of insoluble MC1 (a conformation-dependent antibody)-positive tau aggregates (Manos et al., 2022). This model uses hiPSCs that were genetically modified to express the MAPT P301S mutation, assisted by intronic mutations (E10 + 14/E10 + 16) which enable inclusion of exon 10. A subclone was generated inserting an iNGN2 cassette in the AAVS1 locus. This allowed comparison of telencephalic neurons seeded with tau sPHFs, differentiated either with the standard dSMADi protocol or BMWi protocol, from the same hiPSC background line. In both neuron cultures, sPHFs were added 2 weeks after final plating, and analysis was performed 3 and 4 weeks post seeding. Neurons were fixed with methanol to remove soluble tau, and insoluble tau aggregates were stained with MC1. Results demonstrate comparable levels of endogenous tau aggregation in dSMADi and BMWi neuron cultures when normalized to the number of healthy nuclei, which is time- and concentration dependent (Figure 4). No cell loss was observed in both neuronal differentiation systems assessed by the number of healthy nuclei.

These results indicate that BMWi neurons are suitable to model an important aspect of tauopathies *in vitro* like what has been shown before in telencephalic neurons derived from the dSMADi protocol (Hong et al., 1998; Liu and Gong, 2008; Manos et al., 2022; Schoch et al., 2016).

BMWi protocol can be combined with regional patterning cues for the CNS

For modeling diseases other than AD, a change in the regional identity was evaluated. To assess dorsoventral patterning of cells generated by the BMWi protocol, we used increasing concentrations of the ventralizing SHH agonist SAG. Dorsalization was studied by increasing concentrations of the GSK3β inhibitor CHIR99021 (“CHIR”), which is activating canonical WNT signaling (Figure 5A). To assess ventralization, we evaluated the expression of the ventral marker gene *NKX2.1* and the floorplate marker *FOXA2* 4 days post induction with BMWi plus morphogen. Both genes showed a dose-dependent increase in expression when treated with SAG compared to hiPSC or CHIR-treated controls. In contrast, dorsal markers *IRX3* and *PAX3* were both increased in the CHIR-treated conditions (Figure 5B). These results indicate that the BMWi-derived NPCs can be further patterned along the dorsoventral axis.

As *FOXA2* was induced on the mRNA level, BMWi cultures alone or in combination with SAG were analyzed by IF staining for FOXA2 and the neuroectodermal marker PAX6. Strikingly, the treatment with SAG caused a complete absence of $PAX6^+$ cells, whereas it induced strong FOXA2 immunoreactivity as early as 4 days after induction (Figure 5C). This is of particular importance, as the floorplate is the only region in the developing neural tube that does not express PAX6, but FOXA2, indicating induction of floorplate progenitor cells (Fedele et al., 2017; Kirkeby et al., 2012; Lek et al., 2010; Smits et al., 2019).

Midbrain dopaminergic neurons (mDANs), which are affected in Parkinson’s disease, are developed from midbrain floorplate progenitor cells (Kirkeby et al., 2012) that are patterned by exposure to WNT and SHH signaling and express a combination of midbrain markers, such as *OTX2* and *LMX1A* and the floorplate marker *FOXA2*. We therefore applied WNT and SHH patterning similar to that described by Kirkeby et al. (2012) and Xu et al. (2022) during BMWi patterning in 3 independent hiPSC lines followed by NGN2 overexpression and maturation (Figure 6A). This led to the expression of *FOXA2*, *LMX1A*, and *OTX2* at the progenitor stage (hiPSCs already express *OTX2*); the latter was significantly downregulated during further maturation. Furthermore, the expression of tyrosine hydroxylase *TH*, the pace-making enzyme for dopamine synthesis, as well as the dopa decarboxylase *DDC* was upregulated with increasing maturation time (Figure 6B). IF staining confirmed the presence of TH^+ and $LMX1A^+$ neurons, indicating that floorplate progenitors induced by BMWi/WNT/SHH can be further differentiated to ventral midbrain neurons (possibly mDAN-like) following iNGN2 (Figure 6C, for quantification, see Figure S7E). To confirm the patterning effect, ventral midbrain BMWi neurons were compared with sBMWi and BMWi neurons (Figure S7F).

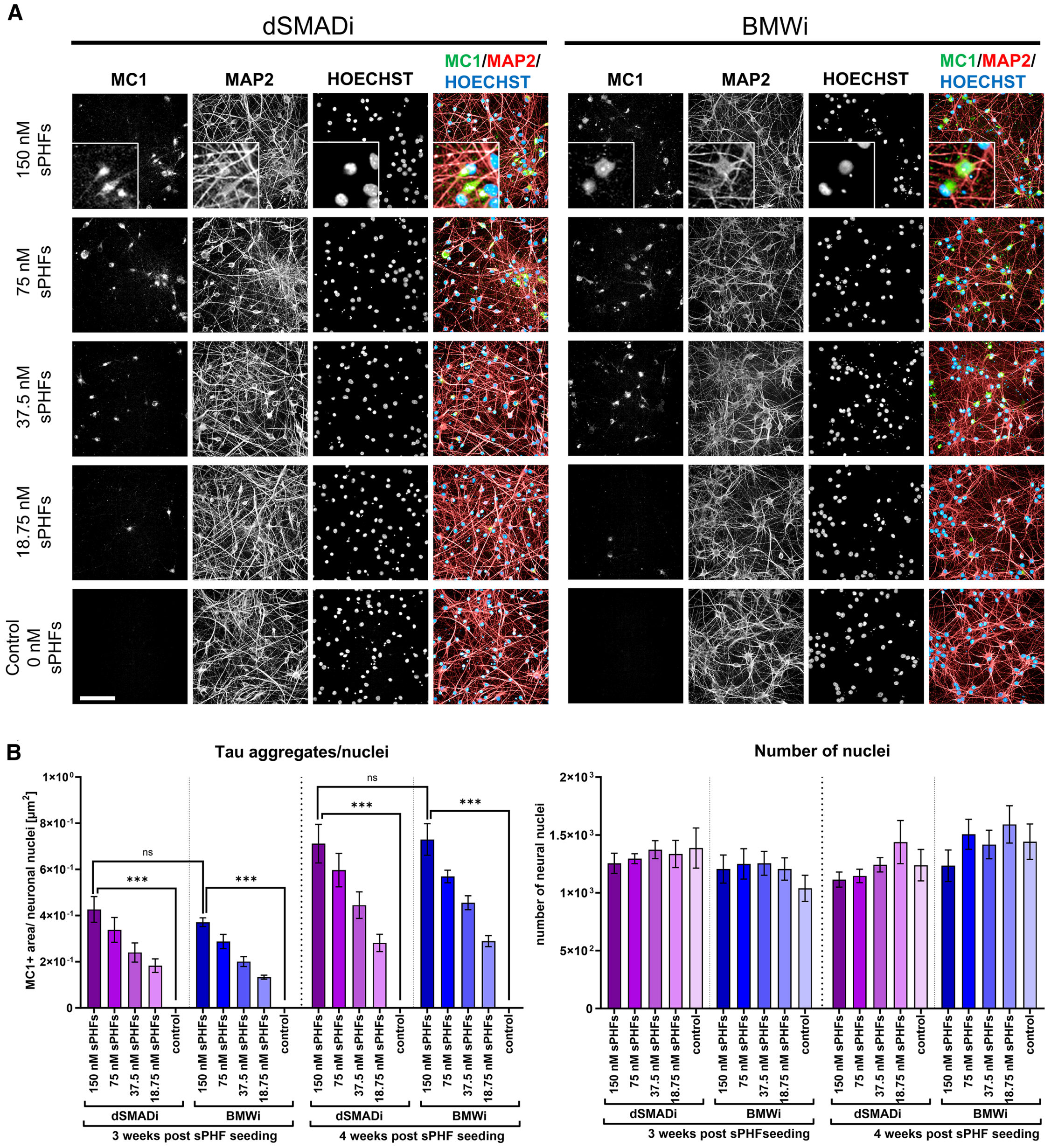

Figure 4. Tau seeding in telencephalic neurons

(A) Representative IF images of tau sPHF-seeded telencephalic neurons of the BMWi and dSMADi protocol 4 weeks post seeding stained with MC1 and pan-neuronal marker MAP2 (scale bars: 100 µm).

(B) Quantification of tau aggregates (MC1-positive area [µm2]) and number of healthy nuclei in dSMADi and BMWi neurons 3 and 4 weeks post seeding with sPHFs. The number of healthy nuclei and the MC1-positive area (μm^2) were determined using high-content imaging (means ± SEM from N = 4 independent experiments with each n = 3 replicate wells) (*$p < 0.05$, **$p < 0.01$, ***$p < 0.001$).

Of note, we also tested the application of SHH inhibitors (cyclopamine 5 µM, vismodegib 1 µM) during the BMWi treatment and did not observe an effect on SHH target genes *GLI1* and *FOXA2* (not shown), which were only activated when additional SHH agonist SAG was added. This suggests that at least the hiPSC lines used for this study do not have endogenous SHH signaling present in this differentiation. The inhibitors were well tolerated and could be added, if needed.

We evaluated whether BMWi could be combined with SAG and RA to pattern NPCs to a ventral posterior fate that could give rise to ventral spinal cord/MNs following

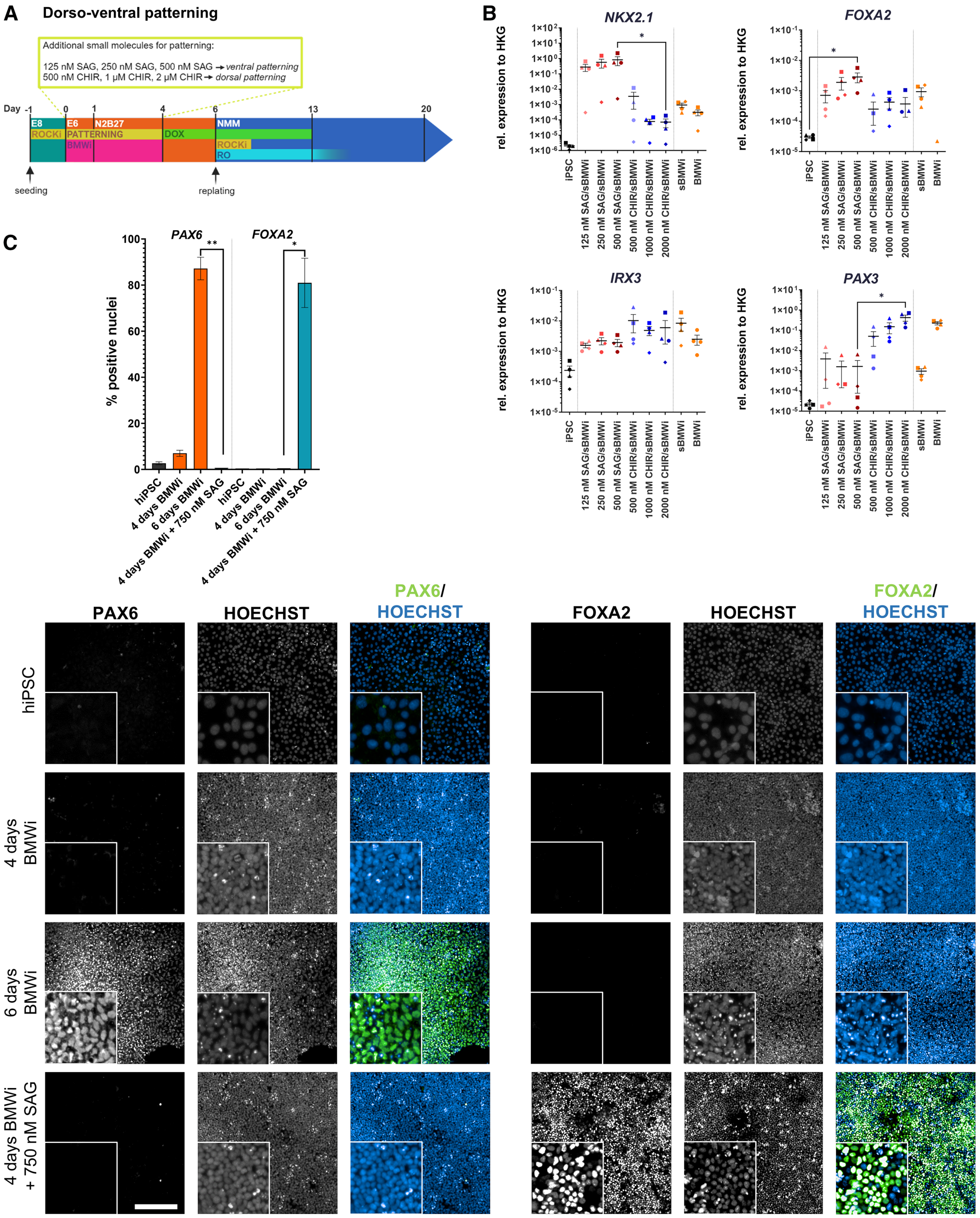

Figure 5. Patterning during BMWi treatment

(A) Schematic representation of the BMWi protocol with additional patterning.

(B) Gene expression of different regional genes to assess patterning along the dorsoventral axes. The experiments were carried out with 4 different cell lines (hiPSC_1–4, means ± SEM) and normalized to expression of housekeeping genes.

(C) Representative IF images (hiPSC_3) of NPCs expressing either the neuroectodermal precursor marker PAX6 or the floorplate marker FOXA2 with additional ventral patterning by SAG (scale bars: 200 μm, insert 3× zoom-in). sBMWi and BMWi NPCs on day 4/day 6 are shown. Quantification of PAX6 and FOXA2 of 3 cell lines is also shown (hiPSC_1,2,4, means ± SEM) (*$p < 0.05$, **$p < 0.01$, ***$p < 0.001$; ● = hiPSC_1, ▲ = hiPSC_2, ■ = hiPSC_3, ◆ = hiPSC_4, ★ = hiPSC_5).

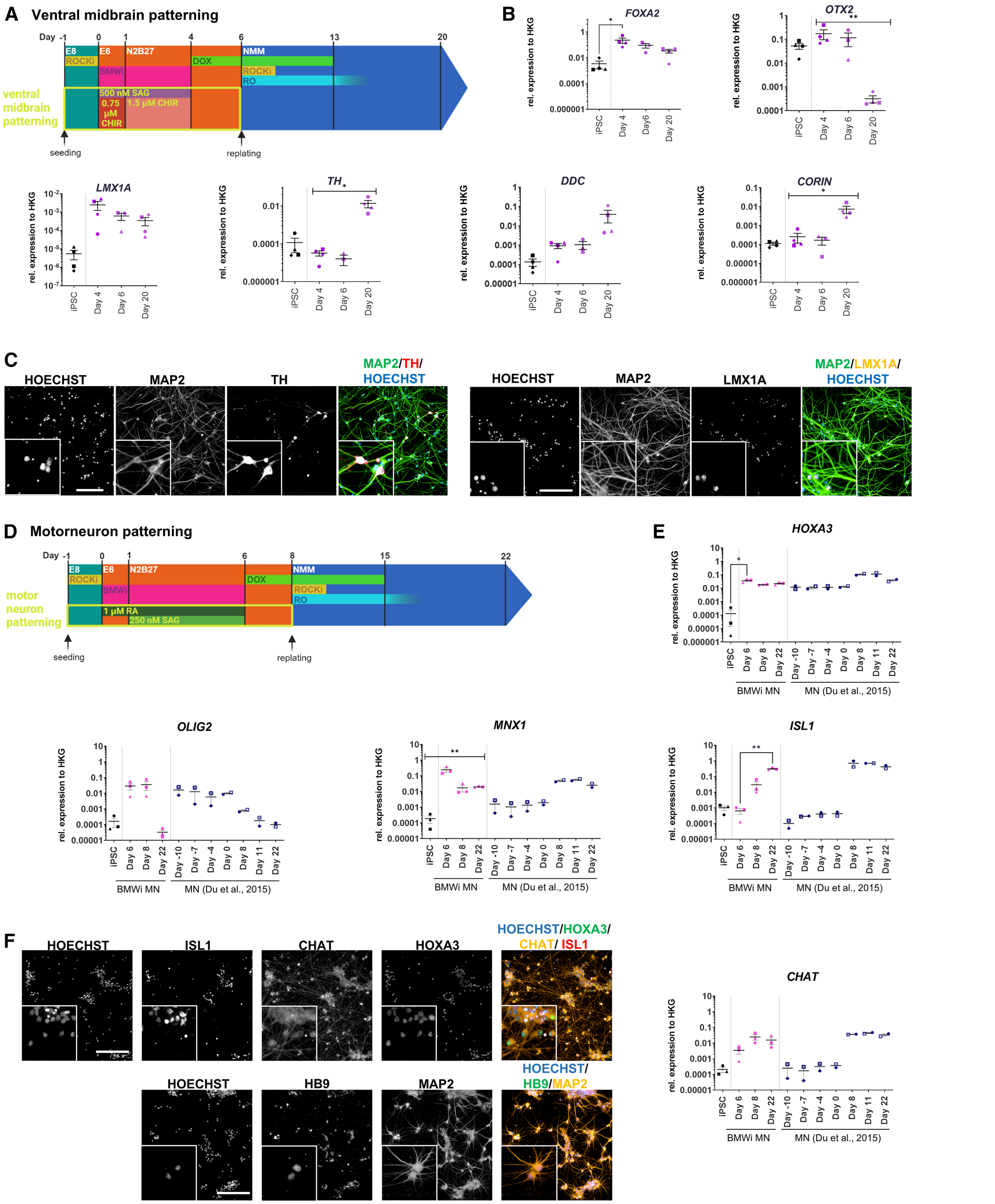

Figure 6. Patterning of neuronal subtypes

(A) Schematic representation of ventral midbrain BWMi patterning.
(B) Gene expression of relevant marker genes for ventral midbrain ($N = 4$ cell lines, hiPSC_1–4).
(C) Representative IF images (day 20, hiPSC_3) of ventral midbrain-patterned neurons.
(D) Schematic representation of the BWMi MN protocol.

(legend continued on next page)

iNGN2 (Figure 6D). Upon differentiation of three hiPSC lines, we could observe a very strong upregulation of the posterior marker *HOXA3* and the MN progenitor marker *OLIG2*. Upon maturation in NMM+ medium, *OLIG2* was downregulated, whereas other MN markers, *MNX1* (coding for HB9), *ISL1*, and *CHAT* (choline acetyltransferase) were upregulated. Comparison of BMWi/SAG/RA + iNGN2 neurons ("BMWi MN") to two independent derivations of MNs derived according to Du et al. (2015) showed a comparable expression pattern in the final MN populations (Figure 6E). IF analysis showed that BMWi MN neuronal cultures expressed the pan-neuronal marker MAP2, MN markers such as HB9, CHAT, and ISL1, as well as the caudal marker HOXA3, indicating an MN-like identity (Figure 6F; for quantification, see Figure S7G). To assess the effect of the patterning on the resulting neurons, cultures of BMWi MN were compared with corresponding cultures with the sBMWi or BMWi (telencephalic) protocol (Figure S7H).

Derivation of PNS neurons by stimulation of WNT signaling

We showed that robust derivation of telencephalic cells requires WNTi for a broadly applicable neural induction based on BMPi/MEKi (Figure 1). Consequently, we speculated that activation of WNT signaling by CHIR could result in the specification of neural crest progenitor cells that express SOX10 (Carney et al., 2006). We therefore omitted IWP2 from the neural induction paradigm ("BMi") and included 3 μM CHIR, as well as 1 μM RA, to achieve an additional posteriorization, with the intention to generate dorsal root ganglia (DRG)-like sensory neurons (Figure 7A, sensory BMi protocol [sensBMi]).

Applying the sensBMi protocol results in progenitor cultures that exhibit a strong and homogeneous expression of SOX10 at the expense of PAX6, which was not detected on protein level, indicating the formation of neural crest-like tissue (Figure 7B; for quantification, see Figure S7I). 5 hiPSC lines were differentiated with this paradigm, followed by iNGN2 and plating in NMM+ medium. On the mRNA level, we observed the expression of key peripheral neuron markers such as *PRPH*, *ISL1*, *POU4F1* (BRN3A), *PRDM12*, and *NTRK1* as well as the pan-neuronal marker *MAPT* (Figure 7C). These neurons showed strong upregulation of HOX genes compared to the undifferentiated hiPSCs, with the strongest upregulation of *HOXB4* and *HOXB5* over hiPSCs. IF staining revealed that sensBMi neurons were positive for sensory neuron markers TRPA1, PRDM12, BRN3A, and ISL1 (Figure 7D; for quantification, see Figure S7J). To assess these cells in a functional assay, MEA experiments were performed comparing the standard iNGN2 differentiation with the DRG-like paradigm and treating the cells with capsaicin ("CAP"), a neurotoxin and activator of nociceptive sensory neurons (Yang and Zheng, 2017). MEA analysis using 5 hiPSC lines indicated an increase of activity in cultures treated with 100 nM CAP compared to solvent only (Figure 7E). This effect was approximately 2.5× more pronounced in DRG-like cultures. To assess the effect of the patterning on the resulting neurons, cultures were compared with corresponding cultures with the sBMWi or BMWi (telencephalic) protocol (Figure S7K).

Together, these data indicate that neural crest progenitors can be derived by the sensBMi protocol, which mature into neurons expressing markers and showing functionality of peripheral, sensory neurons.

DISCUSSION

Controlled NGN2 overexpression in hiPSCs is an attractive source of functional neurons due to the ease of use and short timeline (Wang et al., 2017). However, the initial assumption that these always represent neurons with a telencephalic, excitatory (glutamatergic) identity (Zhang et al., 2013) has recently been scrutinized (Chen et al., 2020; Lin et al., 2021). Studies using iNGN2 either did not reveal basal *FOXG1* expression in the unmodified iNGN2 condition (Limone et al., 2023; Nehme et al., 2018; Wang et al., 2017), or report its absence in most of the samples at the mRNA level ((Mertens et al., 2021), own analysis of deposited data). While the original report from Zhang and colleagues showed *FOXG1* gene expression, this seemed limited to samples co-expressing *SOX2*, suggesting expression from remaining neural progenitor cells that follow the developmentally predetermined program that is recapitulated *in vitro* and will form telencephalic neural progenitor cells first (Shi et al., 2012). A complicating aspect in comparing studies is that some studies used viral delivery with random integration of the iNGN2 genetic constructs whereas others, including our study, use stable induced pluripotent stem cell (iPSC) lines with targeted integration into a safe harbor locus. Additional contribution to varying results could arise from using different pluripotent stem cell lines that have been

(E) Comparison of gene expression at different time points of the BMWi MN protocol (*N* = 3 cell lines, hiPSC_1–3) and the protocol for differentiation of MN according to Du et al (hiPSC_1,8).
(F) Representative IF images of BMWi MN (day 22, hiPSC_1) (all scale bars: 200 μm, insert 3× zoom-in, all results normalized to housekeeping gene expression, means ± SEM) (*$p < 0.05$, **$p < 0.01$, ***$p < 0.001$; ● = hiPSC_1, ▲ = hiPSC_2, ■ = hiPSC_3, ◆ = hiPSC_4, ☐ = hiPSC_8).

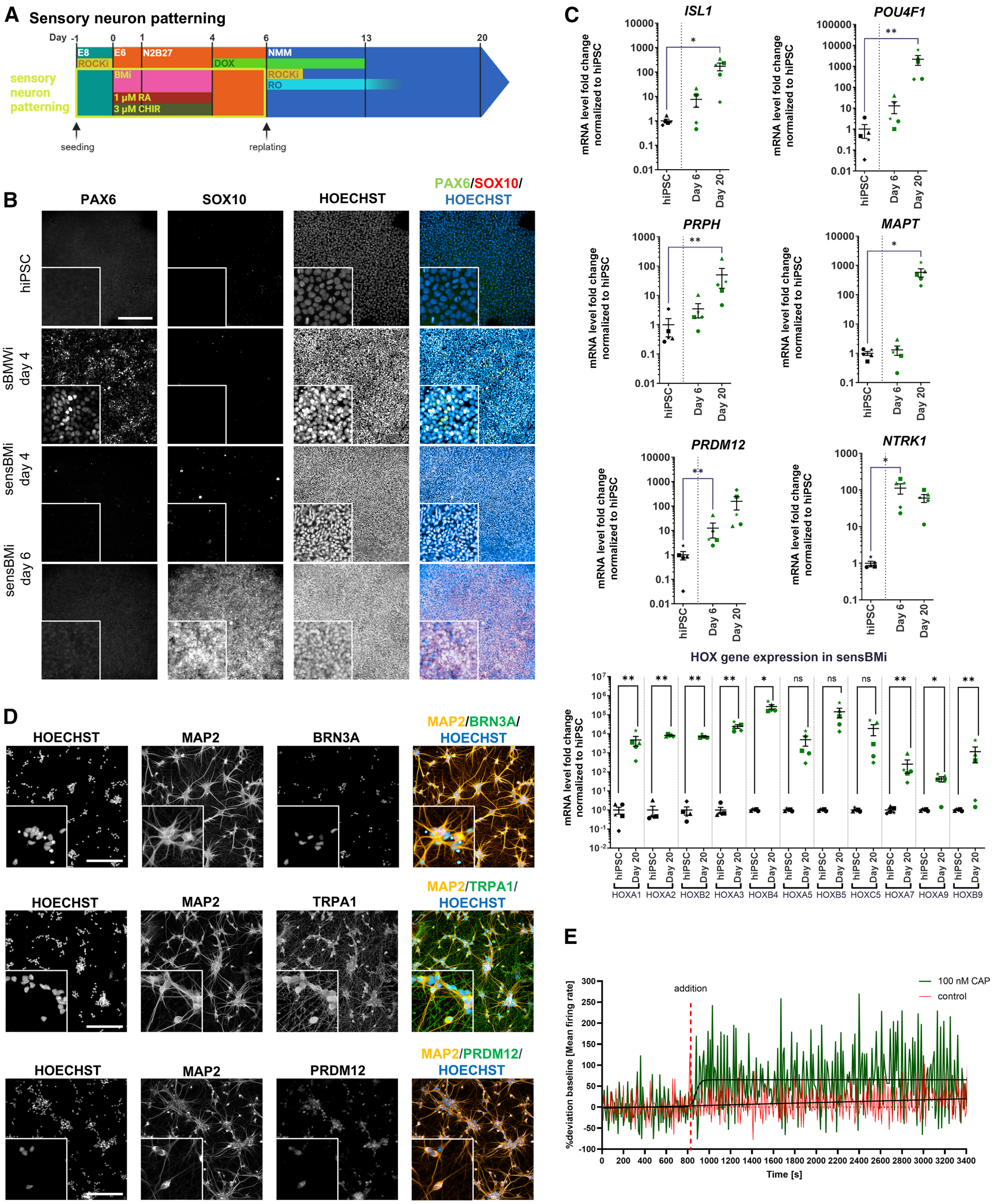

Figure 7. Patterning of DRG

(A) Schematic representation of the shortBMi with sensory neuron patterning protocol (sensBMi).

(B) Representative IF images of the expression of sensory neuron progenitors after 4 and 6 days of the neural crest marker SOX10 (hiPSC_5, scale bars: 200 μm, insert 3× zoom-in).

(C) Gene expression of relevant marker genes for sensory neurons normalized to average expression in hiPSCs (N = 5 different cell lines, hiPSC_1–5, means ± SEM).

(D) Representative IF images of the expression of sensory neuron marker genes on day 20 (hiPSC_5) (scale bars: 200 μm, insert 3× zoom-in).

(legend continued on next page)

reported to show different endogenous WNT signaling (Moya et al., 2014; Strano et al., 2020). We assessed the expression of key genes and confirmed the low expression of the telencephalic marker gene *FOXG1* and the cortical marker *TBR1* in the iNGN2 protocol in hiPSC lines from 5 different donors. The low *FOXG1* and *TBR1* expressions were independent of the neural maturation medium (NMM+/−) that was used in this study. Interestingly, we detected the expression of *POU3F2* (BRN2) and *CUX1/2* in the iNGN2 neurons, markers which were used to attribute a telencephalic/telencephalic identity (Wang et al., 2017). Nevertheless, not arising from a telencephalic (FOXG1$^+$) progenitor and lacking the expression of *FOXG1* or other telencephalic markers in the resulting neurons, expression of *POU3F2 and CUX1/2 alone* could be misleading, as, for example, *CUX1/2* is already strongly expressed in the hiPSC. We therefore concluded that the iNGN2 protocol did not robustly result in telencephalic, CNS-patterned cultures and explored a strategy to rapidly commit hiPSCs to a telencephalic neural progenitor cell fate before initiating the iNGN2 expression.

The effects of FGF2 signaling in neural induction from hiPSCs were controversially discussed, and some protocols had included FGF2 on purpose (Cohen et al., 2010), until it was shown that inhibition in addition to dSMADi greatly accelerates neurogenesis but yields sensory neurons of the PNS (Greber et al., 2011). Later protocols used this triple inhibition with modulation of WNT signaling to generate CNS (Qi et al., 2017) and PNS progeny more rapidly (Chambers et al., 2012).

Confirming Greber et al. (2011), we could show that inhibition of the FGF2/MEK/ERK signaling pathway, in combination with commonly used dSMADi, led to a strongly accelerated induction of neuroectoderm. Unexpectedly, inhibition of the TGF-β/SMAD2/3 pathway was completely dispensable. Using BMPi/MEKi, some hiPSC lines exhibited differentiation toward a neural crest fate, indicated by SOX10$^+$ cells. As neural crest is induced by WNT signaling during development, addition of the PRCN inhibitor IWP2 (which inhibits the palmitoylation of WNT proteins, thus their production by the cells (Chen et al., 2009)) uniformly abolished neural crest contamination. It is worth noting that the resulting BMWi led to a comparable outcome of neural induction from different hiPSC lines, including a more homogeneous expression of *FOXG1* between hiPSC clones. This is in line with previous reports suggesting that different pluripotent stem cell lines can be differentiated more homogeneously to telencephalic neurons when WNT signaling is inhibited due to the posteriorizing effect of WNT (Moya et al., 2014; Strano et al., 2020). The inhibition of WNT also prevents mesodermal and endodermal fate (Kreuser et al., 2020); endogenous WNT signaling could also explain the predominantly neural crest differentiation outcome observed by Greber and colleagues. BMWi therefore represents a novel, minimal, but universally applicable neural induction paradigm and is largely independent of the cell density. It rapidly generates a telencephalic neural progenitor cell population, indicated by the strong expression of *PAX6* and *FOXG1*, without complicated daily variations of small molecules or recombinant proteins.

Selection of robust inhibitors was essential for establishing a reliable protocol. The BMP inhibitor LDN proved to be superior to DM, leading to reduction of *TFAP2A* expression (most likely non-neural ectoderm, also indicated by CK18$^+$ cells in mature cultures [not shown]) (Qu et al., 2017). IWP2 was superior to the frequently used WNT/tankyrase inhibitor XAV939. One possible explanation would be that IWP2 prevents production/secretion of WNT proteins, thus inhibiting canonical and non-canonical WNT signaling, while inhibition of tankyrase may only block canonical WNT signaling (Karner et al., 2010). An additional benefit of IWP2 is that using either pharmacological or protein activators of WNT signaling can be added on top to fine-tune, as we could demonstrate. It is not surprising that inhibition of FGF2/MEK necessitates complete blockade of BMP signaling, as these pathways play opposite roles in ectoderm development (Qu et al., 2017), and it was shown before that the effect of pathway activation can be dependent on the presence or blockade of other pathways (Rao et al., 2016). One of our goals was to accelerate the commitment of the cells to a telencephalic fate, which could benefit modeling of diseases such as AD. We showed that 4 days of BMWi was sufficient to induce RNA expression of PAX6, which could be uniformly detected at the protein level by day 6 (compared to dSMADi, which only showed emergence of a few PAX6$^+$ cells at this stage).

One unexpected result was the strong posteriorizing effect that early exposure to NMM containing B27 Plus had on cultures compared to NMM with B27 supplement without retinoids. A possible explanation could be a higher retinoid (such as RA or all-*trans*-RA) concentration in B27 Plus, which we hypothesize based on the orange color of the supplement. RA has been shown to induce differentiation of stem cells in general, induce maturation from neuronal progenitor cells specifically, and act as a powerful

(E) MEA assay of sensory sBMi neurons 3 weeks after replating (N = 5 different cell lines each n = 2/3 technical replicates, hiPSC_1–5). 100 nM CAP diluted in NMM+ was added during measurement. As control, just NMM+ with solvent was added (*p < 0.05, **p < 0.01, ***p < 0.001; ● = hiPSC_1, ▲ = hiPSC_2, ■ = hiPSC_3, ◆ = hiPSC_4, ★ = hiPSC_5).

antioxidant (Durston et al., 1989). While these features are desirable for neuronal maturation, RA has also been shown to induce posteriorization of NPCs (Calder et al., 2015). Therefore, even though NMM+ containing B27 Plus very strongly supported neuronal health, maturation, and function, we caution its use when neurons are not yet fully committed to the desired regionalization. Compared to cultures only cultivated in NMM-, we did not observe strong differences in gene expression when switched to NMM+ at 1 week post plating, most likely, as the cells are not amenable to regional patterning cues anymore. This offered excellent maturation and electrophysiological activity, as shown in our tau seeding and MEA experiments, respectively. It is worth noting that previous studies, using iNGN2 paradigms, suggest co-culturing hiPSC-derived neurons with astrocytes to obtain fully active cultures (Kirkeby et al., 2012; Limone et al., 2023; Nehme et al., 2018; Neyrinck et al., 2021; Shih et al., 2021), whereas our culture system allowed the generation of functional neurons alone. We did not detect astrocyte-like cells in our cultures (GFAP staining, not shown) and other non-neuronal cells (see also Figure S7A), which also would be prevented by the antimitotic mitomycin C treatment routinely applied for obtaining pure, long-term stable neuron cultures. The propensity of BMWi-induced early neuroepithelium to differentiate into non-neuronal cells (astrocytes or oligodendrocytes) would need to be assessed. Still, after 6 days of BMWi, cells expressing progenitor markers, such as SOX9, could be identified; thus, astrocyte differentiation potential could exist (Sun et al., 2017).

The BMWi approach followed by iNGN2 represents a unique and highly attractive strategy as compared to other techniques for deriving neurons arising from telencephalic progenitors. Comparable protocols used inhibition of SMAD1/5/8 and SMAD2/3 signaling pathways (dSMADi) to induce a neural progenitor cell fate. Other studies had addressed guiding the cells to a more telencephalic fate together with iNGN2 and had combined dSMADi, with or without additional patterning factors, reporting a stronger CNS outcome (Chen et al., 2020; Nehme et al., 2018). The combination of dSMADi with XAV as in the study by Nehme et al. did not result in a strong upregulation of *FOXG1* in our experiment, in line with a very recent study by the authors that showed an increase of *FOXG1* mRNA expression by an average 2-fold over the iNGN2 condition (Limone et al., 2023; Nehme et al., 2018), as well as no increase of *TBR1* expression, compared to an increase of approximately 100-fold in our experiments. One possible explanation is an incomplete conversion of the cells to a homogeneous telencephalic progenitor fate prior to iNGN2. Additionally, the use of XAV instead of IWP2 could lead to less WNT inhibition. Chen et al. used a 6-day pre-differentiation paradigm prior to NGN2 induction, but with dSMADi alone. In our experiments, this time with dSMADi was insufficient for a PAX6$^+$ progenitor population, in line with other studies (Chambers et al., 2009). Applying these dSMADi conditions instead of BMWi for 6 days in our protocol led to neuron cultures expressing similar levels of *TBR1*, but less *FOXG1*, as well as more *PRPH* and *HOXA1*, compared to the BMWi induction, possibly due to incomplete commitment to telencephalic neuroectoderm in the time applying dSMADi. Walsh and colleagues used a more timed approach combining a staggered dSMADi: 1 day a pan-FGF2 inhibitor (BGJ) with LDN, followed by 1-day inhibition of SMAD2/3 signaling by A8301 (TGF-β type 1 receptor ALK5 kinase inhibitor), WNT signaling with wntC59, as well as addition of FGF2 (Walsh et al., 2020). While this approach shares similarities to our induction, it lasts only 2 days (4 with regional patterning), uses a SMAD2/3 inhibitor, and involves a postulated early/intermediate neuroectodermal state. Also, their accelerated neural induction was not combined with the iNGN2 strategy. Using 5 different hiPSC lines that were adapted to their culture conditions, we obtained approximately 10-fold less *PAX6* expression on mRNA level at the end of this induction phase (we observed comparable levels on mRNA after 3 days of BMWi), did not observe homogeneously PAX6$^+$ cells, and experienced apparent cell loss. This could be partially attributed to the effect BGJ had on phosphorylation of PLCγ, thus inhibiting cell proliferation and survival signaling. We speculate that the transcriptional changes caused more downstream of the FGF2 receptor by applying the MEK inhibitor PD in the BMWi approach are sufficient to accelerate neural induction without negatively affecting cell survival. Furthermore, the hiPSC lines that we used could differentiate slower or faster than needed for the exactly timed protocol by Walsh and colleagues. In contrast, the BMWi protocol simply uses one paradigm for 4–6 days. This could facilitate a broader applicability, as no variations of inhibitors are applied.

Direct comparison using same hiPSC clones differentiated with dSMADi or BMWi protocols using RNA-seq, also including additional time points after replating, showed that the composition of neuronal subtypes was stable over time. The analysis also showed that the BMWi protocol generates a population of neurons with a comparably strong signature of excitatory/glutamatergic markers but less inhibitory/GABAergic markers (still more than in the iNGN2 protocol), with a slightly lower expression of dopaminergic but slightly stronger cholinergic (but less than in the iNGN2 protocol) marker gene expression. Mapping the expression data from neurons generated with the two protocols to GTEx (Lonsdale et al., 2013) brain region data showed a comparable, predominantly telencephalic

component between the BMWi and dSMADi samples. snRNA-seq analysis directly comparing the resulting neurons from iNGN2 and BMWi protocols further confirmed that BMWi results in a distinct, separate population when compared to iNGN2. Analyzing this dataset for the expression of neuronal subtype-specific markers further confirmed the observation obtained by the bulk RNA-seq analysis. BMWi neurons predominantly have an excitatory/glutamatergic phenotype, with a stronger GABAergic population compared to iNGN2 neurons. Less BMWi neurons were positive for peripheral or sensory neuron markers, such as *PRPH*, *ISL1*, and *POU4F1*. Both BMWi and dSMADi neurons showed expression of *LHX9* but less in BMWi neurons. LHX9 is a marker that is associated with hypothalamic neurons (Kim et al., 2020) but also with the developing cortex (Bertuzzi et al., 1999; Rétaux et al., 1999). As expression of *LHX9* is decreasing during maturation and other hypothalamus-related (regional) genes (*NKX2.1*, *NKX6.1*, *HCRT*, *LHX6*, and *PNOC*) (Kim et al., 2020; 2021; 2022; Maroof et al., 2013) were not expressed (also at progenitor stage), this is possibly reflecting the role in cortical development. It should be noted that the brain region mapping pattern compared to the GTEx datasets shows a similar hypothalamic component in the reference dSMADi protocol samples. When comparing BMWi neurons 14 days post final plating with those that have matured an additional 1 to 2 weeks, there is a decrease in the expression of *SLC17A6*, while *SLC17A7* increases (Berry et al., 2012; Bertuzzi et al., 1999; Boulland et al., 2004; Rétaux et al., 1999; Togo et al., 2021). This further indicates a maturation process in the BMWi neurons, a pattern that is also observed in dSMADi cortical neurons. While the network bursting observed in the MEA experiment is similar to reports from telencephalic cultures (Cossart et al., 2003; Kamioka et al., 1996; Saberi-Moghadam et al., 2018; Sanchez-Vives and McCormick, 2000), more detailed electrophysiological assessment would be needed to attribute a true cortical identity using this method.

During BMWi, cells can be easily regionally patterned. Followed by iNGN2, this led to the formation of neurons that were derived from the ventral midbrain, similar to mDANs; as well as MNs. Here, the reduction of WNT production by IWP2 enables the application of CHIR (as used for the ventral midbrain protocol) or even recombinant WNT proteins to tune the WNT signaling. The MNs that are formed were positive for markers ISL1, MNX1/HB9, as well as HOXA3 and expressing caudal *HOX* genes. This could indicate that they are comparable to MNs of the brachial lower motor column (Dasen et al., 2005). Nevertheless, more careful titration may be required to promote the highest yield of mDANs or specific MN subpopulations from hiPSCs (Johns and Maragakis, 2022; Kirkeby et al., 2012). When replacing IWP2 with high doses of CHIR, the resulting BMi condition efficiently generates neural crest tissue and, combined with posteriorization, can be guided into a DRG-like neuron outcome.

Recently, Limone et al. (2023) and Sheta et al. (2022) published protocols suggesting that iNGN2 can be combined with patterning cues to generate MN or mDAN-like cells, respectively. Even though these protocols share principles with our study, they do not use the defined prepatterning approach unique to BMWi, as they either use dSMADi (Limone et al., 2023) or make use of a commercially available kit with unknown constituents, and the neurons are dependent on continuous expression of NGN2 (Sheta et al., 2022).

Lastly, we emphasize that all differentiations (unless daily sampling as required for a time course) were performed in a weekend-free manner; the protocols only require one (re) plating step—which is beneficial for large or repetitive experiments—and the progenitor cells can be frozen and thawed at the time of replating, which will allow less experienced users to generate neurons. The differentiation into CNS is not negatively affected by low plating densities, unlike reported for the dSMADi method, where high cell densities are required for formation of CNS over PNS cells (Chambers et al., 2009; Manos et al., 2022; Münst et al., 2018). This together allows the generation of large batches for screening approaches or repetitive experiments.

The BMWi neural induction paradigm therefore represents a robust, universally applicable method, independent of the commonly used SMAD2/3 inhibition to differentiate hiPSCs into regionally specifiable neuroectoderm. This can be of particular interest if combined with the iNGN2 paradigm, as shown here, can also be applied to obtain mechanistic insights, if an alternative to dSMADi is needed for experimental purposes. Although it was not assessed in this study, the BMWi paradigm should also represent an interesting base for further maturation of the cultures without iNGN2.

METHODS

hiPSC culture conditions

hiPSC lines used in this study were publicly available hiPSC lines with an iNGN2 cassette (BIONi010-C-13, available from EBiSC www.ebisc.org. The EBiSC Bank acknowledges Bioneer A/S as the source of hiPSC line BIONi010-C-13, which was generated with support from EFPIA companies and the European Union (IMI-JU)); hiPSC lines generated from Schöndorf et al. (2019) (BiomedX) with subsequent integration of an iNGN2 cassette; subclones of iPSC0028 (SIGi001-A, Sigma-Aldrich), one with subsequent

integration of iNGN2 cassette; or from IMI StemBANCC (Volpato et al., 2018). The complete list of hiPSC clones used can be found in Table S1.

Unless otherwise stated, hiPSCs were cultured in E8 Flex medium (Thermo Fisher Scientific) on Matrigel-coated (Manos et al., 2022) (Matrigel hESC-Qualified Matrix, LDEV-free, Corning) cell culture ware. Matrigel was coated in DMEM/F12 (Thermo Fisher Scientific). Medium was changed every second to third day, depending on the cell density and cells routinely split twice a week using Versene/EDTA (Lonza) as clumps, or as single cells with Accutase (Thermo Fisher Scientific) supplemented with 10 μM Y-27632 (Merck, referred to as ROCKi) for 10–15 min at 37°C. The cells were replated on Matrigel-coated cell culture ware in E8 flex medium with 10 μM ROCKi. ROCKi was removed the following day.

Neural induction using overexpression of NGN2

The protocol used is from Manos et al. (2022) and Stolzenburg et al. (2023). In all iNGN2 hiPSC lines used, a gene cassette was integrated into the AAVS1 (*PPP1R12C* gene) safe harbor locus by a contract research organization. hiPSCs were seeded on Matrigel-coated plates, as single cells at 52,000 cells/cm^2 in E8 flex medium with 10 μM ROCKi (day −1). On day 0, cells were washed once with N2B27 medium (Table S4) to remove E8 flex medium. Cells were then supplied with 0.5 mL/cm^2 N2B27 medium with 2 μg/mL DOX hydrochloride (Merck). On the next day (day 1), a complete medium change with the same medium was performed. On day 2, cultures were incubated for 15 min at 37°C with prewarmed Accutase with 10 μM ROCKi. Single-cell solution diluted 5× in N2B27 medium, centrifuged for 5 min at 300xg, and the pellet was resuspended in NMM (Table S5 [B27+ culture system: NMM+; B27 culture system: NMM-]) supplemented with 2 μg/mL DOX, 10 μM ROCKi, and 500 nM RO4929097 (Selleckchem, γ-Secretase inhibitor to inhibit NOTCH signaling). Cells were replated on PLO-Matrigel-coated plates as in the study by Manos et al. For imaging, 41,000 cells/cm^2 were seeded in 0.45 mL/cm^2; for RNA analysis, 100,000 cells/cm^2 in 0.42 mL/cm^2. On day 3, 70% medium change was performed with NMM supplemented with 2 μg/mL DOX, 5 μM ROCKi, and 500 nM RO4929097. A 1 μg/mL mitomycin C (Merck) treatment was performed for 1 h at 37°C on day 6. Afterward, a complete medium change with NMM supplemented with 100 nM RO4929097 was performed. From then on, every 4–5 days, a 50% medium change with NMM was carried out.

Neural induction using BMWi

36,000 cells/cm^2 iPSCs were seeded on Matrigel-coated plates 1 day prior to neural induction, as described for iPSC culture. On day 0, cells were washed once with E6 medium (Thermo Fisher Scientific) followed by a complete medium change with 0.75 mL/cm^2 E6 medium with 500 nM LDN193189 (Merck, stock 10 mM), 1 μM IWP2 (Tocris, stock 5 mM), and 1 μM PD0325901 (Tocris, stock 10 mM) (BMWi treatment). The following day, a complete medium change with 1.25 mL/cm^2 N2B27 with BMWi treatment was performed. For the next 2 days (typically over the weekend), no medium change was required. Medium was replaced with fresh medium at day 4 with 1.25 mL/cm^2 N2B27 with BMWi. To remove the BMWi condition (day 4: sBMWi protocol; day 6: BMWi protocol), the predifferentiated progenitor cells were washed once with N2B27, and 1.25 mL/cm^2 N2B27 supplemented with 2 μg/mL DOX was added to stimulate the expression of NGN2. The following day, a complete medium change with the same medium was performed. To replate or cryopreserve the progenitor cells (sBMWi day 6; BMWi day 8), they were washed with DPBS (−/−) and incubated for 15–20 min with prewarmed Accutase with 10 μM ROCKi at 37°C. When the cells were completely detached, they were pipetted in a single-cell solution, diluted in 5× amount N2B27 medium, and collected by centrifugation at 300*g* for 5 min.

The replating was carried out as described earlier with the iNGN2 protocol. Two days after replating, a 70% medium change was carried out with NMM supplemented with 2 μg/mL DOX, 10 μM Y-27632, and 500 nM RO4929097. Five days after replating, the cells were incubated for 1 h with 1 μg/mL mitomycin C at 37°C and followed by a complete medium change with NMM with 100 nM RO. Afterward, a 50% medium change was performed every 4–5 days.

Regional patterning and differentiation of mDAN, MN, and DRG-like neurons

To pattern the cells along the dorsoventral axis during the BMWi treatment, CHIR99021 (CHIR, Tocris) or smoothened agonist (SAG, Merck) in different concentrations was added during the days 0–4 of the BMWi induction paradigm. NMM+ and NMM- were used to check patterning along the anterior-posterior axis (Table S5). For mDAN patterning, 500 nM SAG was added to sBMWi treatment. Additionally, on day 0, 0.75 μM CHIR, and on day 2–4, 1.5 μM CHIR was added, and NPCs were replated in the NMM- medium with supplements added as above. After 1 week, the medium was changed to NMM+. To pattern sensory neurons from day 0 to day 4, 500 nM LDN193189, 1 μM PD0325901, 1 μM RA (Merck), and 3 μM CHIR were added, and the cells were replated in the NMM+ medium with supplements added as above. The BMWi protocol was modified as follows to pattern

MNs: from day 0–6, 1 μM RA, and from day 1–6, 250 nM SAG was added.

Microscopy

For fixation, 8% formaldehyde solution (VWR) in DPBS (+/+) was added to the culture medium, and the plate was incubated for 15 min at room temperature. Cells were washed three times with DPBS (+/+). Block/perm solution (10% FCS [Sigma], 1% BSA [Sigma], and 0.2% Triton X-100 (Sigma) in DPBS [+/+]) was added. The plates were incubated for 60 min at 4°C.

Afterward, antibody solution (DPBS [+/+], 0.1% BSA) supplemented with the desired primary antibodies (Table S6) was applied. The plates were incubated overnight at 4°C. For the secondary antibody solution, the secondary antibodies complementary to the primary antibodies were diluted 1:1,000 (from donkey or goat, Thermo Fisher Scientific) with 1:2,000 HOECHST 33342 (HOECHST, Thermo Fisher Scientific) in the antibody solution. The cells were incubated 1 h at room temperature with the secondary antibody solution. IF pictures were carried out at Operetta (PerkinElmer). All compared images were taken with the same instrument settings. The quantitative image analysis was carried out with the Harmony HCS software. For this purpose, dead cells were identified based on the morphology of the nuclei and excluded from the analysis. As controls for all experiments, hiPSCs were stained the same way, and their expression was compared with that of neurons/NPCs, and the intensity of the positive cells was selected accordingly. A secondary antibody staining was used as a background control. For very dense cultures, Harmony could not be used as analysis software. In this case, the data were quantified using ImageJ with the same controls.

Real-time qPCR

Cells were washed once with DPBS (−/−) and lysed directly on the plate in RLT Plus buffer (QIAGEN), and RNA was extracted using the RNeasy Plus Mini (QIAGEN) Kit and reversely transcribed using SuperScript IV VILO reverse transcriptase (Thermo Fisher Scientific), analyzed using TaqMan Assays (Thermo Fisher Scientific, Table S7) in a 20 μL reaction with 10 ng RNA input against the genes of interest, processed on a QuantStudio 7 Real-Time PCR System (Thermo Fisher Scientific), normalized to 3 housekeeping genes (*GAPDH*, *PPIA*, and *RPL13*), and calculated using the 2-ΔΔCt method using the hiPSC or iNGN2 differentiated condition of each experiment as a calibrator. The real-time qPCR (QuantStudio 7 Flex Real-Time PCR System, Thermo Fisher Scientific) was carried out under fast conditions. Initiation was set to 95°C for 20 s, followed by 40 cycles with denaturation at 95°C for 1 s and annealing/extension for 20 s at 60°C. The real-time qPCR results were evaluated with GraphPad Prism 8. The CT (cycle threshold) values of the samples were normalized to the CT values of *GAPDH*, *RPL13*, and *PPIA*. Then, the fold change was calculated against a respective relevant variable. If the CT values were undetected, the CT value was set arbitrarily to the maximum cycle number (CT = 40). The statistical analyses were carried out with GraphPad Prism 10. Prior to the statistical analysis, the groups to be examined were checked for their normal distribution using the Shapiro-Wilk test. To compare two groups, an unpaired t test was performed for equal variances and a Welch's test for different variances. The significant differences in one-way ANOVA (line in graph) or t-test (downward-reaching bracket in graph) were shown in all graphs as follows: *$p < 0.05$, **$p < 0.01$, ***$p < 0.001$.

MEA

The MEA was performed with Maestro Pro (Axion Biosystems). 30,000 cells/well were replated via drop seeding on 48 well poly(ethyleneimine) solution (PEI 50%, Merck) precoated MEA plates. For PEI coating, 5 μL freshly prepared 0.1% PEI (1 mL 10% PEI solution [2 g 50% PEI stock solution, 8 mL sterile water] with 99 mL 25 mM borate buffer pH 8.4 [Merck]) was dropped in the middle of the well and incubated at 37°C for 1 h. Afterward, the wells were washed two times with sterile water, two times with DPBS (−/−), and dried out overnight at room temperature. Cells were seeded as a drop in the middle of the well in MEA replate medium (7:1 NMM supplemented with 2 μg/mL DOX, 10 μM ROCKi, and 500 nM RO4929097: 193 μg/mL Matrigel in DEMEM/F12). After 1 h at 37°C, the well was filled up with NMM supplemented with 2 μg/mL DOX, 10 μM ROCKi, and 500 nM RO4929097. The cells were then further treated as described in the protocols earlier. To determine the activity of the BMi DRG, a baseline was first recorded for 30 min, then CAP (Tocris) dissolved in NMM/DMSO was added, and activity was measured for further 60 min. Only NMM/DMSO was added as control. For analysis, AxIS Navigator, AxIS Metric Plotting Tool, Neural Metric Tool, and Axion Data Export Tool (all Axion Biosystems) were used.

Bulk RNA-seq

For RNA-seq, the Illumina NextSeq 550 System with NextSeq 500/550 High Output Kit v.2.5 (Illumina, 20024906) was used. Library preparation was performed with Illumina Stranded mRNA Prep, Ligation (20040534) and IDT for Illumina RNA UD Indexes Set A, B, C (20040553, 20040554, 20040555). The denaturation was carried out according to protocol A (Denature and Dilute Libraries Guide, Illumina). Sequencing quality control was performed with FastQC (version 0.11.9) (Andrews, 2010) and MultiQC (version 1.9) (Ewels et al., 2016)

software. Alignment was conducted with STAR (version 2.7.1a) (Dobin et al., 2013) against the Homo sapiens genome assembly GRCh38 (gencode v.31). FeatureCounts (v.1.6.5) (Liao et al., 2014) was used to assign aligned reads to genes. Genes lowly expressed across conditions were filtered out, retaining those expressed at $\geq$ 1 CPM (counts per million) in at least four samples. Raw counts were log transformed and trimmed mean of M values normalized (Robinson and Oshlack, 2010). Differential gene expression was assessed by using limma-voom (v.3.5) (Law et al., 2014; Ritchie et al., 2015). Principal component analysis was performed to assess the data distribution across samples. Heatmaps show top 500 most variable genes, unless indicated otherwise. A direct comparison between different sample sets (fresh and frozen samples, BMWi and dSMAD protocols, different BMWi protocol lengths) was assessed by Pearson correlation of logarithmized, normalized expression levels. Differential gene expression was visualized using the R package EnhancedVolcano (v.1.20.0) (Blighe et al., 2024) with a fold change threshold of 1.5 and an adjusted p value cutoff of 1e−05.

Brain region analysis

The data used for the brain region analysis were obtained from the GTEx Portal on 01/04/2023 as median gene-level TPMs (transcripts per million) by tissues (v.8, RNA-SeQCv1.1.9) (Lonsdale et al., 2013). For selected brain region-specific tissues and spinal cord tissue, Spearman correlation coefficients were assessed between mean expression levels per sample group of BMWi/dSMAD protocol data and GTEx expression. The R package corrplot (v.0.92) (Wei and Simko, 2021) was used to visualize results with both circle size and color intensity encoding correlation coefficients.

snRNA-seq

For single-nuclei isolation, cells were washed with cold DPBS (−/−). Each well was treated with 500 μL of lysis buffer (Table S8) on ice, and samples were incubated for 15 min. Cells were scraped off, and the lysate was mixed with 1.4 mL washing buffer (Table S9). After 10-min centrifugation step at 4°C and 500xg, the cells were subjected to a second wash with 800 μL of washing buffer. Subsequently, cells were resuspended in washing buffer containing 1:750 Sytox blue solution (Thermo Fisher Scientific). 65,000 nuclei (SytoxBlue+) were sorted on a BD FACSAria Fusion flow Cvtometer into 1.5 mL DNA LoBind tubes (Eppendorf) pre-coated with 20% BSA (Merck) in PBS pH 7.2 (Thermo Fisher Scientific). snRNA-seq procedure was performed with the Chromium Next GEM Single Cell 3′ Reagent Kit v.3.1 (10× Genomics) for generating Gel Beads-in-emulsion (GEMs), barcoding and cDNA amplification as well as the 3′ gene expression library according to the manufacturer's protocol for Chromium Next GEM Single Cell 3′ Dual Index Reagent Kits v.3.1. Sorted nuclei suspensions were mixed with master mix (reverse transcriptase and template switch oligo) and loaded with Next GEM Single Cell 3′ v.3.1 Gel Beads and the Chromium Partitioning Oil in different wells of a Next GEM Chip G. After completion of the GEM generation on the Chromium X instrument, GEMs were transferred and incubated in a SimpliAmp Thermal Cycler for generating cDNA. After cleanup with Recovery Agent and Dynabeads MyOne SILANE followed by a double washing step with final 80% ethanol (Merck) in nuclease-free water (Thermo Fisher Scientific), cDNA was amplified with 13 PCR cycles and cleaned up with SPRIselect Reagent Kit (Beckman Coulter). cDNA QC (quality control) and quantification were carried out with the High Sensitivity D5000 ScreenTape assay (Agilent) on a 4200 TapeStation. 25% of generated cDNA underwent enzymatic fragmentation, end repair, and A-tailing, followed by double-sided size selection SPRIselect cleanup, before adapter ligation. After post ligation cleanup with SPRIselect, sample indices are added by 13 PCR cycles, and double-sided size selection purified final library for QC (average library fragment sizes) and quantification with D1000 ScreenTape assay (Agilent) on 4200 TapeStation. In addition, the library concentrations were measured with Qubit dsDNA High Sensitivity assay (Thermo Fisher Scientific) on Qubit Flex Fluorometer to confirm the measured concentrations. Libraries were normalized first to 10 nM, then to 1 nM, and pooled and denatured using 0.2 NaOH (Sigma-Aldrich) and 200 mM Tris-HCl (Thermo Fisher Scientific). Finally, 1.8 pM of pooled and denatured library was sequenced on a NextSeq 550 (Illumina). Next-generation sequencing data were preprocessed using cellranger 7.2.0 with 10× Genomics "GRCh38-2020-A" pre-mRNA reference. Filtering and QC were done using the Seurat package (v.5.1.0) (Hao et al., 2024). Nuclei were quality checked, and gene expression matrices were merged and filtered to have >200 features and features that were detected in at least 3 cells. Additional filtering was applied to remove potential outliers or low-quality cells by filtering for mitochondrial gene percentages <10%, number of features >400 and <6,000, and for UMI counts >1,000 and <2,0000. Doublets were removed using the R package scDblFinder 2. Samples were transformed using SCTransform function with the regression variables, UMI counts, and mitochondrial gene percentages (Germain et al., 2021). The principal components were calculated using the first 2,000 variable genes, and the uniform manifold approximation and projection (UMAP) dimensionality reduction was performed with the top 30 principal components. The Leiden clustering was done using a resolution of 0.4 resulting in 13 clusters (Hafemeister and Satija, 2019). Data visualization was

achieved using functions from the Seurat and scCustomize packages (Marsh, 2021).

Tau seeding assay

For tau seeding, cells were treated according to the appropriate protocol. On day 14 after replating, the neurons (hiPSC_3/8) were treated with 18.75–150 nM (calculated based on the molecular weight of full-length Tau monomer (2N4R)) recombinant paired helical filaments. A 50% medium change with NMM+ was performed 2 days post seed treatment, followed by regular 50% medium changes twice a week. 3 or 4 weeks after seeding, cells were fixed with MeOH: telencephalic neurons were washed once with DPBS (−/−), fixed with 100% ice-cold methanol at −20°C for 15 min, and subsequently washed 3 times with DPBS (−/−). The plates were incubated with blocking solution (3% BSA (Sigma) in DPBS (−/−)) for 1 h at room temperature. Afterward, they were stained as described in microscopy selection. Statistical analysis for this assay was performed using GraphPad Prism 10. A two-way ANOVA was performed with replicates and protocol or sPHF concentration as two variants. Significant differences were shown as follows: *$p < 0.05$, **$p < 0.01$, ***$p < 0.001$.

Statistical analysis

The procedure described here was used to evaluate data from IF quantification, real-time qPCR, and bulk RNA-seq data. Statistical analyses were carried out with GraphPad Prism 10. Prior to the statistical analysis, the groups to be examined were checked for their normal distribution using the Shapiro-Wilk test. To compare two groups, an unpaired t test was performed for equal variances and a Welch's test for different variances. The significant differences in one-way ANOVA (line in the graph) or t test (downward-reaching bracket in graph) were shown in all graphs as follows: *$p < 0.05$, **$p < 0.01$, ***$p < 0.001$.

RESOURCE AVAILABILITY

Lead contact

Further information and requests for resources and sequencing data should be directed to the lead contact, Peter Reinhardt (peter.reinhardt@abbvie.com).

Materials availability

This study did not generate new unique reagents.

Data and code availability

The data that support the findings of this study are openly available via GEO (https://www.ncbi.nlm.nih.gov/geo/), under the accession nos. GEO: GSE253508, GEO: GSE253509, GEO: GSE279593, GSE279590 and GEO: GSE279591.

ACKNOWLEDGMENTS

We would like to thank AbbVie employees Elke Käfer and Anja Fink for their technical assistance to provide frozen stocks of hiPSCs. We would also like to thank Prof. Dr. Martin Grininger and Prof. Dr. Jasmin Hefendehl for their supervision. A.K. was funded by the Graduiertenkolleg TASCDT of the MWK Baden-Württemberg and the Albert und Anneliese Konanz-Stiftung. Principles of this study are based on a doctoral thesis, which was submitted and accepted to fulfill in part the requirements for the degree of a doctor sc. hum at University of Heidelberg, Germany. Data in Figure S3 are also part of this thesis. The following figures were created with BioRender.com: graphical abstract, Figures 1A, 2A, S5A/E, 5A, 6/D, S6A, and 7A. Thanks to the team of Peter Davies of Feinstein Institute for providing the MC1 antibody; this was received through a material transfer agreement.

AUTHOR CONTRIBUTIONS

Conceptualization, P.R., A.K., and C.H.; methodology, P.R., A.K., and C.H.; validation, C.H.; formal analysis, C.H.; investigation, C.H., A.K., M.J.H., M.W., N.N., V.H., D.G., and J.K.; data curation, C.H., A.W., M.P.K., and T.L.; writing – original draft, P.R. and C.H.; writing – review and editing, P.R., C.H., A.K., A.W., M.J.H., N.N., C. S., L.R., V.H., T.L., C.U., L.N.M., D.S., H.L., L.B., B.M.-S., R.W., J.R., I. W., R.R., M.H., J.D.M., M.P.K., M.W., and M.C.; visualization, C.H., P.R., A.K., M.P.K., and V.H.; supervision, P.R.; project administration, P.R.

DECLARATION OF INTERESTS

C.H., A.W., M.J.H., M.W., M.P.K., N.N., C.S., L.R., T.L., C.U., J.K., D. G., D.S., H.L., L.B., B.M.-S., M.S.B., R.W., J.R., J.D.M., M.C., and P.R. are employees of AbbVie. L.N.M., I.W., and V.H. were employees of AbbVie at the time of the study. R.R. and M.H. are current employees of Center for Mass Spectrometry and Optical Spectroscopy, Mannheim University of Applied Sciences and Institute of Medical Technology, Heidelberg University and Mannheim University of Applied Sciences. A.K. is a current employee for Struktur-und Genehmigungsdirektion Süd. The design, study conduct, and financial support for this research were provided by AbbVie. AbbVie participated in the interpretation of data, review, and approval of the publication.

SUPPLEMENTAL INFORMATION

Supplemental information can be found online at https://doi.org/10.1016/j.stemcr.2025.102539.

Received: January 18, 2024
Revised: May 21, 2025
Accepted: May 22, 2025
Published: June 19, 2025

REFERENCES

Andrews, S. (2010). FastQC: A Quality Control Tool for High Throughput Sequence Data (Babraham Bioinformatics, Babraham Institute).

Berry, C.T., Sceniak, M.P., Zhou, L., and Sabo, S.L. (2012). Developmental Up-Regulation of Vesicular Glutamate Transporter-1 Promotes Neocortical Presynaptic Terminal Development. PLoS One *7*, e50911. https://doi.org/10.1371/journal.pone.0050911.

Bertuzzi, S., Porter, F.D., Pitts, A., Kumar, M., Agulnick, A., Wassif, C., and Westphal, H. (1999). Characterization of Lhx9, a novel LIM/homeobox gene expressed by the pioneer neurons in the mouse cerebral cortex. Mech. Dev. *81*, 193–198. https://doi.org/10.1016/s0925-4773(98)00233-0.

Black, B.J., Atmaramani, R., Kumaraju, R., Plagens, S., Romero-Ortega, M., Dussor, G., Price, T.J., Campbell, Z.T., and Pancrazio, J.J. (2018). Adult mouse sensory neurons on microelectrode arrays exhibit increased spontaneous and stimulus-evoked activity in the presence of interleukin-6. J. Neurophysiol. *120*, 1374–1385. https://doi.org/10.1152/jn.00158.2018.

Blighe, K., Rana, S., and Lewis, M. (2024). EnhancedVolcano: Publication-ready volcano plots with enhanced colouring and labeling. R package version 1.22.0. https://github.com/kevinblighe/EnhancedVolcano.

Boulland, J.L., Qureshi, T., Seal, R.P., Rafiki, A., Gundersen, V., Bergersen, L.H., Fremeau, R.T., Edwards, R.H., Storm-Mathisen, J., and Chaudhry, F.A. (2004). Expression of the vesicular glutamate transporters during development indicates the widespread corelease of multiple neurotransmitters. J. Comp. Neurol. *480*, 264–280. https://doi.org/10.1002/cne.20354.

Calder, E.L., Tchieu, J., Steinbeck, J.A., Tu, E., Keros, S., Ying, S.-W., Jaiswal, M.K., Cornacchia, D., Goldstein, P.A., Tabar, V., and Studer, L. (2015). Retinoic Acid-Mediated Regulation of GLI3 Enables Efficient Motoneuron Derivation from Human ESCs in the Absence of Extrinsic SHH Activation. J. Neurosci. *35*, 11462–11481. https://doi.org/10.1523/jneurosci.3046-14.2015.

Campos-Peña, V., and Meraz-Ríos, M.A. (2014). Neurochemistry. https://doi.org/10.5772/57367.

Carney, T.J., Dutton, K.A., Greenhill, E., Delfino-Machín, M., Dufourcq, P., Blader, P., and Kelsh, R.N. (2006). A direct role for Sox10 in specification of neural crest-derived sensory neurons. Development *133*, 4619–4630. https://doi.org/10.1242/dev.02668.

Chambers, S.M., Fasano, C.A., Papapetrou, E.P., Tomishima, M., Sadelain, M., and Studer, L. (2009). Highly efficient neural conversion of human ES and iPS cells by dual inhibition of SMAD signaling. Nat. Biotechnol. *27*, 275–280. https://doi.org/10.1038/nbt.1529.

Chambers, S.M., Qi, Y., Mica, Y., Lee, G., Zhang, X.-J., Niu, L., Bilsland, J., Cao, L., Stevens, E., Whiting, P., et al. (2012). Combined small-molecule inhibition accelerates developmental timing and converts human pluripotent stem cells into nociceptors. Nat. Biotechnol. *30*, 715–720. https://doi.org/10.1038/nbt.2249.

Chen, B., Dodge, M.E., Tang, W., Lu, J., Ma, Z., Fan, C.-W., Wei, S., Hao, W., Kilgore, J., Williams, N.S., et al. (2009). Small molecule–mediated disruption of Wnt-dependent signaling in tissue regeneration and cancer. Nat. Chem. Biol. *5*, 100–107. https://doi.org/10.1038/nchembio.137.

Chen, M., Maimaitili, M., Habekost, M., Gill, K.P., Mermet-Joret, N., Nabavi, S., Febbraro, F., and Denham, M. (2020). Rapid generation of regionally specified CNS neurons by sequential patterning and conversion of human induced pluripotent stem cells. Stem Cell Res. *48*, 101945. https://doi.org/10.1016/j.scr.2020.101945.

Cohen, M.A., Itsykson, P., and Reubinoff, B.E. (2010). The role of FGF-signaling in early neural specification of human embryonic stem cells. Dev. Biol. *340*, 450–458. https://doi.org/10.1016/j.ydbio.2010.01.030.

Cossart, R., Aronov, D., and Yuste, R. (2003). Attractor dynamics of network UP states in the neocortex. Nature *423*, 283–288. https://doi.org/10.1038/nature01614.

Dasen, J.S., Tice, B.C., Brenner-Morton, S., and Jessell, T.M. (2005). A Hox Regulatory Network Establishes Motor Neuron Pool Identity and Target-Muscle Connectivity. Cell *123*, 477–491. https://doi.org/10.1016/j.cell.2005.09.009.

Dobin, A., Davis, C.A., Schlesinger, F., Drenkow, J., Zaleski, C., Jha, S., Batut, P., Chaisson, M., and Gingeras, T.R. (2013). STAR: ultrafast universal RNA-seq aligner. Bioinformatics *29*, 15–21. https://doi.org/10.1093/bioinformatics/bts635.

Du, Z.-W., Chen, H., Liu, H., Lu, J., Qian, K., Huang, C.-L., Zhong, X., Fan, F., and Zhang, S.-C. (2015). Generation and expansion of highly pure motor neuron progenitors from human pluripotent stem cells. Nat. Commun. *6*, 6626. https://doi.org/10.1038/ncomms7626.

Durston, A.J., Timmermans, J.P., Hage, W.J., Hendriks, H.F., de Vries, N.J., Heideveld, M., and Nieuwkoop, P.D. (1989). Retinoic acid causes an anteroposterior transformation in the developing central nervous system. Nature *340*, 140–144. https://doi.org/10.1038/340140a0.

Eaton, M., Que, Z., Zhang, J., Beck, K., Shi, R., McDermott, J., Ladisch, M., and Yang, Y. (2021). Multi-Electrode Array of Sensory Neurons as an In Vitro Platform to Identify the Nociceptive Response to Pharmaceutical Buffer Systems of Injectable Biologics. Pharm. Res. *38*, 1179–1186. https://doi.org/10.1007/s11095-021-03075-z.

Ewels, P., Magnusson, M., Lundin, S., and Käller, M. (2016). MultiQC: summarize analysis results for multiple tools and samples in a single report. Bioinformatics *32*, 3047–3048. https://doi.org/10.1093/bioinformatics/btw354.

Fedele, S., Collo, G., Behr, K., Bischofberger, J., Müller, S., Kunath, T., Christensen, K., Gündner, A.L., Graf, M., Jagasia, R., and Taylor, V. (2017). Expansion of human midbrain floor plate progenitors from induced pluripotent stem cells increases dopaminergic neuron differentiation potential. Sci. Rep. *7*, 6036. https://doi.org/10.1038/s41598-017-05633-1.

Fiock, K.L., Smalley, M.E., Crary, J.F., Pasca, A.M., and Hefti, M.M. (2020). Increased Tau Expression Correlates with Neuronal Maturation in the Developing Human Cerebral Cortex. ENeuro *7*, ENEURO.0058-20.2020. https://doi.org/10.1523/eneuro.0058-20.2020.

Garcini, E.M. de, Serrano, L., and Avila, J. (1986). Self assembly of microtubule associated protein tau into filaments resembling those found in alzheimer disease. Biochem. Biophys. Res. Commun. *141*, 790–796. https://doi.org/10.1016/s0006-291x(86)80242-x.

Germain, P.-L., Lun, A., Garcia Meixide, C., Macnair, W., and Robinson, M.D. (2021). Doublet identification in single-cell

sequencing data using scDblFinder. F1000Res. *10*, 979. https://doi.org/10.12688/f1000research.73600.2.

Greber, B., Coulon, P., Zhang, M., Moritz, S., Frank, S., Müller-Molina, A.J., Araúzo-Bravo, M.J., Han, D.W., Pape, H.C., and Schöler, H.R. (2011). FGF signalling inhibits neural induction in human embryonic stem cells. EMBO J. *30*, 4874–4884. https://doi.org/10.1038/emboj.2011.407.

Grundke-Iqbal, I., Iqbal, K., Quinlan, M., Tung, Y.C., Zaidi, M.S., and Wisniewski, H.M. (1986). Microtubule-associated protein tau. A component of Alzheimer paired helical filaments. J. Biol. Chem. *261*, 6084–6089. https://doi.org/10.1016/s0021-9258(17)38495-8.

Hafemeister, C., and Satija, R. (2019). Normalization and variance stabilization of single-cell RNA-seq data using regularized negative binomial regression. Genome Biol. *20*, 296. https://doi.org/10.1186/s13059-019-1874-1.

Hao, Y., Stuart, T., Kowalski, M.H., Choudhary, S., Hoffman, P., Hartman, A., Srivastava, A., Molla, G., Madad, S., Fernandez-Granda, C., and Satija, R. (2024). Dictionary learning for integrative, multimodal and scalable single-cell analysis. Nat. Biotechnol. *42*, 293–304. https://doi.org/10.1038/s41587-023-01767-y.

Hiller, B.M., Marmion, D.J., Gross, R.M., Thompson, C.A., Chavez, C.A., Brundin, P., Wakeman, D.R., McMahon, C.W., and Kordower, J.H. (2021). Mitomycin-C treatment during differentiation of induced pluripotent stem cell-derived dopamine neurons reduces proliferation without compromising survival or function in vivo. Stem Cells Transl. Med. *10*, 278–290. https://doi.org/10.1002/sctm.20-0014.

Hof, P.R., Perl, D.P., Loerzel, A.J., and Morrison, J.H. (1991). Neurofibrillary tangle distribution in the cerebral cortex of parkinsonism-dementia cases from Guam: differences with Alzheimer's disease. Brain Res. *564*, 306–313. https://doi.org/10.1016/0006-8993(91)91467-f.

Hong, M., Zhukareva, V., Vogelsberg-Ragaglia, V., Wszolek, Z., Reed, L., Miller, B.I., Geschwind, D.H., Bird, T.D., McKeel, D., Goate, A., et al. (1998). Mutation-Specific Functional Impairments in Distinct Tau Isoforms of Hereditary FTDP-17. Sci. Technol. Humanit. *282*, 1914–1917. https://doi.org/10.1126/science.282.5395.1914.

Johns, A.E., and Maragakis, N.J. (2022). Exploring Motor Neuron Diseases Using iPSC Platforms. Stem Cell. *40*, 2–13. https://doi.org/10.1093/stmcls/sxab006.

Kamioka, H., Maeda, E., Jimbo, Y., Robinson, H.P., and Kawana, A. (1996). Spontaneous periodic synchronized bursting during formation of mature patterns of connections in cortical cultures. Neurosci. Lett. *206*, 109–112. https://doi.org/10.1016/s0304-3940(96)12448-4.

Karner, C.M., Merkel, C.E., Dodge, M., Ma, Z., Lu, J., Chen, C., Lum, L., and Carroll, T.J. (2010). Tankyrase is necessary for canonical Wnt signaling during kidney development. Dev. Dyn. *239*, 2014–2023. https://doi.org/10.1002/dvdy.22340.

Karran, E., and De Strooper, B. (2022). The amyloid hypothesis in Alzheimer disease: new insights from new therapeutics. Nat. Rev. Drug Discov. *21*, 306–318. https://doi.org/10.1038/s41573-022-00391-w.

KIDD, M. (1963). Paired Helical Filaments in Electron Microscopy of Alzheimer's Disease. Nature *197*, 192–193. https://doi.org/10.1038/197192b0.

Kim, D.W., Washington, P.W., Wang, Z.Q., Lin, S.H., Sun, C., Ismail, B.T., Wang, H., Jiang, L., and Blackshaw, S. (2020). The cellular and molecular landscape of hypothalamic patterning and differentiation from embryonic to late postnatal development. Nat. Commun. *11*, 4360. https://doi.org/10.1038/s41467-020-18231-z.

Kim, D.W., Liu, K., Wang, Z.Q., Zhang, Y.S., Bathini, A., Brown, M.P., Lin, S.H., Washington, P.W., Sun, C., Lindtner, S., et al. (2021). Gene regulatory networks controlling differentiation, survival, and diversification of hypothalamic Lhx6-expressing GABAergic neurons. Commun. Biol. *4*, 95. https://doi.org/10.1038/s42003-020-01616-7.

Kim, D.W., Place, E., Chinnaiya, K., Manning, E., Sun, C., Dai, W., Groves, I., Ohyama, K., Burbridge, S., Placzek, M., and Blackshaw, S. (2022). Single-cell analysis of early chick hypothalamic development reveals that hypothalamic cells are induced from prethalamic-like progenitors. Cell Rep. *38*, 110251. https://doi.org/10.1016/j.celrep.2021.110251.

Kim, S.-K., Seo, S., Stein-O'Brien, G., Jaishankar, A., Ogawa, K., Micali, N., Luria, V., Karger, A., Wang, Y., Kim, H., et al. (2024). Individual variation in the emergence of anterior-to-posterior neural fates from human pluripotent stem cells. Stem Cell Rep. *19*, 1336–1350. https://doi.org/10.1016/j.stemcr.2024.07.004.

Kirkeby, A., Grealish, S., Wolf, D.A., Nelander, J., Wood, J., Lundblad, M., Lindvall, O., and Parmar, M. (2012). Generation of Regionally Specified Neural Progenitors and Functional Neurons from Human Embryonic Stem Cells under Defined Conditions. Cell Rep. *1*, 703–714. https://doi.org/10.1016/j.celrep.2012.04.009.

Kreuser, U., Buchert, J., Haase, A., Richter, W., and Diederichs, S. (2020). Initial WNT/β-Catenin Activation Enhanced Mesoderm Commitment, Extracellular Matrix Expression, Cell Aggregation and Cartilage Tissue Yield From Induced Pluripotent Stem Cells. Front. Cell Dev. Biol. *8*, 581331. https://doi.org/10.3389/fcell.2020.581331.

Law, C.W., Chen, Y., Shi, W., and Smyth, G.K. (2014). voom: precision weights unlock linear model analysis tools for RNA-seq read counts. Genome Biol. *15*, R29. https://doi.org/10.1186/gb-2014-15-2-r29.

Lek, M., Dias, J.M., Marklund, U., Uhde, C.W., Kurdija, S., Lei, Q., Sussel, L., Rubenstein, J.L., Matise, M.P., Arnold, H.-H., et al. (2010). A homeodomain feedback circuit underlies step-function interpretation of a Shh morphogen gradient during ventral neural patterning. Development *137*, 4051–4060. https://doi.org/10.1242/dev.054288.

Liao, Y., Smyth, G.K., and Shi, W. (2014). featureCounts: an efficient general purpose program for assigning sequence reads to genomic features. Bioinformatics *30*, 923–930. https://doi.org/10.1093/bioinformatics/btt656.

Limone, F., Guerra San Juan, I., Mitchell, J.M., Smith, J.L.M., Raghunathan, K., Meyer, D., Ghosh, S.D., Couto, A., Klim, J.R., Joseph, B.J., et al. (2023). Efficient generation of lower induced motor neurons by coupling Ngn2 expression with developmental cues.

Cell Rep. *42*, 111896. https://doi.org/10.1016/j.celrep.2022.111896.

Lin, H.-C., He, Z., Ebert, S., Schörnig, M., Santel, M., Nikolova, M. T., Weigert, A., Hevers, W., Kasri, N.N., Taverna, E., et al. (2021). NGN2 induces diverse neuron types from human pluripotency. Stem Cell Rep. *16*, 2118–2127. https://doi.org/10.1016/j.stemcr.2021.07.006.

Liu, F., and Gong, C.-X. (2008). Tau exon 10 alternative splicing and tauopathies. Mol. Neurodegener. *3*, 8. https://doi.org/10.1186/1750-1326-3-8.

Liu, J., Chang, L., Song, Y., Li, H., and Wu, Y. (2019). The Role of NMDA Receptors in Alzheimer's Disease. Front. Neurosci. *13*, 43. https://doi.org/10.3389/fnins.2019.00043.

Lonsdale, J., Thomas, J., Salvatore, M., Phillips, R., Lo, E., Shad, S., Hasz, R., Walters, G., Garcia, F., Young, N., et al. (2013). The Genotype-Tissue Expression (GTEx) project. Nat. Genet. *45*, 580–585. https://doi.org/10.1038/ng.2653.

Manos, J.D., Preiss, C.N., Venkat, N., Tamm, J., Reinhardt, P., Kwon, T., Wu, J., Winter, A.D., Jahn, T.R., Yanamandra, K., et al. (2022). Uncovering specificity of endogenous TAU aggregation in a human iPSC-neuron TAU seeding model. iScience *25*, 103658. https://doi.org/10.1016/j.isci.2021.103658.

Maroof, A.M., Keros, S., Tyson, J.A., Ying, S.-W., Ganat, Y.M., Merkle, F.T., Liu, B., Goulburn, A., Stanley, E.G., Elefanty, A.G., et al. (2013). Directed Differentiation and Functional Maturation of Cortical Interneurons from Human Embryonic Stem Cells. Cell Stem Cell *12*, 559–572. https://doi.org/10.1016/j.stem.2013.04.008.

Marsh, S. (2021). scCustomize: Custom Visualizations & Functions for Streamlined Analyses of Single Cell Sequencing. https://doi.org/10.5281/zenodo.5706430.RRID:SCR_024675.

Mertens, J., Herdy, J.R., Traxler, L., Schafer, S.T., Schlachetzki, J.C. M., Böhnke, L., Reid, D.A., Lee, H., Zangwill, D., Fernandes, D.P., et al. (2021). Age-dependent instability of mature neuronal fate in induced neurons from Alzheimer's patients. Cell Stem Cell *28*, 1533–1548.e6. https://doi.org/10.1016/j.stem.2021.04.004.

Moya, N., Cutts, J., Gaasterland, T., Willert, K., and Brafman, D.A. (2014). Endogenous WNT Signaling Regulates hPSC-Derived Neural Progenitor Cell Heterogeneity and Specifies Their Regional Identity. Stem Cell Rep. *3*, 1015–1028. https://doi.org/10.1016/j.stemcr.2014.10.004.

Münst, S., Koch, P., Kesavan, J., Alexander-Mays, M., Münst, B., Blaess, S., and Brüstle, O. (2018). In vitro segregation and isolation of human pluripotent stem cell-derived neural crest cells. Methods *133*, 65–80. https://doi.org/10.1016/j.ymeth.2017.09.012.

Nehme, R., Zuccaro, E., Ghosh, S.D., Li, C., Sherwood, J.L., Pietilainen, O., Barrett, L.E., Limone, F., Worringer, K.A., Kommineni, S., et al. (2018). Combining NGN2 Programming with Developmental Patterning Generates Human Excitatory Neurons with NMDAR-Mediated Synaptic Transmission. Cell Rep. *23*, 2509–2523. https://doi.org/10.1016/j.celrep.2018.04.066.

Neyrinck, K., Van Den Daele, J., Vervliet, T., De Smedt, J., Wierda, K., Nijs, M., Vanbokhoven, T., D'hondt, A., Planque, M., Fendt, S.-M., et al. (2021). SOX9-induced Generation of Functional Astrocytes Supporting Neuronal Maturation in an All-human System. Stem Cell Rev. Rep. *17*, 1855–1873. https://doi.org/10.1007/s12015-021-10179-x.

Pera, M.F., Andrade, J., Houssami, S., Reubinoff, B., Trounson, A., Stanley, E.G., Ward-van Oostwaard, D., and Mummery, C. (2004). Regulation of human embryonic stem cell differentiation by BMP-2 and its antagonist noggin. J. Cell Sci. *117*, 1269–1280. https://doi.org/10.1242/jcs.00970.

Qi, Y., Zhang, X.-J., Renier, N., Wu, Z., Atkin, T., Sun, Z., Ozair, M.Z., Tchieu, J., Zimmer, B., Fattahi, F., et al. (2017). Combined small-molecule inhibition accelerates the derivation of functional cortical neurons from human pluripotent stem cells. Nat. Biotechnol. *35*, 154–163. https://doi.org/10.1038/nbt.3777.

Qu, Y., Han, B., Gao, B., Bose, S., Gong, Y., Wawrowsky, K., Giuliano, A.E., Sareen, D., and Cui, X. (2017). Differentiation of Human Induced Pluripotent Stem Cells to Mammary-like Organoids. Stem Cell Rep. *8*, 205–215. https://doi.org/10.1016/j.stemcr.2016.12.023.

Rao, J., Pfeiffer, M.J., Frank, S., Adachi, K., Piccini, I., Quaranta, R., Araúzo-Bravo, M., Schwarz, J., Schade, D., Leidel, S., et al. (2016). Stepwise Clearance of Repressive Roadblocks Drives Cardiac Induction in Human ESCs. Cell Stem Cell *18*, 341–353. https://doi.org/10.1016/j.stem.2015.11.019.

Rétaux, S., Rogard, M., Bach, I., Failli, V., and Besson, M.-J. (1999). Lhx9: A Novel LIM-Homeodomain Gene Expressed in the Developing Forebrain. J. Neurosci. *19*, 783–793. https://doi.org/10.1523/jneurosci.19-02-00783.1999.

Ritchie, M.E., Phipson, B., Wu, D., Hu, Y., Law, C.W., Shi, W., and Smyth, G.K. (2015). limma powers differential expression analyses for RNA-sequencing and microarray studies. Nucleic Acids Res. *43*, e47. https://doi.org/10.1093/nar/gkv007.

Robinson, M.D., and Oshlack, A. (2010). A scaling normalization method for differential expression analysis of RNA-seq data. Genome Biol. *11*, R25. https://doi.org/10.1186/gb-2010-11-3-r25.

Saberi-Moghadam, S., Simi, A., Setareh, H., Mikhail, C., and Tafti, M. (2018). In vitro Cortical Network Firing is Homeostatically Regulated: A Model for Sleep Regulation. Sci. Rep. *8*, 6297. https://doi.org/10.1038/s41598-018-24339-6.

Sanchez-Vives, M.V., and McCormick, D.A. (2000). Cellular and network mechanisms of rhythmic recurrent activity in neocortex. Nat. Neurosci. *3*, 1027–1034. https://doi.org/10.1038/79848.

Schoch, K.M., DeVos, S.L., Miller, R.L., Chun, S.J., Norrbom, M., Wozniak, D.F., Dawson, H.N., Bennett, C.F., Rigo, F., and Miller, T.M. (2016). Increased 4R-Tau Induces Pathological Changes in a Human-Tau Mouse Model. Neuron (Camb., Mass.) *90*, 941–947. https://doi.org/10.1016/j.neuron.2016.04.042.

Schöndorf, D.C., Elschami, M., Schieck, M., Ercan-Herbst, E., Weber, C., Riesinger, Y., Kalman, S., Steinemann, D., and Ehrnhoefer, D.E. (2019). Generation of an induced pluripotent stem cell cohort suitable to investigate sporadic Alzheimer's Disease. Stem Cell Res. *34*, 101351. https://doi.org/10.1016/j.scr.2018.11.012.

Seubert, P., Mawal-Dewan, M., Barbour, R., Jakes, R., Goedert, M., Johnson, G.V., Litersky, J.M., Schenk, D., Lieberburg, I., Trojanowski, J.Q., et al. (1995). Detection of Phosphorylated Ser262 in Fetal Tau, Adult Tau, and Paired Helical Filament Tau (*). J. Biol. Chem. *270*, 18917–18922. https://doi.org/10.1074/jbc.270.32.18917.

Sheta, R., Teixeira, M., Idi, W., Pierre, M., de Rus Jacquet, A., Emond, V., Zorca, C.E., Vanderperre, B., Durcan, T.M., Fon, E.A., et al. (2022). Combining NGN2 programming and dopaminergic patterning for a rapid and efficient generation of hiPSC-derived midbrain neurons. Sci. Rep. *12*, 17176. https://doi.org/10.1038/s41598-022-22158-4.

Shi, Y., Kirwan, P., and Livesey, F.J. (2012). Directed differentiation of human pluripotent stem cells to cerebral cortex neurons and neural networks. Nat. Protoc. *7*, 1836–1846. https://doi.org/10.1038/nprot.2012.116.

Shih, P.-Y., Kreir, M., Kumar, D., Seibt, F., Pestana, F., Schmid, B., Holst, B., Clausen, C., Steeg, R., Fischer, B., et al. (2021). Development of a fully human assay combining NGN2-inducible neurons co-cultured with iPSC-derived astrocytes amenable for electrophysiological studies. Stem Cell Res. *54*, 102386. https://doi.org/10.1016/j.scr.2021.102386.

Smith, J.R., Vallier, L., Lupo, G., Alexander, M., Harris, W.A., and Pedersen, R.A. (2008). Inhibition of Activin/Nodal signaling promotes specification of human embryonic stem cells into neuroectoderm. Dev. Biol. *313*, 107–117. https://doi.org/10.1016/j.ydbio.2007.10.003.

Smits, L.M., Reinhardt, L., Reinhardt, P., Glatza, M., Monzel, A.S., Stanslowsky, N., Rosato-Siri, M.D., Zanon, A., Antony, P.M., Bellmann, J., et al. (2019). Modeling Parkinson's disease in midbrain-like organoids. NPJ Park.'s Dis. *5*, 5. https://doi.org/10.1038/s41531-019-0078-4.

Sterneckert, J.L., Reinhardt, P., and Schöler, H.R. (2014). Investigating human disease using stem cell models. Nat. Rev. Genet. *15*, 625–639. https://doi.org/10.1038/nrg3764.

Stolzenburg, L.R., Esmaeeli, S., Kulkarni, A.S., Murphy, E., Kwon, T., Preiss, C., Bahnassawy, L., Stender, J.D., Manos, J.D., Reinhardt, P., et al. (2023). Functional characterization of a single nucleotide polymorphism associated with Alzheimer's disease in a hiPSC-based neuron model. PLoS One *18*, e0291029. https://doi.org/10.1371/journal.pone.0291029.

Strano, A., Tuck, E., Stubbs, V.E., and Livesey, F.J. (2020). Variable Outcomes in Neural Differentiation of Human PSCs Arise from Intrinsic Differences in Developmental Signaling Pathways. Cell Rep. *31*, 107732. https://doi.org/10.1016/j.celrep.2020.107732.

Sun, W., Cornwell, A., Li, J., Peng, S., Osorio, M.J., Aalling, N., Wang, S., Benraiss, A., Lou, N., Goldman, S.A., and Nedergaard, M. (2017). SOX9 Is an Astrocyte-Specific Nuclear Marker in the Adult Brain Outside the Neurogenic Regions. J. Neurosci. *37*, 4493–4507. https://doi.org/10.1523/jneurosci.3199-16.2017.

Tao, Y., and Zhang, S.-C. (2016). Neural Subtype Specification from Human Pluripotent Stem Cells. Cell Stem Cell *19*, 573–586. https://doi.org/10.1016/j.stem.2016.10.015.

Tchieu, J., Zimmer, B., Fattahi, F., Amin, S., Zeltner, N., Chen, S., and Studer, L. (2017). A Modular Platform for Differentiation of Human PSCs into All Major Ectodermal Lineages. Cell Stem Cell *21*, 399–410.e7. https://doi.org/10.1016/j.stem.2017.08.015.

Togo, K., Fukusumi, H., Shofuda, T., Ohnishi, H., Yamazaki, H., Hayashi, M.K., Kawasaki, N., Takei, N., Nakazawa, T., Saito, Y., et al. (2021). Postsynaptic structure formation of human iPS cell-derived neurons takes longer than presynaptic formation during neural differentiation in vitro. Mol. Brain *14*, 149. https://doi.org/10.1186/s13041-021-00851-1.

Volpato, V., and Webber, C. (2020). Addressing variability in iPSC-derived models of human disease: guidelines to promote reproducibility. Dis. Model. Mech. *13*, dmm042317. https://doi.org/10.1242/dmm.042317.

Volpato, V., Smith, J., Sandor, C., Ried, J.S., Baud, A., Handel, A., Newey, S.E., Wessely, F., Attar, M., Whiteley, E., et al. (2018). Reproducibility of Molecular Phenotypes after Long-Term Differentiation to Human iPSC-Derived Neurons: A Multi-Site Omics Study. Stem Cell Rep. *11*, 897–911. https://doi.org/10.1016/j.stemcr.2018.08.013.

Walsh, P., Truong, V., Nayak, S., Saldías Montivero, M., Low, W.C., Parr, A.M., and Dutton, J.R. (2020). Accelerated differentiation of human pluripotent stem cells into neural lineages via an early intermediate ectoderm population. Stem Cell. *38*, 1400–1408. https://doi.org/10.1002/stem.3260.

Wang, C., Ward, M.E., Chen, R., Liu, K., Tracy, T.E., Chen, X., Xie, M., Sohn, P.D., Ludwig, C., Meyer-Franke, A., et al. (2017). Scalable Production of iPSC-Derived Human Neurons to Identify Tau-Lowering Compounds by High-Content Screening. Stem Cell Rep. *9*, 1221–1233. https://doi.org/10.1016/j.stemcr.2017.08.019.

Wei, T., and Simko, V. (2021). R package "corrplot": Visualization of a Correlation Matrix (Version 0.92). https://github.com/taiyun/corrplot.

Xu, P., He, H., Gao, Q., Zhou, Y., Wu, Z., Zhang, X., Sun, L., Hu, G., Guan, Q., You, Z., et al. (2022). Human midbrain dopaminergic neuronal differentiation markers predict cell therapy outcome in a Parkinson's disease model. J. Clin. Investig. *132*, e156768. https://doi.org/10.1172/jci156768.

Yang, F., and Zheng, J. (2017). Understand spiciness: mechanism of TRPV1 channel activation by capsaicin. Protein Cell *8*, 169–177. https://doi.org/10.1007/s13238-016-0353-7.

Young, G.T., Gutteridge, A., Fox, H.D., Wilbrey, A.L., Cao, L., Cho, L.T., Brown, A.R., Benn, C.L., Kammonen, L.R., Friedman, J.H., et al. (2014). Characterizing Human Stem Cell–derived Sensory Neurons at the Single-cell Level Reveals Their Ion Channel Expression and Utility in Pain Research. Mol. Ther. *22*, 1530–1543. https://doi.org/10.1038/mt.2014.86.

Zhang, X., Huang, C.T., Chen, J., Pankratz, M.T., Xi, J., Li, J., Yang, Y., LaVaute, T.M., Li, X.-J., Ayala, M., et al. (2010). Pax6 Is a Human Neuroectoderm Cell Fate Determinant. Cell Stem Cell *7*, 90–100. https://doi.org/10.1016/j.stem.2010.04.017.

Zhang, Y., Pak, C., Han, Y., Ahlenius, H., Zhang, Z., Chanda, S., Marro, S., Patzke, C., Acuna, C., Covy, J., et al. (2013). Rapid Single-Step Induction of Functional Neurons from Human Pluripotent Stem Cells. Neuron (Camb., Mass.) *78*, 785–798. https://doi.org/10.1016/j.neuron.2013.05.029.

Stem Cell Reports, Volume *20*

Supplemental Information

BMP, MEK, and WNT inhibition with NGN2 expression for rapid generation of hiPSC-derived neurons amenable to regional patterning

Carina Habich, Alexandra Kowalski, Astrid Wachter, Michaela J. Heimann, Michael Wolf, Markus P. Kummer, Nathalie Nicolaisen, Christopher Sliwinski, Lydia Reinhardt, Veronika Heil, Timo Lange, Christopher Untucht, Loan N. Miller, Jürgen Korffmann, Daniela Geist, David Schöndorf, Heyne Lee, Lamiaa Bahnassawy, Benjamin Mielich-Süss, Melanie S. Brennan, Ruven Wilkens, Julian Röwe, Ian Weidling, Rüdiger Rudolf, Mathias Hafner, Justine D. Manos, Miroslav Cik, and Peter Reinhardt

Supplementary Information

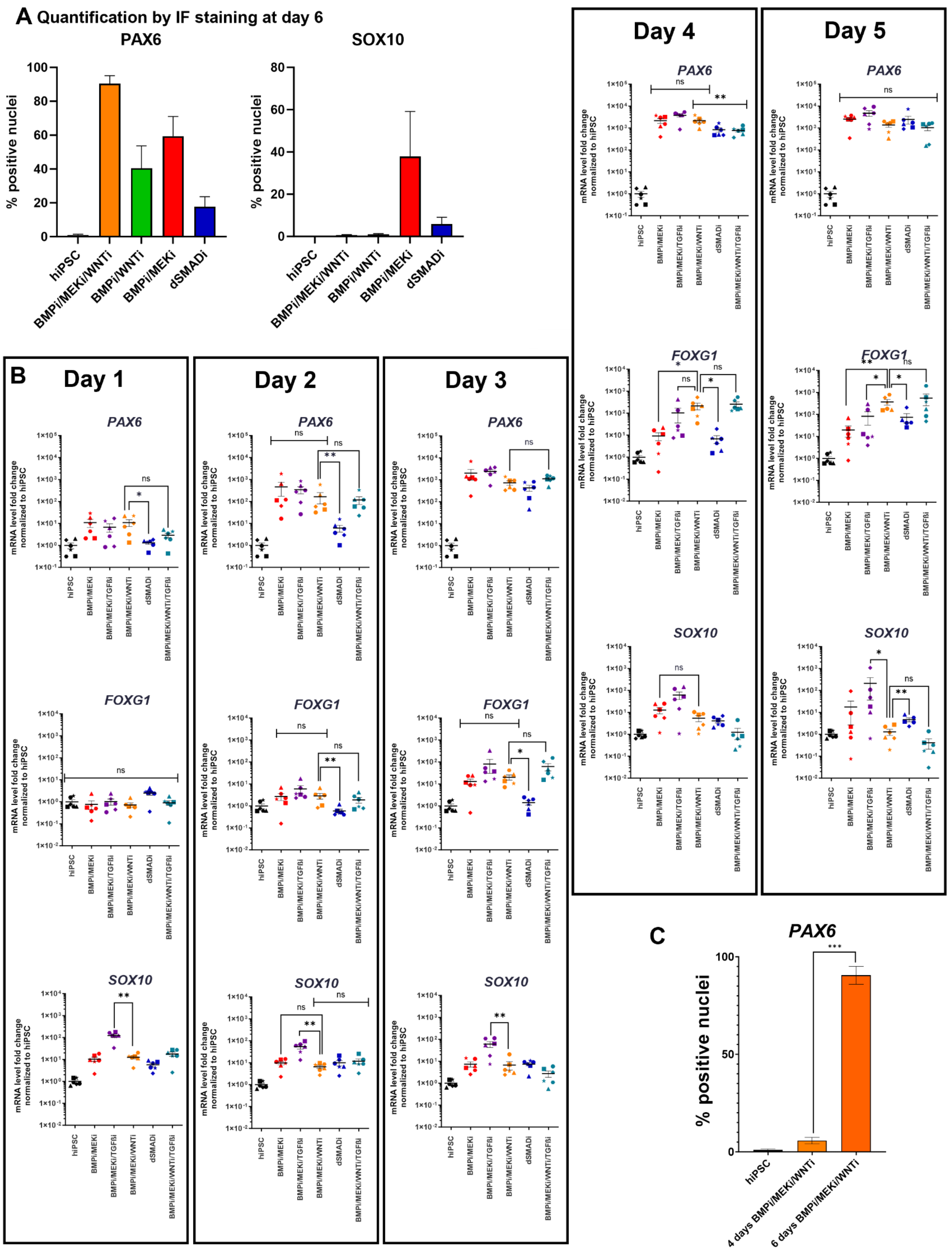

Figure S1: Additional data related to main-text figure 1. A Quantification of IF staining in Figure 1C. 5 different cell lines (hiPSC_1-5). **B** Time course of gene expression measured by qPCR after treatment of hiPSC with different combinations of inhibitors from day 0 to day 6 (results are shown as means ±SEM; N = 6 individual experiments with 6 different cell lines). In the main text Figure 1B, the course over 6 days is shown. The individual results of day 6 are shown in Figure 1D. In order to check significance, the replicates of each day were plotted individually and selected combinations were examined for significance. **C** Quantification of IF images Figure 1E (hiPSC_1-5). (* = $p < 0.05$, ** = $p < 0.01$; ●=hiPSC_1, ▲=hiPSC_2, ■=hiPSC_3, ♦=hiPSC_4, ★=hiPSC_5, ⬣=hiPSC_6)

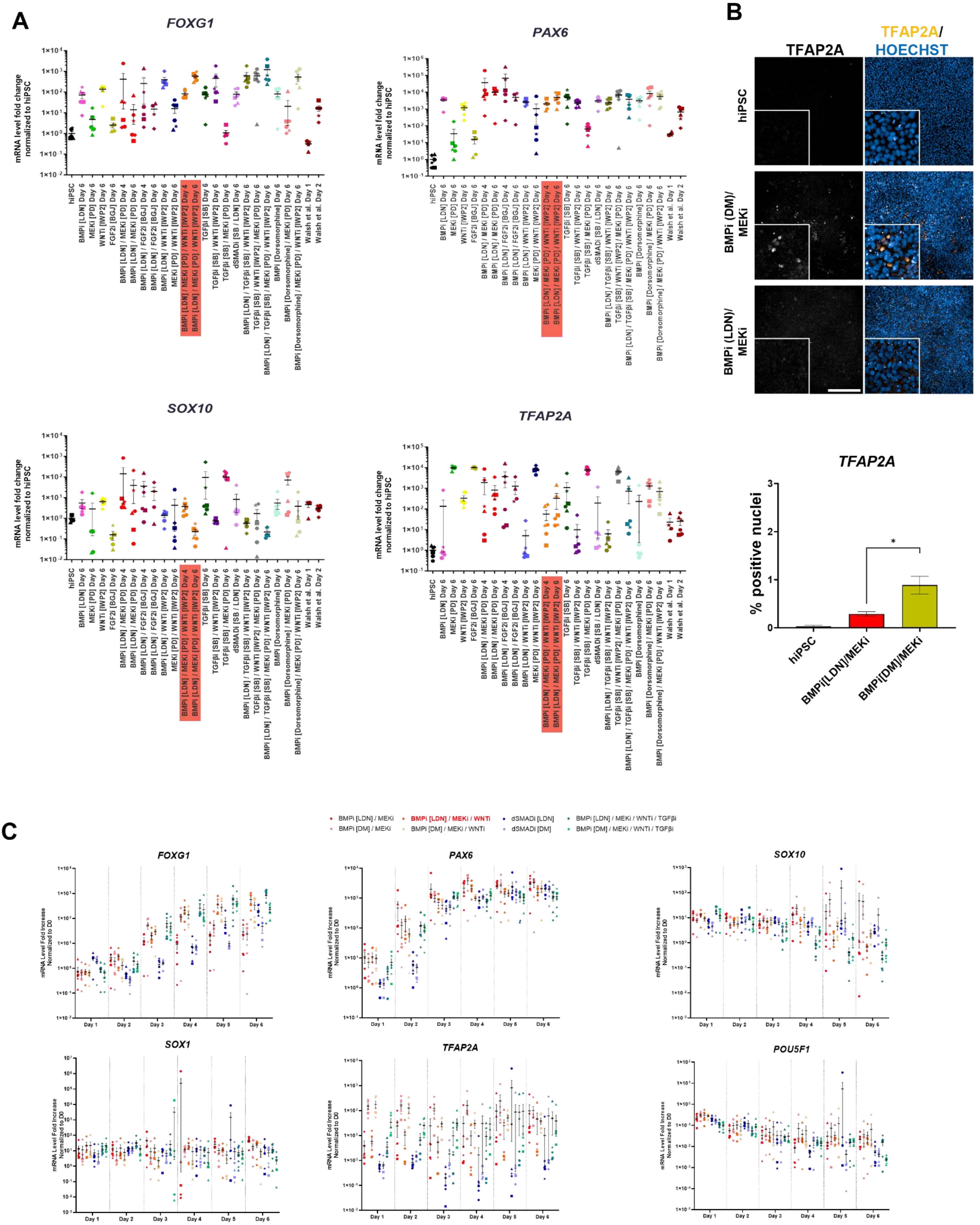

Figure S2: Additional data related to main-text Figure 1. A Gene expression after 4 or 6 days of treatment with different inhibitor combinations. 6 different cell lines (hiPSC_1-6) were used and a total of 6 independent differentiations were performed. For comparison, the differentiation protocol of Walsh et al. was replicated and samples were taken on day 1 and 2 (N=6 cell lines), (results are shown as means ±SEM). **B** Comparison of BMP

inhibition with LDN or DM. Inhibition with DM does not completely block formation of non-neural ectoderm, highlighted by the presence of TFAP2A+ cells. Representative IF pictures of hiPSC_1 were shown. Quantification of TFAP2A positive nuclei in 5 cell lines is shown (hiPSC_1-5, Scale bar: 200 µM, insert 3x zoom-in, means ±SEM). **C** Data points of time course of gene expression measured by qPCR after treatment of hiPSC (hiPSC_1-6) with different combinations of inhibitors from day 0 to day 6 (results are shown as means ±SEM; N = 6 different cell lines; DM=Dorsomorphine). (●=hiPSC_1, ▲=hiPSC_2, ■=hiPSC_3, ♦=hiPSC_4, ★=hiPSC_5, ⬣=hiPSC_6).

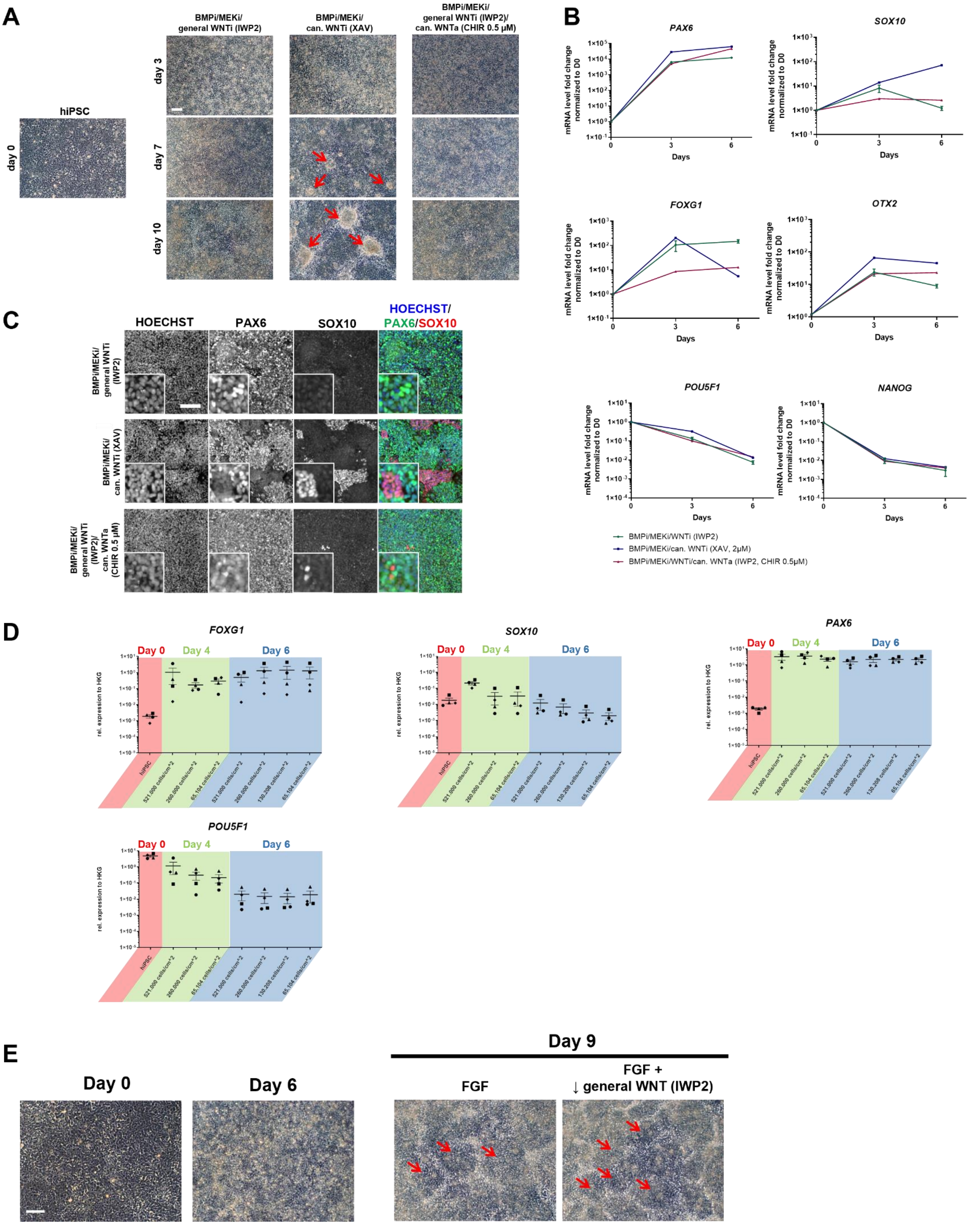

Figure S3: Additional data related to main-text Figure 1. A hiPSC_6 was treated with BMPi/MEKi and either with a general WNT inhibitor (IWP2), a canonical WNT inhibitor (XAV) or IWP2 with slight simultaneous canonical WNT activation (0.5 µM CHIR, GSK3 inhibitor). Phase contrast images of the cells at day 0, day 3, day 7 and day 10 of induction are shown. Sphere formation, signifying neural crest differentiation, is observed only when canonical WNT is inhibited without additional WNT inhibitors. Red arrows indicate spheres (Scale bar: 100 µM). **B** Gene analysis

by qPCR of relevant genes. Inhibition of canonical WNT results in the highest fold increase in *PAX6* and *SOX10*, along with a rapid downregulation of *FOXG1* starting from day 3 onwards. General WNT inhibition leads to the highest fold increase for *FOXG1* (1 hiPSC line was used N=1 (hiPSC_6), BMPi/MEKi/WNTi n=3 independent differentiations, other conditions n=1, results are shown as means ±SEM). **C** IF staining of cells (same experiment as in B) at day 6 of neural induction. Cells were stained for the neuroectodermal marker PAX6 and the neural crest marker SOX10. Patches of SOX10+ cells were observed only when canonical WNT was inhibited without additional WNT inhibitors. Individual SOX10+ cells are also present when general WNT is inhibited and the cells are treated with the canonical WNT activator CHIR. The images suggest that non-canonical WNT signaling has a more significant impact on neural crest formation than canonical WNT signaling (Scale bar: 100 µM, insert 3x zoom-in). **D** hiPSC_1-4 lines were differentiated up to day 6 using the BMWi protocol. On day 0 cells were seeded at different densities. Using qRT-PCR, the RNA samples from the hiPSC, the pre-differentiated precursors and the NPC were examined for relevant genes on the day of replating (N=4 cell lines, results are shown as means ±SEM). **E** hiPSC were induced for 6 days by BMWi. Subsequently, the cells were expanded for 4 days through treatment with FGF2 or a combination of FGF2 and general WNT inhibition (IWP2). Phase contrast images were taken on D0, D6 and D9. Representative images of hiPSC_6 are shown. After treatment with FGF2 formation of neural rosettes can be observed. Selected rosetted labeled by arrows (Scale bar: 100 µM).

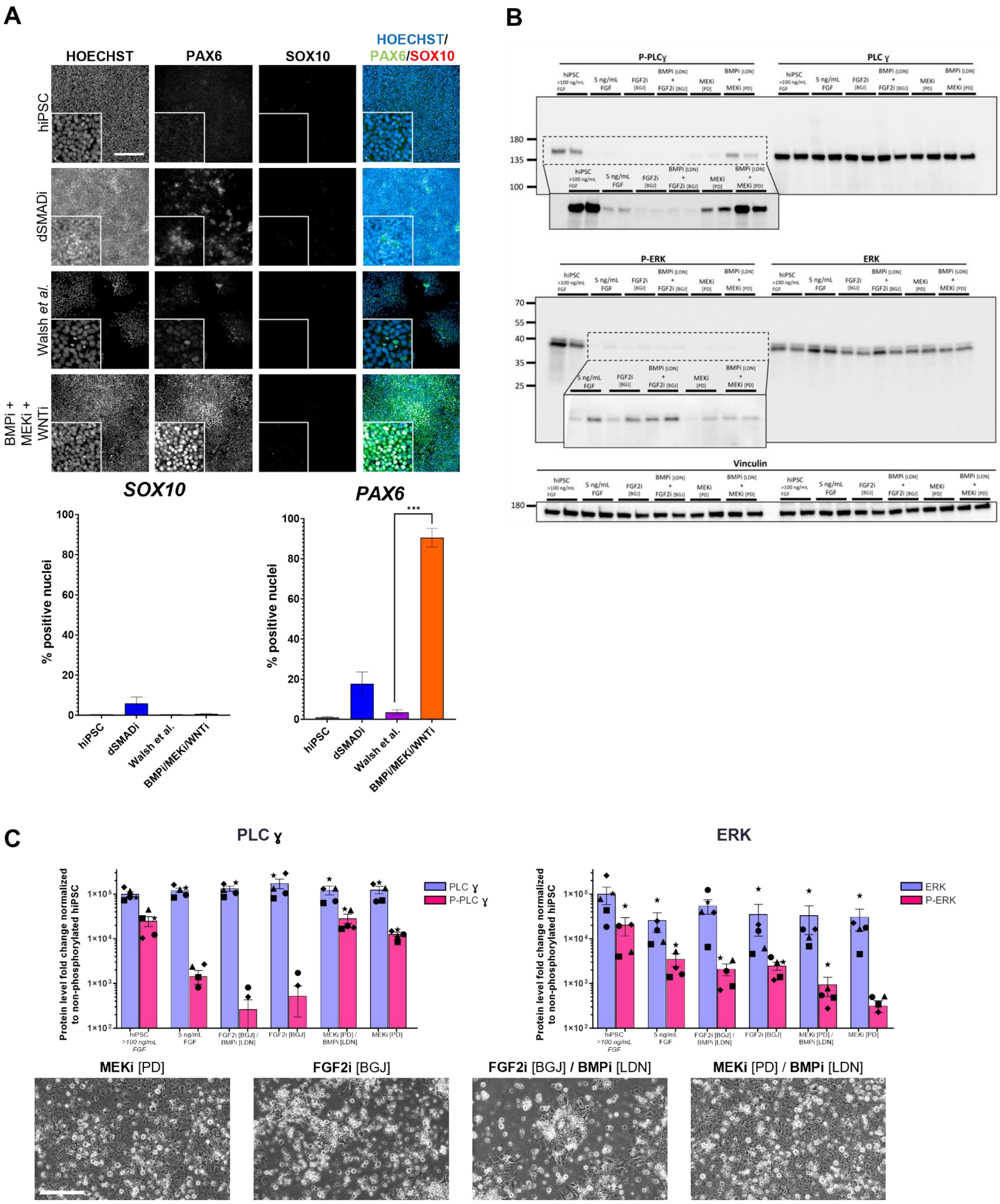

Figure S4: **Additional data related to main-text Figure 1. A** Representative immunofluorescence staining (hiPSC_5) after 6 days of treatment with various inhibitors. According to Walsh et *al.* differentiated cells were fixed on day 2 (final stage NPC) of the protocol instead (Scale bar: 200 µM, insert 3x zoom-in). A quantification of 5 cell lines was carried out (N = 5 cell lines, hiPSC_1-5, results are shown as means ±SEM). **B** hiPSC were treated with the indicated inhibitors for 4 days. As a positive control, 5 ng/ml FGF2 was added to FGF2-free medium, or hiPSC medium (100 ng/ml FGF2) was used. The results of 5 cell lines (hiPSC_1-5) were evaluated using western blot

(12 µg protein each well) with vinculin control. **C** Quantification of western blot results from B (N=5 cell lines). The positive control (>100 ng/ml FGF2) was set to 100% and the other samples were then normalized (results are shown as means ±SEM). Representative images show hiPSC_4 after 4 days of treatment with the specified inhibitors (scale bar: 200 µM).

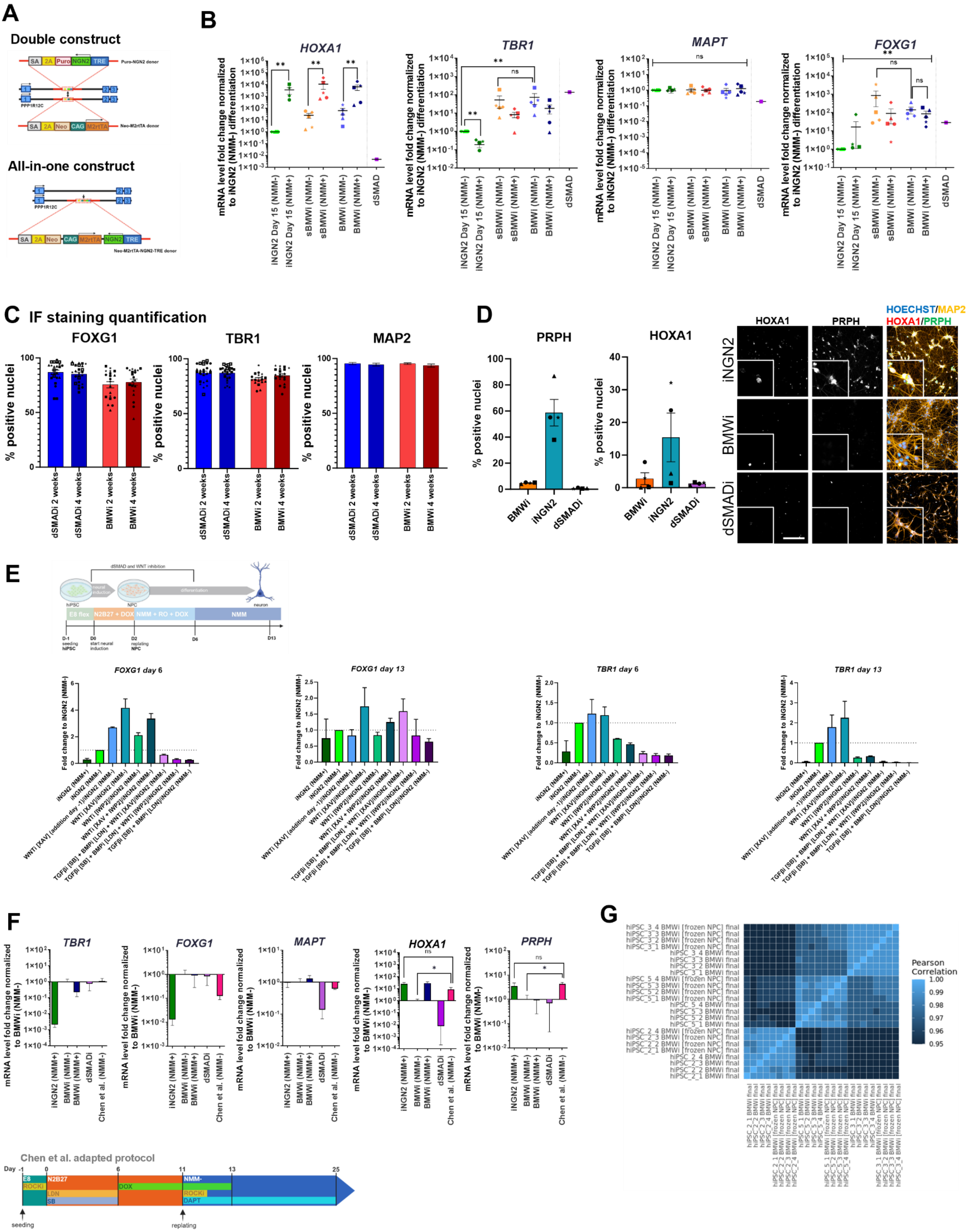

Figure S5: Additional data related to main-text Figure 1/2. A The overexpression of the basic helix-loop-helix transcription factor neurogenin 2 (NGN2) promotes the direct differentiation of hiPSC to neurons. In the ASSV1 (PPP1R12C) locus, puro-NGN2 and neo-M2rtTA were inserted on 2 alleles by gene editing through HDR pathway (hiPSC_1,2,4,5/ Double construct). Alternatively, an all-in-one construct was inserted into an allele in AAVS1

(hiPSC_3 - All-in-one construct). The expression of NGN2 is stimulated by adding DOX. **B** Gene expression at comparable times of different differentiation protocols, normalized to iNGN2 (NMM-) protocol. The final neurons were replated in NMM+ or NMM-. The dSMADi neurons were only replated in NMM+ according to the original protocol. 5 independent differentiations (different cell lines) were carried out (sBMWi, BMWi, iNGN2; hiPSC_1-5) and for comparison 3 cell lines (hiPSC_7-9) with the dSMADi protocol (results are shown as means ±SEM, same data plotted as Figure 2B). **C** Quantification of IF stainings (hiPSC_1-5) BMWi vs dSMADi neurons 2 and 4 weeks after final plating of the respective protocol (N=5 cell lines BMWi protocol, dSMADi additional hiPSC_8, n=4 technical replicates, results are shown as means ±SEM). **D** Quantification of IF stainings and representative images (hiPSC_1). Neurons were matured 2 weeks after final replating of the respective protocol (N=4 cell lines, hiPSC_1-4, scale bar: 200 µm, insert 3x zoom-in, results are shown as means ±SEM). **E** From day 0 (XAV treatment also from day -1) until day 6, differentiation was performed with and without the addition of dSMADi (LDN and SB) and WNT (IWP2 and XAV) pathway inhibitors, alone and in combination, in order to obtain more cortical neurons on day 13 of iNGN2 neuron differentiation (n=3 independent differentiations from hiPSC_1, except NMM+ and dSMADi n=2, results are shown as means ±SEM). **F** hiPSC_1-4 were differentiated according to protocol adapted from Chen et al. 2020 (dSMADi with following NGN2 overexpression, protocol see supplementary Materials and Methods). qPCR comparison with BMWi protocol shows an increased expression of hindbrain and PNS genes in Chen et al. neurons (N=4 cell lines). Schematic representation of the protocol is also shown. **G** hiPSC were differentiated according to the BMWi protocol. On day 8, half of one differentiation was replated and the other half cryopreserved. The replated NPC were matured for 14 days. The frozen NPC were thawed a week later and matured for 14 days (N=3 cell lines hiPSC_2,3,5, n=4 independent differentiations of each cell line). RNA seq was performed and similarity across replicates from frozen and non-frozen NPC was assessed by Pearson correlation coefficient (r = 0.99, N=3 cell lines, n=4 technical replicate).

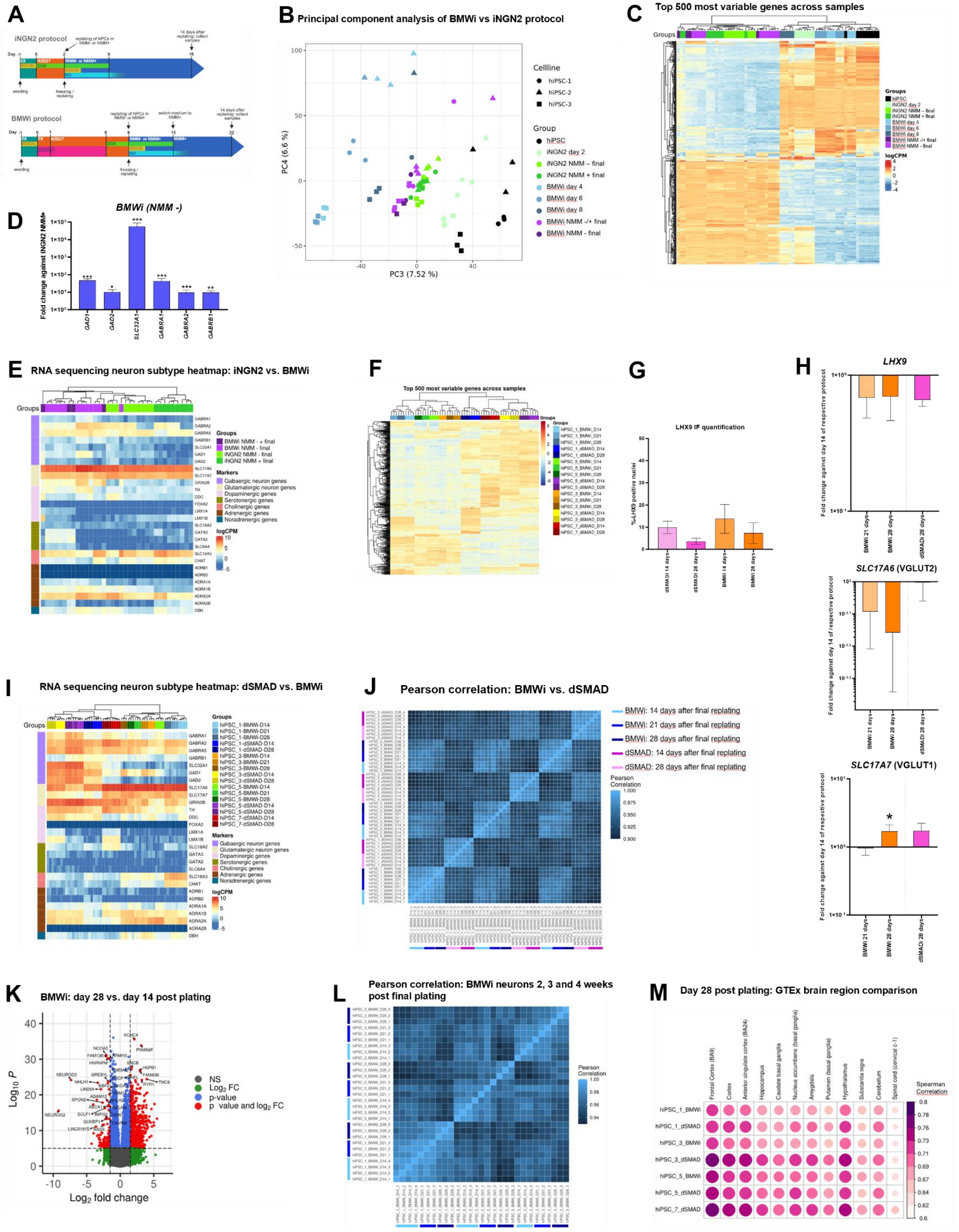

Figure S6: Additional data related to main-text Figure 3. A Schematic BMWi and iNGN2 protocols used for bulk RNA-seq, with respective timepoints of changing NMM media. The neurons were either plated final in NMM + or NMM -. BMWi cells were also replated in NMM- and medium was changed to NMM+ after 7 days (BMWi NMM -/+

final). Sequencing was performed on 3 different cell lines with at least 3 technical replicates (hiPSC_1-3). **B** Principal component analysis of top 500 variable genes shows RNA seq results comparing iNGN2 and BMWi protocol (Figure 3). Shown are PCA dimensions 3 vs. 4 stratifying between different protocols, while first two principal components are not shown as they differentiate cell types. **C** Bulk RNA-seq results showing differences between the iNGN2 and BMWi protocols by hierarchically clustering the 500 most variable genes. **D** Comparison of iNGN2 neurons (NMM+) and BMWi neurons in bulk RNA-seq to show the expression of GABAergic marker genes (results are shown as means ±SEM, * = $p < 0.05$, ** = $p < 0.01$, *** = $p < 0.001$). **E** Heatmap showing hierarchical clustering of BMWi and iNGN2 neurons based on marker genes chosen for different neuronal subtypes (adrenergic, cholinergic, dopaminergic, GABAergic, glutamatergic, noradrenergic and serotonergic genes). Genes not detected across samples were indicated with dark-blue color, at higher intensity than minimum expression levels. **F** Bulk RNA-seq of 2 and 4 weeks matured dSMADi neurons (hiPSC_1,3,5,7, N=4 cell lines, n=3 technical replicates/independent differentiations) compared to 2, 3 and 4 week matured BMWi (hiPSC_1,3,5, N=3 cell lines, n=3 technical replicates/independent differentiations). 500 most variable genes differentiating BMWi and dSMADi. **G** Quantification of IF stainings of LHX9 in 2 and 4 weeks matured dSMADi and BMWi neurons (N=4 cell lines n=2 technical replicates each, results are shown as means ±SEM). **H** Comparison of BMWi and dSMADi bulk RNA-seq timecourse against the respective protocol of gene expression levels during maturation of neurons (N=4 cell lines, hiPSC_1-4, results are shown as means ±SEM, * = $p < 0.05$). **I** Heatmap of bulk transcriptomics at different maturation times of BMWi and dSMADi neurons. Same marker genes are shown as in Figure S6D. Genes not detected across samples were indicated with dark-blue color, at higher intensity than minimum expression levels. **J** Pearson correlation of BMWi and dSMADi bulk RNA-seq replicates. **K** Volcano plot of 4 vs. 2 weeks matured BMWi neurons. **L** Pearson correlation of different maturation timepoints of BMWi neurons (2, 3 and 4 weeks after replating). Legend see I. **M** GTEx brain region analysis of BMWi and dSMADi neurons 28 days after final replating of the respective protocol. Color-intensity and dot size indicate Spearman correlation coefficient.

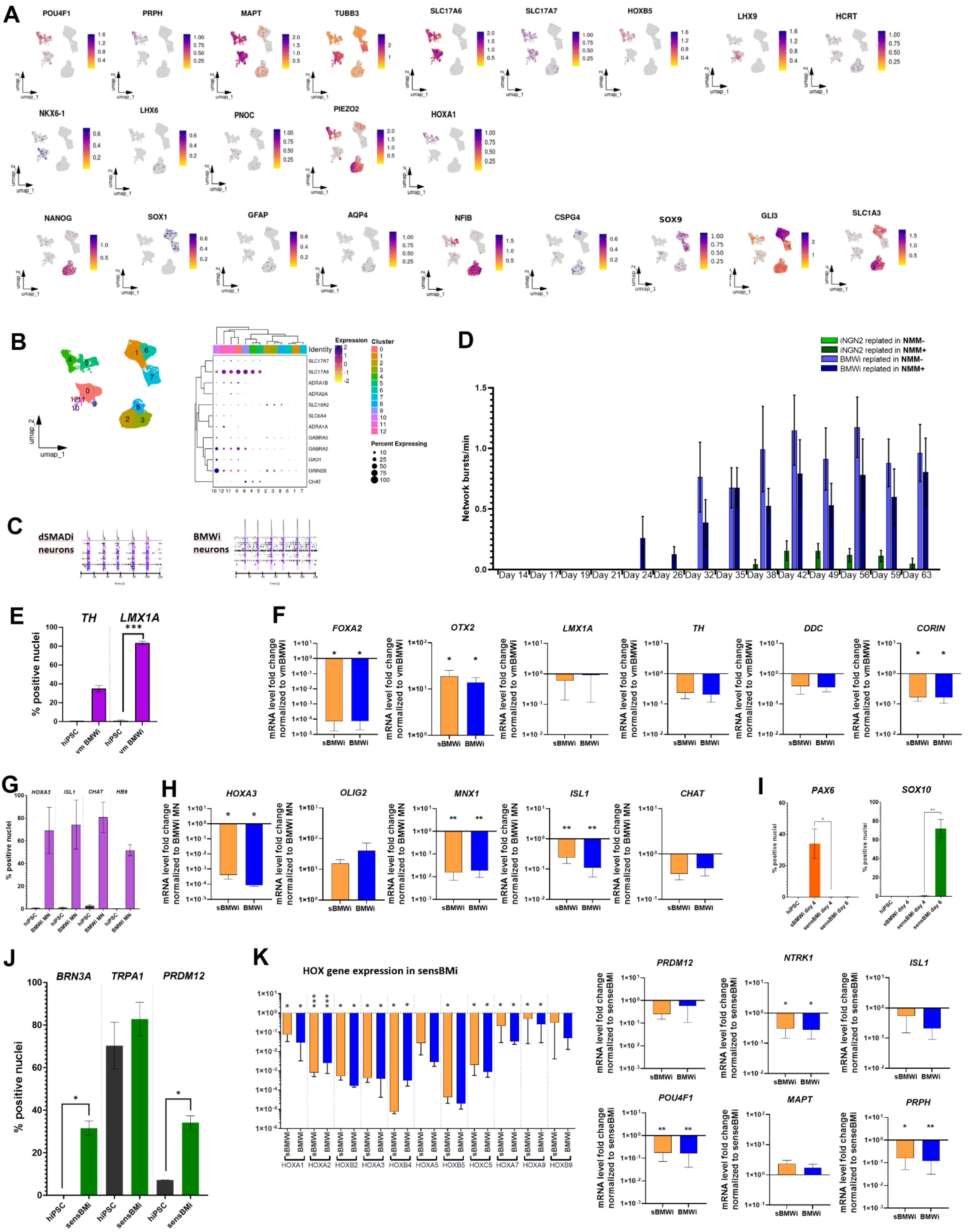

Figure S7: Additional data related to main-text Figures 3/6/7. A Additional analysis of marker genes of snRNA-seq Figure 3E. **B** Clustering of BMWi neurons, BMWi NPC day 6, iNGN2 neurons and hiPSC (hiPSC_3) in snRNA-seq. A bubble plot of marker genes expressed in different clusters is shown. **C** Network activity pattern of MEA measurements of dSMADi neurons and BWMi neurons. 2-minute sections are shown (day 28 after final replating,

hiPSC_1). **D** Determination of the beginning of the electrophysiological activity of the iNGN2 and BMWi neuron cultures. Medium of the neurons replated in NMM- was switched to NMM+ after 7 days. The number of bursts (burst duration min 250 spikes) is plotted against the days after the final replating. Experiment was carried out with 5 different cell lines (hiPSC_1-5) each with 6 technical replicates (results are shown as means ±SEM). The activity of the neurons was recorded for 8 min. **E** Quantification of mDAN marker genes LMX1A and TH of 14 days matured ventral midbrain BMWi (vmBMWi) (Figure 6C, N=4 cell lines, hiPSC_1-4, means ±SEM) **F** Fold change of sBMWi and BMWi neurons 14 days after final replating against vmBMWi 14 days after replating (N=4 cell lines, hiPSC_1-4, all results normalized to vmBMWi of each cell line, means ±SEM). **G** Quantification of marker genes of 15 days matured BMWi MN (Figure 6F, N=4 cell lines, hiPSC_1-4, means ±SEM) **H** Fold change of sBMWi and BMWi neurons 14 days after final replating against BMWi MN 14 days after replating. (N=3 cell lines, hiPSC_1-3, all results normalized to BMWi MN of each cell line, means ±SEM) **I** Quantification of results shown in Figure 7B (N = 2 cell lines, hiPSC_1,5, n=3, n_{hiPSC}=2, means ±SEM). **J** Quantification of data shown in Figure 7C **(**N = 2 cell lines, hiPSC_1,5, n=4, n_{hiPSC}=1, means ±SEM) **K** Fold change of sensBMi neurons normalized to sBMWi and BMWi neurons 14 days after final replating. (* = $p < 0.05$, ** = $p < 0.01$, *** = $p < 0.001$)

Note S1 (Figure S2/3):

It should be noted that the more potent BMP inhibitor LDN provided a stronger BMPi than Dorsomorphin, which is why we chose LDN in this protocol (Figure S2). Incomplete BMPi was assessed by a stronger expression of *TFAP2A* and consistently with the formation of non-neural ectoderm, the use of Dorsomorphin also resulted in non-neuronal, Cytokeratin 18 (CK18) positive cells (not shown). These cells were not observed in conditions with LDN as inhibitor.

Interestingly, a commonly used alternative WNT inhibitor, XAV939 (“XAV”), inhibiting tankyrase, and thus canonical WNT signaling (Figure 1A), did not succeed in preventing the formation of neural crest clusters (SOX10+ cells), whereas addition of canonical WNT activator CHIR99021 (GSK3β inhibitor) with BMWi led to the formation of only few SOX10+ cells (Figure S3A/B/C).

In the dSMADi dependent protocols low cell density leads to a differentiation skewed towards neural crest rather than CNS (Chambers et al., 2009; Münst et al., 2018), implying the use of very high cell densities (Manos et al., 2022). We tested the effect of cell seeding density on the expression of *PAX6, FOXG1 SOX10* and *OCT4*, and found no negative effect of low seeding density (Figure S3D). To compromise between obtaining as many cells as possible from a cell culture vessel and preventing cells from starvation over the course of a weekend, we used the density of 36.000 cells/cm^2 below, unless stated differently.

Note S2 (Figure S4):

We carried out the differentiation described by (Walsh et al., 2020) with the indicated cell lines. In all 6 lines, an increase of *PAX6* and *FOXG1* expression was obtained at the end of the induction period (Walsh et al. 2 days, BMWi 4 or 6 days), but the levels were 7.3-fold lower for *PAX6* and 34.5-fold lower for *FOXG1* (Figure S2A, Figure S4A). It should be noted that Walsh and colleagues used a different inhibitor for FGF2-i, BGJX398 “BGJ” that inhibits the FGF2 receptor rather than the downstream signaling pathways, such as the MAPK/ERK pathway. Another FGF2 dependent signaling pathway, the PLCγ/Ca^{2+} signaling pathway, is associated with the stimulation of neurite growth, but also cell survival. We probed both signaling pathways by measuring the ratio between the unphosphorylated and phosphorylated protein and observed a strong effect of BGJ on both, whereas PD mostly targeted MAPK/ERK, even to an apparently stronger extend than BGJ (Figure S4B/C). We also experienced cell death through inhibition with BGJ or BGJ in combination with BMPi (Figure S4C as an example). That could explain why PD accelerated neural induction without affecting cell survival (Doherty and Walsh, 1996; ZHANG and LIU, 2002).

Note S3 (Figure S5E):

We evaluated XAV alone, added to the culture one day before iNGN2 induction, dSMADi alone, dSMADi with IWP2, IWP2 alone and IWP2 in combination with XAV, together with the iNGN2, replated in NMM- (Figure S5E). We did not observe a strong increase of *FOXG1* or *TBR1* expression on mRNA level at 6 or 13 days of the experiment. The strongest effect on the neuron cultures was achieved with XAV alone, whereas the combination with dSMADi was not beneficial, especially for TBR1 expression. Nevertheless, the increase of *FOXG1* expression in the late timepoint was only 1.5-fold over the iNGN2 alone induction.

Supplementary experimental procedures

hiPSC culture conditions (related to main text materials & methods)

Table S1: *List of cell lines.*

Name	Referred as	Source/ Modification	Disease genotype	Sex	Age	Reprogramming Method	Modification
**BIONi010-C-13**	hiPSC_1 ●	EBiSC*	Control cell line	male	15-19	Episomal Reprogramming factors: KLF4, Lin28, MYC, POU5F1, shP53, SOX2	edited using CRISPR at CRO (Bioneer) with a DOX-inducible NGN2 overexpression construct introduced in the AAVS1 alleles (one allele CAG:m2rtTA, other allele TRE:NGN2)
**BIOMEDi004-A (Coriell Fibroblast AG08125)**	hiPSC_2 ▲	BioMedX/ Bioneer	sAD	male	64	RNA based reprogramming (Schöndorf et al., 2019), Reprogramming factors: OSKM	see hiPSC_1
**iPSC0028 MAPT TM (iNGN2 insert)**	hiPSC_3 ■	EBiSC/ internal edit/ Bioneer	iPSC0028 with MAPT P301S/ E10+14/ E10+16, all biallelic	female	24	see hiPSC_2	edited using CRISPR at CRO with a DOX -inducible NGN2 overexpression construct containing both, CAG:m2rtTA and TRE:NGN2 in one cassette introduced in one AAVS1 allele
**BIOMEDi001-A (Coriell Fibroblast AG08379)**	hiPSC_4 ♦	BioMedX/ Bioneer	Control cell line	female	60	see hiPSC_2	see hiPSC_1
**BIOMEDi005-A (Coriell Fibroblast AG06869)**	hiPSC_5 ★	BioMedX/ Bioneer	sAD	female	60	see hiPSC_2	see hiPSC_1
**SBAD3 Cl1**	hiPSC_6 ⬣	IMI StemBANCC	Control cell line	female	31	Sendai virus, Reprogramming factors: OSKM	-
**iPSC0028 MAPT WT**	hiPSC_7 ▼	Sigma Aldrich	Control cell line	female	24	Retroviral, Reprogramming factors: OSKM	-
**iPSC0028 MAPT TM**	hiPSC_8 □	EBiSC/ internal edit	MAPT P301S/ E10+14/ E10+16, all biallelic	female	24	see hiPSC_7	-
**iPSC0028 MAPT KO**	hiPSC_9 ○	Sigma Aldrich/ internal edit	Knockout of MAPT	female	24	see hiPSC_7	-

Western Blots (related to Figure S4)

Cells were lysed on ice for 10 min with RIPA buffer (ThermoFisher) supplemented with Halt Protease & Phosphatase Inhibitor Cocktail (ThermoFisher). The lysate was then centrifuged and the supernatant taken. Protein concentration was determined via BCA (Pierce BCA Protein Asay Kit, ThermoFisher, 23225) and 15 µg protein loaded onto a gel (Criterion TGX, 4-20%, BIO-RAD, 5671095). The quantitative analyse were performed with Image Lab 6.0.

Table S2: *Primary antibodies for western blot.*

1st antibody	Company	Order number	Dilution
P44/42 MAPK (ERK1/2) (137F5) Rabbit mAb	Cell Signaling	4695	1:950
Phospho-p44/42 (ERK1/2) (Thr202/Tyr204) (D13.14.4E) XP Rabbit mAb	Cell Signaling	4370	1:950
PLCγ1 (D9H10) XP Rabbbit mAb	Cell Signaling	5690	1:950
Phospho-PLCγ1 (Tyr783) (D6M9S) Rabbit mAb	Cell Signaling	14008	1:950
Recombinant Anti-Vinculin antibody [EPR19579]	Abcam	Ab207440	1:10,000

Table S3: *Secondary antibody for western blot.*

2nd antibody	Company	Order number	Concentration	Dilution
Goat Anti-Rabbit IgG H&L (HRP)	Abcam	ab97051	1 mg/mL	1:10,000

Neural induction (related to main text materials & methods)

Table S4: *N2B27 medium.*

<table>
<tr><th>Ingredient</th><th>Concentration</th><th>Source</th></tr>
<tr><td>DMEM/F12</td><td>0.5x</td><td rowspan="6">ThermoFisher</td></tr>
<tr><td>Neurobasal medium</td><td>0.5x</td></tr>
<tr><td>N2 supplement</td><td>0.5x</td></tr>
<tr><td>B27 supplement (w/o vitamin A)</td><td>0.5x</td></tr>
<tr><td>Penicillin-Streptomycin
(5,000 U/mL)</td><td>50 U/mL</td></tr>
<tr><td>GlutaMax</td><td>0.5 mM</td></tr>
</table>

Table S5: *Neural maturation medium (NMM).*

Medium	Ingredient	Concentration	Source
**NMM+**	Neurobasal plus medium	1x	ThermoFisher
**NMM+**	B27 Supplement plus supplement	1x	ThermoFisher
**NMM-**	Neurobasal medium	1x	ThermoFisher
**NMM-**	*B27 Supplement w/o vitamin A*	1x	ThermoFisher
**NMM+ and NMM-**	GlutaMax	1 mM	ThermoFisher
	Penicillin-Streptomycin (5,000 U/mL)	50 U/mL	ThermoFisher
	BDNF	10 ng/mL	Biotechne
	GDNF	5 ng/mL	Biotechne
	dcAMP	200 µM	Merck
	L-Ascorbic acid	200 µM	Merck
	Laminin	2 µg/mL	Merck

Immunofluorescence staining (related to all IF stainings in main text)

In this supplementary following antibody was used additionally to the antibodies in main text.

Table S6: *Supplementary antibodies.*

Target	Name	Stock concentration	Dilution	Source	Identifier
**FOXG1**	Anti FOXG1 antibody - ChIP Grade	1 mg/mL	1:150	Abcam	ab18259
**MAP2**	Anti-MAP2 antibody	19 mg/mL	1:10 000	Abcam	ab5392
**Tau aggregates/ MC1**	Anti MC1 antibody	12.4 mg/mL	1:5000	Peter Davies Lab	-
**PAX6**	Pax6, bioreactor supernatant	500 µg/L	1:60 - 1:80	DSHB	AB 528427
**EN1**	4G11, concentrate	466 µg/mL	5 µg/mL	DSHB	AB_2314371
**CHAT**	Anti-Choline Acetyltransferase Antibody	n/a	1:100	Merck	AB144P
**HB9**	81.5C10, bioreactor supernatant	430 µg/mL	1:86	DSHB	AB_2145209
**HOXA3**	HOXA3 Polyclonal Antibody	0.2 mg/mL	1:100	ThermoFisher	PA5-56077
**TH**	Tyrosine Hydroxylase Antibody	n/a	1:5 000	Immunostar	22941
**FOXA2**	Recombinant Anti-FOXA2 antibody [EPR4466]	n/a	1:300	Abcam	ab108422
**ISL1**	40.2D6, concentrate	234 µg/mL	1:47	DSHB	AB_528315
**TRPA1**	TRPA1 Antibody – BSA free	1 mg/mL	1:200	Biotechne	NB110-40763

Target	Name	Stock concentration	Dilution	Source	Identifier
**PRDM12**	PRDM12 Polyclonal Antibody	1 mg/mL	2 µg/mL	ThermoFisher	PA5-60257
**VGLUT2**	VGLUT2 (D7D2H) Rabbit mAb	n/a	1:100	Cellsignaling	71555
**VGLUT1**	VGLUT1 (E9D2B) Mouse mAB	n/a	1:800	Cellsignaling	98199
**Cytokeratin 18**	Anti-Cytokeratin 18 antibody ab52948	1mg/mL	3.3µg/mL	Abcam	ab52948
**PRPH**	Alexa Fluor® 488 Anti-Peripherin antibody [EPR23445-28]	n/a	1:400	Abcam	ab275150
**LHX9**	LHX9 Polyclonal Antibody	1.41 mg/mL	1:150	ThermoFisher	PA5-88722
**HOXA1**	Anti-HOXA1 antibody	n/a	1:100	Abcam	ab168179

Brain region analysis (related to Figure S6)

The data used for the brain region analysis described in this manuscript were obtained from the GTEx Portal on 01/04/2023 as median gene-level TPMs by tissues (v8, RNASeQCv1.1.9) (Lonsdale et al., 2013). For selected brain region specific tissues and spinal chord tissue, Spearman correlation coefficients were assessed between mean expression levels per sample group of BMWi/dSMADi protocol data and GTEx expression. The R package corrplot (v0.92) (Wei and Simko, 2021) was used to visualize results with circle size and color intensity encoding correlation coefficients.

Alternative coating method with Laminin (related to Figure S3)

Cell culture dishes were coated with 5 µg/mL LN521 (BioLamina) diluted in DPBS (+/+). 80 µL coating solution was added to each 96 well and 2 mL to each 6 well. Dishes were sealed with parafilm and stored at 4°C on a shaker for a minimum of 2 hours. The dishes can be stored in the fridge up to 3 weeks. After one week in the fridge, the coating solution needed to be replenished with DPBS (+/+) to prevent drying during long-term storage. This method was used in Figure S3A-C, E.

Reproduction of Walsh *et al*. (related to Figure S5)

All experiments were carried out on vitronectin-coated plates. IPSCs were cultured for 4 days until they were confluent and passaged with hypertonic citrate buffer and replated in E8

medium + 10 µM ROCKi. 24 h after passage, the medium was changed to E6 supplemented with 500 nM LDN and 100 nM BGJ. 24 h later a complete medium change was carried out with E6 medium supplemented with 500 nM wntC59, 20 ng/mL FGF2 and 500 nM A8301. The final samples were taken 24 h after the last medium change.

Reproduction of Nehme *et al*. (related to Figure S5)

100,000 cells/well were plated on Matrigel coated 6 well plates in E8 flex medium + 10 µM ROCKi. On day 0 medium was changed to N2B27, 2 µg/mL DOX, 100 nM LDN, 10 µM SB and 2 µM XAV. Day 1 a complete medium change with N2B27, 2 µg/mL DOX, 100 nM LDN, 10 µM SB and 2 µM XAV was performed. After 3 days, the cells were replated as described in main text materials and methods and replated in NMM-, DOX, LDN, SB and XAV, 10 µM ROCKi and 500 nM RO. The day after, 90% of the medium was replaced to NMM-, 2 µg/mL DOX, LDN, SB and XAV, and RO to remove ROCKi. On day 6, medium was replaced with NMM+ medium. The medium was 50% replaced every 4-5 days.

Reproduction of Chen *et al*. (related to Figure S5)

250.000 cells were plated in each 6 well in E8flex medium + 10 µM ROCKi. Day 0 medium was changed to N2B27 supplemented with 100 nM LDN + 10 µM SB. A complete medium change was performed each day until day 6. Day 6 medium was changed to N2B27 + 2 µg/mL DOX. After 5 days the cells were replated as in main text materials and methods described in NMM- + DOX + 2.5 µM DAPT (Tocris) + ROCKi. After 2 days DOX was removed from the medium. The medium was 50% replaced every 4-5 days. 14 days after final replating the neuons were lysed.

Quantitative real-time PCR (related to all PCR experiments of this manuscript)

Table S7: Primer real-time PCR.

Gene	Company	Assay ID
CHAT	Thermo Fisher	Hs00758143_m1
CORIN	Thermo Fisher	Hs00198141_m1
DCC	Thermo Fisher	Hs00180437_m1
FOXA2	Thermo Fisher	Hs00936490_m1
FOXG1	Thermo Fisher	Hs01850784_s1
GAPDH	Thermo Fisher	Hs99999905_m1
HOXA1	Thermo Fisher	Hs00939046_m1
HOXA2	Thermo Fisher	Hs00534579_m1
HOXA3	Thermo Fisher	Hs00601076_m1
HOXA7	Thermo Fisher	Hs00600844_m1
HOXA9	Thermo Fisher	Hs04931836_m1
HOXB2	Thermo Fisher	Hs01911167_s1

Gene	Company	Assay ID
HOXB4	Thermo Fisher	Hs00256884_m1
HOXB5	Thermo Fisher	Hs00357820_m1
HOXB9	Thermo Fisher	Hs00256886_m1
HOXC5	Thermo Fisher	Hs00232747_m1
IRX3	Thermo Fisher	Hs01124217_g1
ISL1	Thermo Fisher	Hs00158126_m1
LMX1A	Thermo Fisher	Hs00892663_m1
MAPT	Thermo Fisher	Hs00902192_m1
MNX1	Thermo Fisher	Hs00907365_m1
NANOG	Thermo Fisher	Hs04399610_g1
NKX2.1	Thermo Fisher	Hs00968940_m1
NTRK1	Thermo Fisher	Hs01021011_m1
OLIG2	Thermo Fisher	Hs00300164_s1
OTX2	Thermo Fisher	Hs00222238_m1
PAX3	Thermo Fisher	Hs00992437_m1
PAX6	Thermo Fisher	Hs01088114_m1
POU4F1	Thermo Fisher	Hs00366711_m1
POU5F1	Thermo Fisher	Hs04260367_gH
PPIA	Thermo Fisher	Hs04194521_s1
PRPH	Thermo Fisher	Hs00986945_g1
PRDM12	Thermo Fisher	Hs00964106_m1
RPL13	Thermo Fisher	Hs00744303_s1
SOX1	Thermo Fisher	Hs01057642_s1
SOX10	Thermo Fisher	Hs00366918_m1
TBR1	Thermo Fisher	Hs00232429_m1
TFAP2A	Thermo Fisher	Hs01029413_m1
TH	Thermo Fisher	Hs00165941_m1

Single nuclei RNA sequencing (related to Figure 3 and S7)

Table S8: Lysis buffer for snRNA seq.

Ingredient	Concentration	Source
**NP40 (25%**)	0.1%	Thermo Fisher
**NaCl (1 M**)	10 mM	Merck
**$MgCl_2$ (1 M**)	3 mM	Merck
**Tris-HCl (pH 7.4, 1 M**)	10 mM	Merck
**DPBS (-/-)**	-	Thermo fisher

Table S9: Washing buffer for snRNA seq.

Ingredient	Concentration	Source
35% BSA solution	1%	Merck
Protector RNase inhibitor	0.2 U/µL	Roche
DPBS (-/-)	-	Thermo Fisher

Supplementary references:

Chambers, S.M., Fasano, C.A., Papapetrou, E.P., Tomishima, M., Sadelain, M., and Studer, L. (2009). Highly efficient neural conversion of human ES and iPS cells by dual inhibition of SMAD signaling. Nat Biotechnol *27*, 275–280. https://doi.org/10.1038/nbt.1529.

Doherty, P., and Walsh, F.S. (1996). CAM-FGF Receptor Interactions: A Model for Axonal Growth. Mol Cell Neurosci *8*, 99–111. https://doi.org/10.1006/mcne.1996.0049.

Lonsdale, J., Thomas, J., Salvatore, M., Phillips, R., Lo, E., Shad, S., Hasz, R., Walters, G., Garcia, F., Young, N., et al. (2013). The Genotype-Tissue Expression (GTEx) project. Nat. Genet. *45*, 580–585. https://doi.org/10.1038/ng.2653.

Manos, J.D., Preiss, C.N., Venkat, N., Tamm, J., Reinhardt, P., Kwon, T., Wu, J., Winter, A.D., Jahn, T.R., Yanamandra, K., et al. (2022). Uncovering specificity of endogenous TAU aggregation in a human iPSC-neuron TAU seeding model. Iscience *25*, 103658. https://doi.org/10.1016/j.isci.2021.103658.

Münst, S., Koch, P., Kesavan, J., Alexander-Mays, M., Münst, B., Blaess, S., and Brüstle, O. (2018). In vitro segregation and isolation of human pluripotent stem cell-derived neural crest cells. Methods *133*, 65–80. https://doi.org/10.1016/j.ymeth.2017.09.012.

Walsh, P., Truong, V., Nayak, S., Montivero, M.S., Low, W.C., Parr, A.M., and Dutton, J.R. (2020). Accelerated differentiation of human pluripotent stem cells into neural lineages via an early intermediate ectoderm population. Stem Cells *38*, 1400–1408. https://doi.org/10.1002/stem.3260.

Wei, T., and Simko, V. (2021). R package “corrplot”: Visualization of a Correlation Matrix (Version 0.92). https://github.com/taiyun/corrplot.

ZHANG, W., and LIU, H.T. (2002). MAPK signal pathways in the regulation of cell proliferation in mammalian cells. Cell Res *12*, 9–18. https://doi.org/10.1038/sj.cr.7290105.
